# FAP-Inhibitor Homodimers with Engineered Linker-Spacer Units: Toward Optimized Radiopharmaceuticals

**DOI:** 10.1021/acsomega.5c13192

**Published:** 2026-08-11

**Authors:** Naveen Kumar, Marcel Martin, Adrianna Bilinska, Elena Menéndez, Ingrid De Meester, Frank Roesch, Axel Rominger, Eleni Gourni

**Affiliations:** † Department of Nuclear Medicine, Inselspital, Bern University Hospital, University of Bern, Bern 3010, Switzerland; ‡ Department of Chemistry—TRIGA Site, Johannes-Gutenberg University, Mainz 55128, Germany; § Graduate School of Cellular and Biomedical Sciences, University of Bern, Bern 3010, Switzerland; ∥ Department of Pharmaceutical Sciences, Laboratory of Medical Biochemistry, University of Antwerp, Wilrijk-Antwerp 2610, Belgium

## Abstract

Monomeric fibroblast activation protein inhibitors (FAPi) based PET tracers have demonstrated very suitable imaging characteristics preclinically in various cancer models. More recently, homodimeric FAP inhibitors (FAPi dimers), that consist of two identical FAPi targeting vectors, have been developed, offering prolonged tumor retention and thus enhancing the potential of targeted radioligand therapy (TRT) with long-lived therapeutic radionuclides such as ^177^Lu. Building on the promising results of DOTAGA.Glu.(FAPi)_2_ in preclinical and patient studies, we synthesized a series of novel homodimeric compounds incorporating different linker and spacer units (NPyr-, Glu_2_-, and PEG_2_.Glu-linked dimers), each coupled to either DOTAGA or DO3A as the chelator. Radiolabeling with ^68^Ga and ^177^Lu resulted in high to quantitative radiochemical conversion and purity. All FAPi homodimers exhibited high hydrophilicity, excellent stability in both human serum and PBS and showed subnanomolar FAP affinity with high selectivity over PREP (prolyl endopeptidase) and DPP4 (dipeptidyl peptidase 4). In parallel, saturation binding studies were performed to determine the affinity of the corresponding ^68^Ga-labeled Glu_2_- and PEG_2_.Glu-linked DOTAGA derivatives and exhibited high FAP affinity (*K*_d_: 1.1–1.2 nM). Internalization was rapid, with up to 95% of total cell-bound activity internalized within 30 min in CAFs (cancer-associated fibroblasts). Metabolic studies confirmed that ^68^Ga-labeled radioligands remained stable in plasma, and PET quantification demonstrated favorable pharmacokinetics. Overall, these findings highlight the new FAPi homodimers as promising candidates for efficient ^177^Lu-based TRT. Further *in vivo* studies are warranted to comprehensively assess their pharmacological properties for therapeutic application.

## Graphical abstract

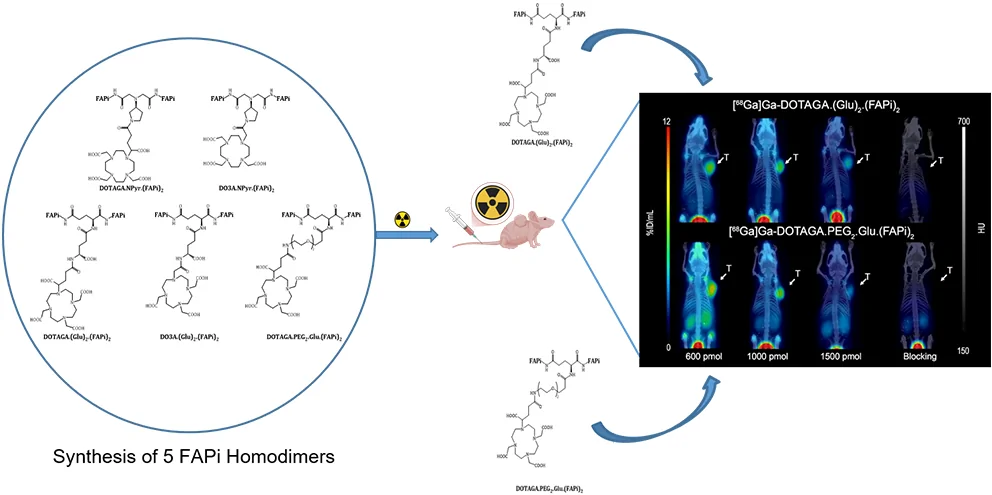

## Introduction

The emergence of fibroblast activation protein inhibitors (FAPi) has introduced a powerful new class of radiopharmaceuticals in nuclear medicine, with transformative potential for both oncological imaging and targeted radionuclide therapy (TRT).^1,2^ Fibroblast activation protein α (FAP) is a membrane-bound serine protease predominantly expressed by cancer-associated fibroblasts (CAFs), which constitute a key component of the tumor stroma in more than 90% of epithelial malignancies including pancreatic, lung, colon, prostate cancer, and others.^3^ FAPi-based radioligands predominantly accumulate in the tumor stroma by targeting CAFs; however, in certain tumor contexts, they may also bind to FAP expressed on the cell membrane. Because CAFs are abundantly expressed in the stroma of most epithelial tumors and relatively absent in healthy tissues; FAPi radioligands enable tumor-specific imaging with high-contrast.^1–3^

The initial generation of FAPi radiopharmaceuticals, primarily monomers, have demonstrated effective tumor targeting, rapid blood clearance, and favorable tumor-to-background contrast in preclinical and clinical studies.^4–31^ Despite these promising attributes, monomeric FAPi tracers often exhibit limited tumor retention, necessitating strategies to prolong tumor residency.^5,14–16,30^ Long tumor retention appears to be an important requirement for efficient targeted radionuclide therapy (TRT) with more long-lived radionuclide like ^177^Lu (*t*_1/2_ = 6.7 d) or targeted alpha therapy with ^225^Ac (*t*_1/2_ = 9.9 d) to deposit a sufficient radiation dose to the tumor.

Currently, there are several strategies under investigation to extend the tumor retention time of FAPi radiopharmaceuticals, such as *e.g.*, using FAP-affine peptides instead of inhibitors or adding an albumin-binding moiety to the FAPi-based radiopharmaceuticals.^22,32–35^ In parallel, tumor retention time was improved drastically by designing FAPi homodimers (or multimers) such as the first-generation linear homodimer DOTAGA.(SA.FAPi)_2_ as published by Moon et al.^23,36^ Initial clinical results with [^177^Lu]­Lu-DOTAGA.(SA.FAPi)_2_, were promising, showing lesion accumulation patterns that closely matched those seen with [^68^Ga]­Ga-DOTA.SA.FAPi PET/CT imaging and persisting at the tumor sites for several days.^8,30^ However, significant improvements were achieved with the next-generation homodimer DOTAGA.Glu.(FAPi)_2_.^21,37,38^ In this case, a central glutamic acid (Glu) linker connects the two FAPi units to the chelator. The replacement of the previously used SA linker with glutamic acid was a deliberate design strategy to increase hydrophilicity, thereby promoting faster clearance from nontarget tissues and improving overall pharmacokinetic performance. [^177^Lu]­Lu-DOTAGA.Glu.(FAPi)_2_ showed higher hydrophilicity, superior *in vitro* affinity and selectivity as well as improved pharmacokinetics.^21,38^ These advances led to a reduced radiation dose to critical healthy organs like liver and colon in both preclinical and clinical studies compared to [^177^Lu]­Lu-DOTAGA.(SA.FAPi)_2_.^21^

The linker and spacer are supposed to strongly impact the *in vivo* behavior of FAPi homodimers in a way that those structural modifications toward third-generation FAPi homodimers may provide a versatile platform to even further improve tumor targeting, imaging contrast, and, most importantly, therapeutic efficacy.^21,24,25,37^

Accordingly, in this study we describe the design, synthesis, and evaluation of a series of third-generation FAPi homodimers incorporating diverse spacer and linker architectures. The new third-generation compounds are DOTAGA.NPyr.(FAPi)_2_, DO3A.NPyr.(FAPi)_2_, DOTAGA.(Glu)_2_.(FAPi)_2_, DO3A.(Glu)_2_.(FAPi)_2_, DOTAGA.PEG_2_.Glu.(FAPi)_2_ (Figure 1). The DOTAGA- and DO3A.NPyr.(FAPi)_2_ derivatives use 3-aminopyrrolidine (NPyr) as the central linker, whereas the remaining compounds incorporate a glutamic acid (Glu) unit. This design choice introduces a nitrogen atom for the tris-linkage in the NPyr-based constructs, in contrast to the carbon-based linkage formed with Glu. In the DOTAGA- and DO3A.(Glu)_2_.(FAPi)_2_ pair, Glu serves as the central linker, with an additional Glu residue functioning as a spacer. This spacer increases the distance between the central linker and the chelator. To further enhance hydrophilicity, a polar spacer bearing a free carboxylic acid was introduced. Moreover, incorporating a diethylene glycol (PEG_2_) spacer led to the development of DOTAGA.PEG_2_.Glu.(FAPi)_2_.

**Figure 1:**
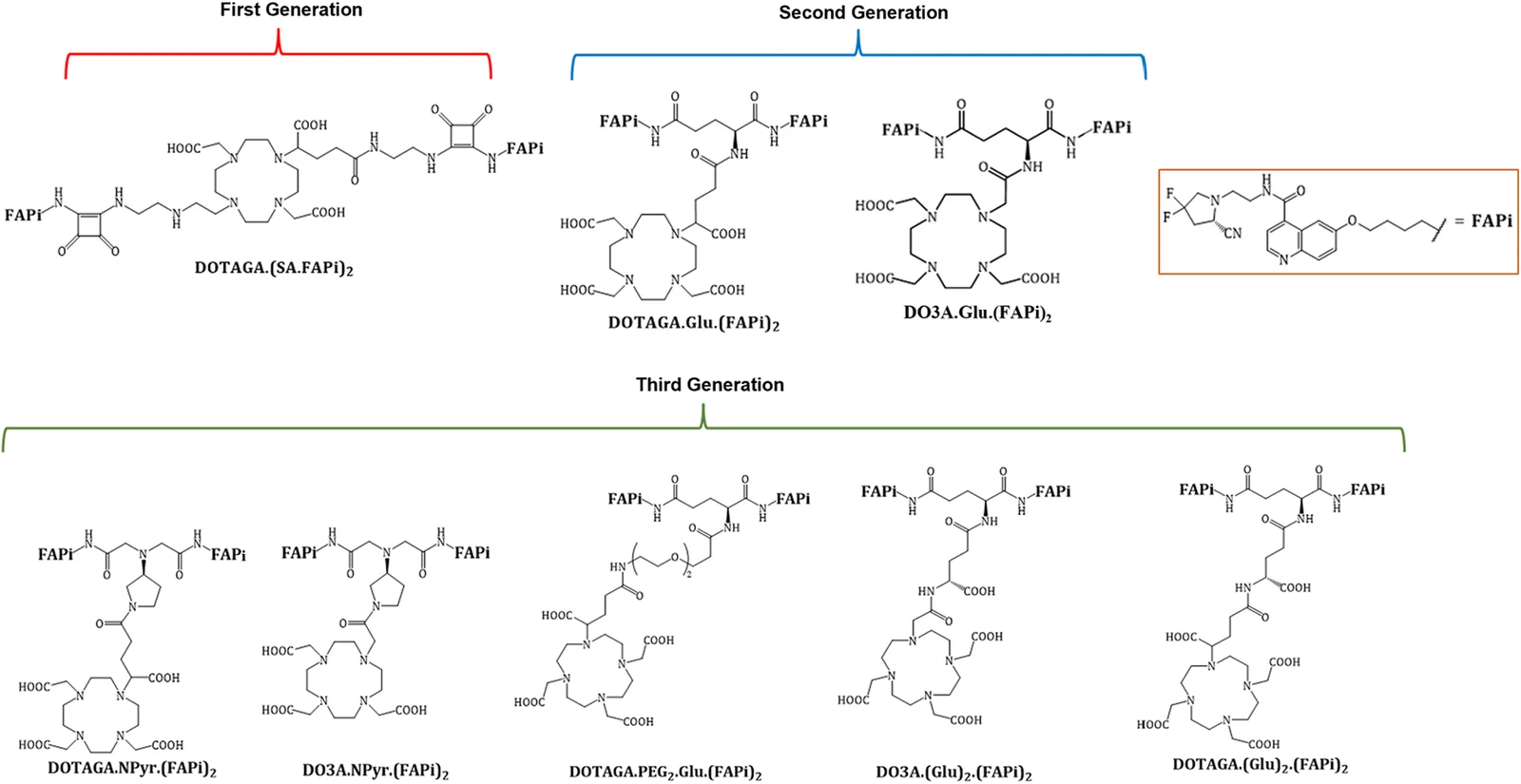
Structures of first-generation, second-generation and third-generation FAPi homodimers.

The aim was to investigate whether modifying the central linker or introducing an additional spacer, compared with the reference compound DOTAGA.Glu.(FAPi)_2_, could potentially increase (i) the radiolabeling efficiency and *in vitro* characteristics, lead to enhanced (ii) *in vitro* binding profiles and (iii) ultimately improve *in vivo* pharmacological performance.

The impact of the spacer/linker units were evaluated by radiolabeling of the third-generation FAPi-homodimers with gallium-68 and lutetium-177, and further assessing the stability and lipophilicity of the generated radioligands. The potential of different linkers to reduce chelator-FAP interactions upon inhibitor binding was also examined. *In vitro* FAP affinity and selectivity over related proteases were determined *via* IC_50_ measurements. Based on the promising initial findings, [^68^Ga]­Ga-DOTAGA.(Glu)_2_.(FAPi)_2_ and [^68^Ga]­Ga-DOTAGA.PEG_2_.Glu.(FAPi)_2_ were further evaluated for their binding affinity *via* radioligand saturation studies, internalization, and metabolic stability. Their *in vivo* pharmacokinetic profiles were further elucidated by small-animal PET imaging in PC3 tumor-bearing mice, complemented by a PET-based dose-escalation study to examine the influence of the injected mass on tumor targeting, biodistribution, and clearance. These studies define a robust preclinical framework for linker-optimized FAPi homodimers and establish a solid foundation for subsequent investigations focusing on therapeutic efficacy and comprehensive dosimetry, which will be addressed in dedicated follow-up studies.

## Results

### Organic Synthesis

The third-generation FAPi homodimers were successfully synthesized using a strategy analogous to that established for second-generation compounds, with targeted modifications at the linker site to investigate the impact of spacer architecture on compound performance.^21,38^

#### Synthesis of DOTAGA.NPyr.(FAPi)_2_ and DO3A.NPyr.(FAPi)_2_

DOTAGA.NPyr.(FAPi)_2_ **8** and DO3A.NPyr.(FAPi)_2_ **11** were synthesized from (*S*)-1-Boc-3-aminopyrrolidine **1**, following the synthetic route indicated in Figure 2.

**Figure 2:**
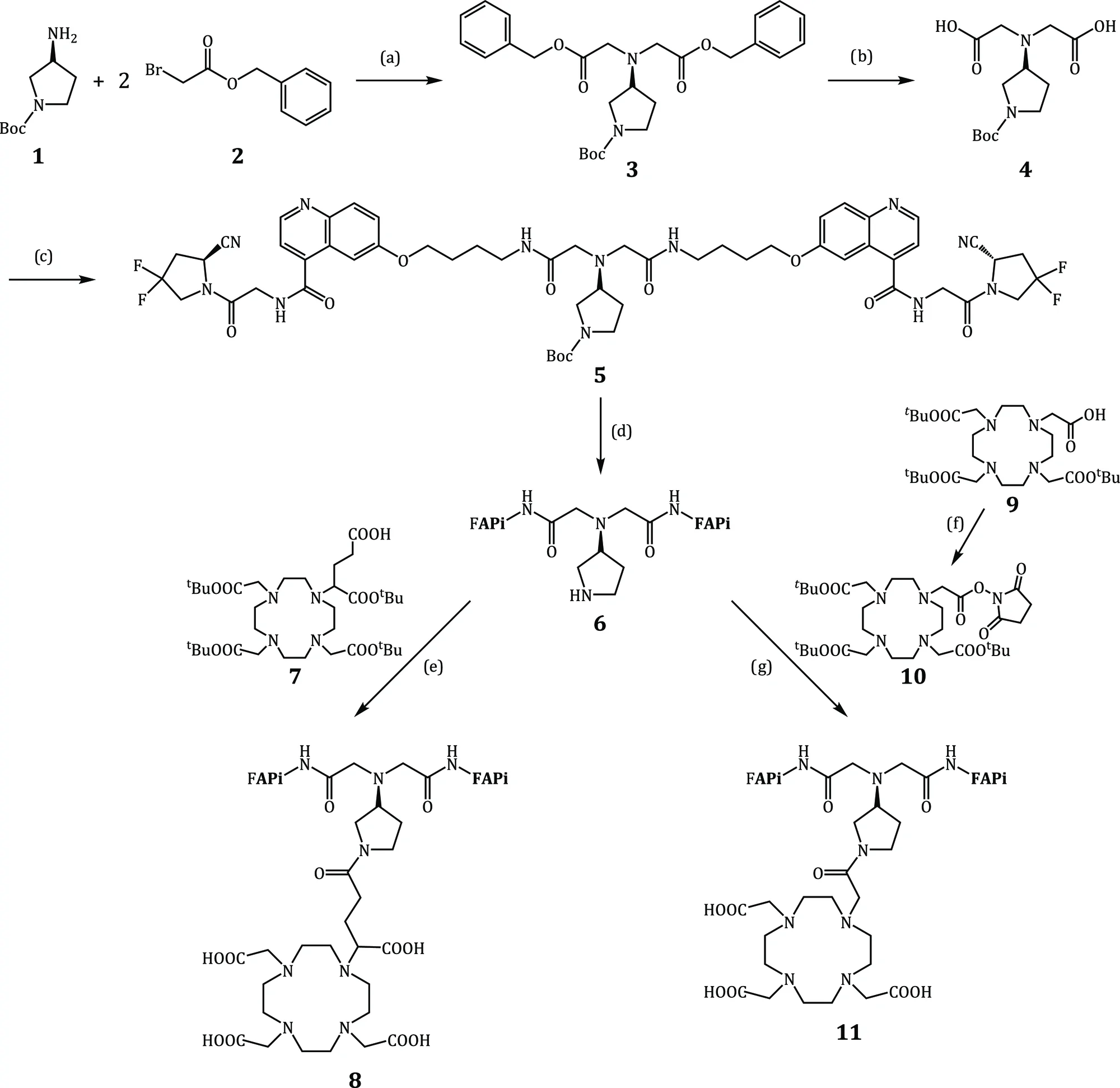
Synthesis of DOTAGA.NPyr.(FAPi)_2_ **8** and DO3A.NPyr.(FAPi)_2_ **11**: (a) DIPEA, MeCN, RT, 2 h, 47%; (b) H_2_, Pd/C, MeOH, RT, 2 d, 85%; (c) 2 FAPi-NH_2_*TFA, HOBt, EDC*HCl, DIPEA, DMF, 30 °C, 3 d, 90%; (d) TFA/TIPS/H_2_O (95:2.5:2.5), RT, 1 h, 100%; (e) 1. NHS, HBTU, DMF, 30 °C, 1 d; 2. DIPEA, DMF, 40 °C, 2 d; 3. TFA/MeCN/TIPS/H_2_O (100:10:5:2.5), RT, 5 h, 11% (over 3 steps); (f) NHS, HBTU, MeCN, DMF, 30 °C, 1 d, 96%; (g) 1. DIPEA, DMF, 40 °C, 3 d; 2. TFA/TIPS/H_2_O (94:3:3), RT, 12 h, 10% (over 2 steps).

Boc-NPyr­(OBzl)_2_ **3** was synthesized with a yield of 47%. The synthesis was designed to obtain the monosubstituted derivative Boc-NPyr­(OBzl) with a yield of 35% using 1.32 equiv of benzyl bromoacetate **2** while compound **3** was isolated as a side-product. The benzyl ester groups were removed under reductive Pd/C-catalyzed hydrogenation conditions to give Boc-NPyr **4**. Consistent with our previous procedures,^21^ **4** was coupled twice with FAPi-NH_2_·TFA using 2.75 equiv using HOBt, EDC*HCl, and DIPEA in dry DMF. After removing the Boc-protecting group with TFA/TIPS/H_2_O (95:2.5:2.5), the intermediate NPyr.(FAPi)_2_ **6** was obtained. The NHS ester of the chelators DOTAGA­(*^t^* Bu)_4_ **7** and DOTA-tris­(*tert*-butyl ester) **9** were synthesized on a large scale and isolated (DOTA­(*^t^* Bu)_3_-NHS **10**), and generated *in situ* (DOTAGA­(*^t^* Bu)_4_-NHS), respectively. These were subsequently coupled to the amine intermediate **6** and finally deprotected using TFA to yield DOTAGA.NPyr.(FAPi)_2_ **8** (11% over 3 steps) and DO3A.NPyr.(FAPi)_2_ **11** (10% over 2 steps), respectively. The overall yield for both compounds was 4% over all 5–6 steps.

#### Synthesis of DOTAGA.(Glu)_2_.(FAPi)_2_ and DO3A.(Glu)_2_.(FAPi)_2_

The two glutamate derivatives dibenzyl glutamate **12** and Fmoc-Glu-O*^t^* Bu **13** were used as starting materials for the synthesis of DOTAGA.(Glu)_2_.(FAPi)_2_ **18** and DO3A.(Glu)_2_.(FAPi)_2_ **19** (Figure 3), using HBTU and HOBt as coupling agents and resulting in a high yield of 95%. Reductive cleavage of both the benzyl ester groups gave Fmoc-Glu­(O*^t^* Bu).Glu **15** in nearly quantitative yield, followed by double coupling of FAPi-NH_2_ using previously described conditions (3.0 equiv amine), based on the method of Martin et al. with 79% yield.^21^ The Fmoc protecting group was cleaved with 10% (v/v) piperidine in DMF yielding the FAPi dimer Glu­(O*^t^* Bu).Glu.(FAPi)_2_ **17** quantitatively.

**Figure 3:**
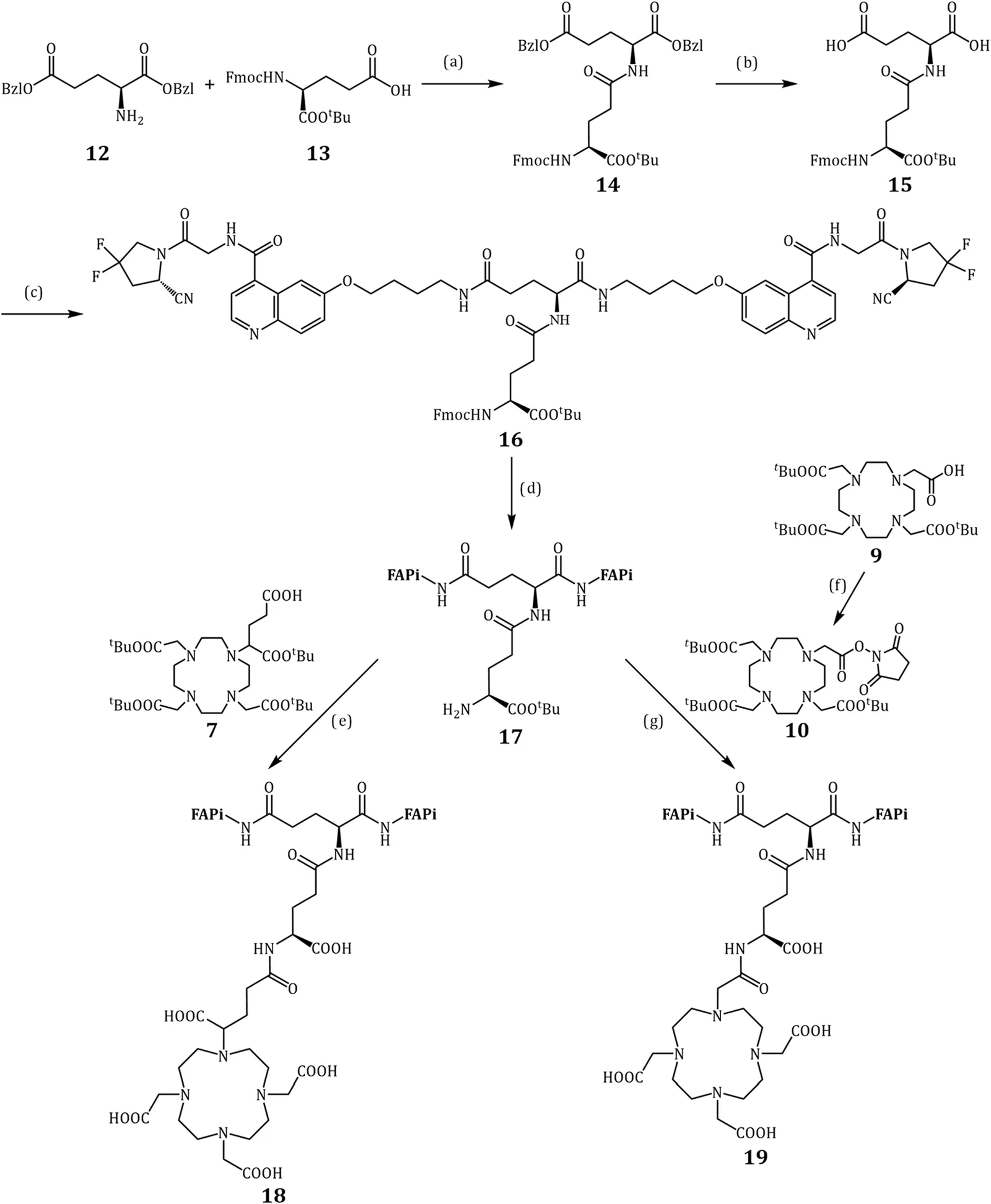
Synthesis of DOTAGA.(Glu)_2_.(FAPi)_2_ **18** and DO3A.(Glu)_2_.(FAPi)_2_ **19**: (a) HBTU, HOBt, DIPEA, DMF, RT, 5 d, 95%; (b) H_2_, Pd/C, THF, RT, 1 d, 99%; (c) 2 FAPi-NH_2_*TFA, HOBt, EDC*HCl, DIPEA, DMF, RT, 5 d, 79%; (d) Piperidine (10% v/v), DMF, RT, 90 min, 100%; (e) 1. HATU, DIPEA, DMF, RT, 1 h; 2. DIPEA, DMF, RT, 1 d; 3. TFA/MeCN/TIPS/H_2_O (90:5:2.5:2.5), RT, 6 h, 16% (over 3 steps); (f) NHS, HBTU, MeCN, DMF, 30 °C, 1 d, 96%; (g) 1. DIPEA, DMF, 40 °C, 3 d; 2. TFA/MeCN/TIPS/H_2_O (90:5:2.5:2.5), RT, 5 h, 10% (over 2 steps).

Finally, deprotection of the carboxylic acids and subsequent RP-HPLC purification gave DOTAGA.(Glu)_2_.(FAPi)_2_ **18** and DO3A.(Glu)_2_.(FAPi)_2_ **19** with isolated yields of 16% and 10%, and overall yields of 12% and 7%, respectively.

#### Synthesis of DOTAGA.PEG_2_.Glu.(FAPi)_2_

Synthesis of DOTAGA.PEG_2_.Glu.(FAPi)_2_ is depicted in Figure 4 and was initiated by coupling dibenzyl glutamate **12** and Fmoc-N-amido-dPEG2 acid **20** using HBTU and HOBt to produce Fmoc-PEG_2_.Glu­(OBzl)_2_ **21** in nearly quantitative yield (99%).

**Figure 4:**
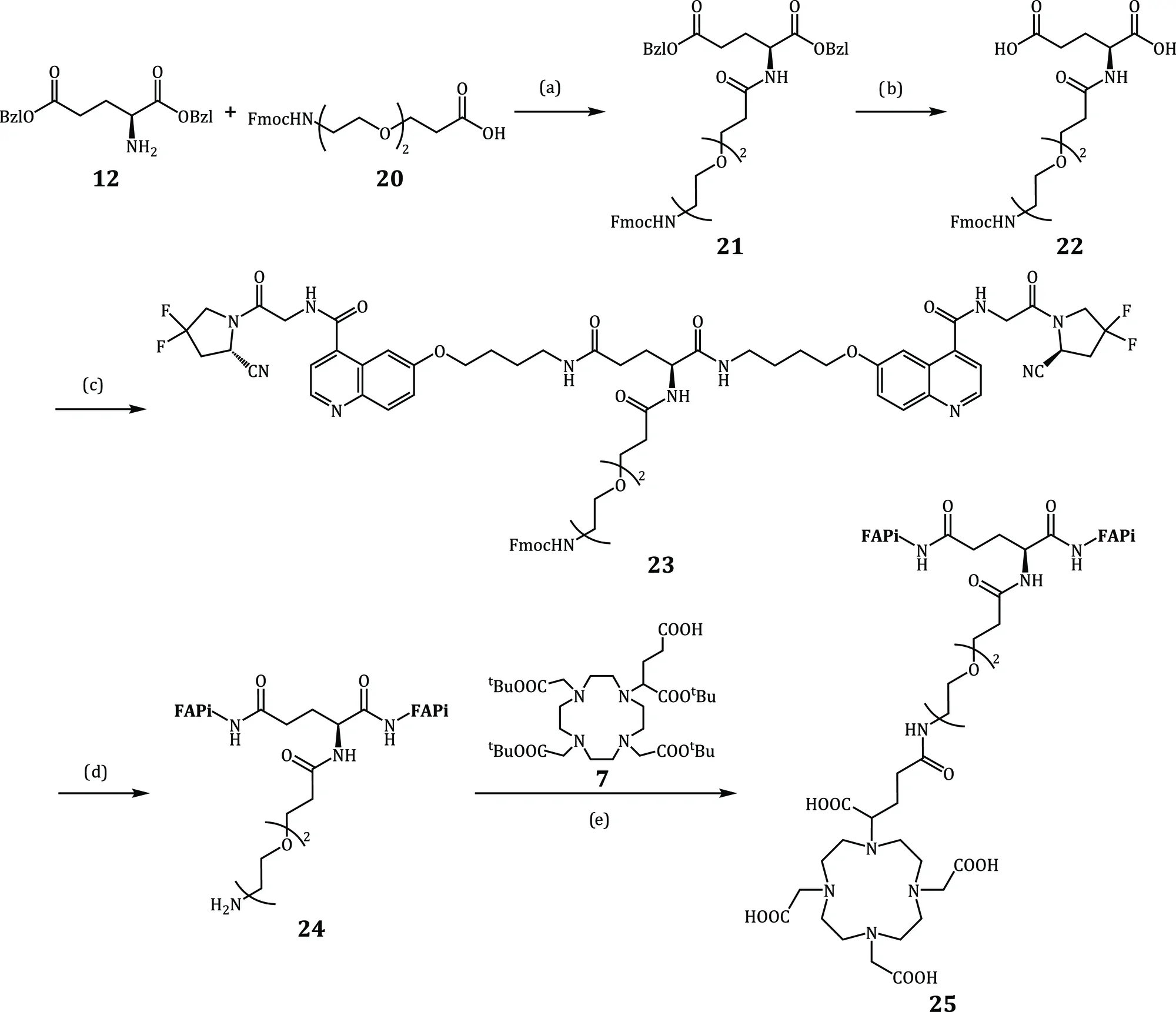
Synthesis of DOTAGA.PEG_2_.(FAPi)_2_ **25**: (a) HBTU, HOBt, DIPEA, DMF, RT, 1 d, 99%; (b) H_2_, Pd/C, THF, RT, 1 d, 84%; (c) 2 FAPi-NH_2_*TFA, HOBt, EDC*HCl, DIPEA, DMF, RT, 1 d, 97%; (d) Piperidine (10% v/v), DMF, RT, 2 h, 100%; (e) 1. HATU, DIPEA, DMF, 30 °C, 45 min; 2. DIPEA, DMF, RT-30 °C, 3 d; 3. TFA/MeCN/TIPS/H_2_O (100:10:5:2.5), RT, 6 h, 21% (over 3 steps).

The benzyl protecting groups were removed by hydrogenation over palladium on carbon, yielding the free carboxylic acid **22**, which was then coupled twice with FAPi-NH_2_ using EDC*HCl, HOBt, and DIPEA in dry DMF (2.5 equiv of amine).^21^ The product Fmoc-PEG_2_.Glu.(FAPi)_2_ **23** was isolated in a high yield of 97%. The Fmoc protecting group was removed using 10% (v/v) piperidine in DMF yielded the FAPi dimer PEG_2_.Glu.(FAPi)_2_ **24** quantitatively and coupled with DOTAGA­(*^t^* Bu)_4_ **7** using HATU. The final deprotection was performed with TFA/MeCN/TIPS/H_2_O (100:10:5:2.5), followed by purification *via* RP-HPLC to yield the final product DOTAGA.PEG_2_.Glu.(FAPi)_2_ **25** in 21% over 3 steps. The overall yield was 17% over 5 steps.

### Radiolabeling of the FAPi Dimers with Gallium-68

The radiolabeling efficiency and reaction kinetics of the five synthesized FAPi dimers with gallium-68 were investigated under different buffer conditions and precursor concentrations. The radioligands tested included [^68^Ga]­Ga-DOTAGA.NPyr.(FAPi)_2_ ([^68^Ga]­Ga**-8**), [^68^Ga]­Ga-DO3A.NPyr.(FAPi)_2_ ([^68^Ga]­Ga-**11**), [^68^Ga]­Ga-DOTAGA.(Glu)_2_.(FAPi)_2_ ([^68^Ga]­Ga-**18**), [^68^Ga]­Ga-DO3A.(Glu)_2_.(FAPi)_2_ ([^68^Ga]­Ga-**19**), and [^68^Ga]­Ga-DOTAGA.PEG_2_.Glu.(FAPi)_2_ ([^68^Ga]­Ga**-25**). For each compound, both the reaction kinetics and radiolabeling yields were analyzed under varying conditions. The temperature of the radiolabeling reaction was adjusted to 95 °C and 50 MBq of gallium-68 activity was used to determine the optimal labeling conditions.

#### Buffer System and Labeling Efficiency

The radiolabelings were tested using three buffer systems: 1 M HEPES (pH 4.5–5.5), 1 M ammonium acetate (AmOAc) pH 4.0–4.5, and 1 M sodium acetate (NaOAc) pH 4.5 (Figure 5). For most of the compounds, HEPES buffer was proved to be superior. Specifically, for [^68^Ga]­Ga-**8** and [^68^Ga]­Ga-**18**, the radiolabeling was only successful in HEPES, while AmOAc and NaOAc did not result to a radiolabeled product. In contrast, [^68^Ga]­Ga-**25** exhibited consistent high radiochemical conversion (RCC) around 95% across all three buffer systems, showing superior behavior compared to the others.

**Figure 5:**
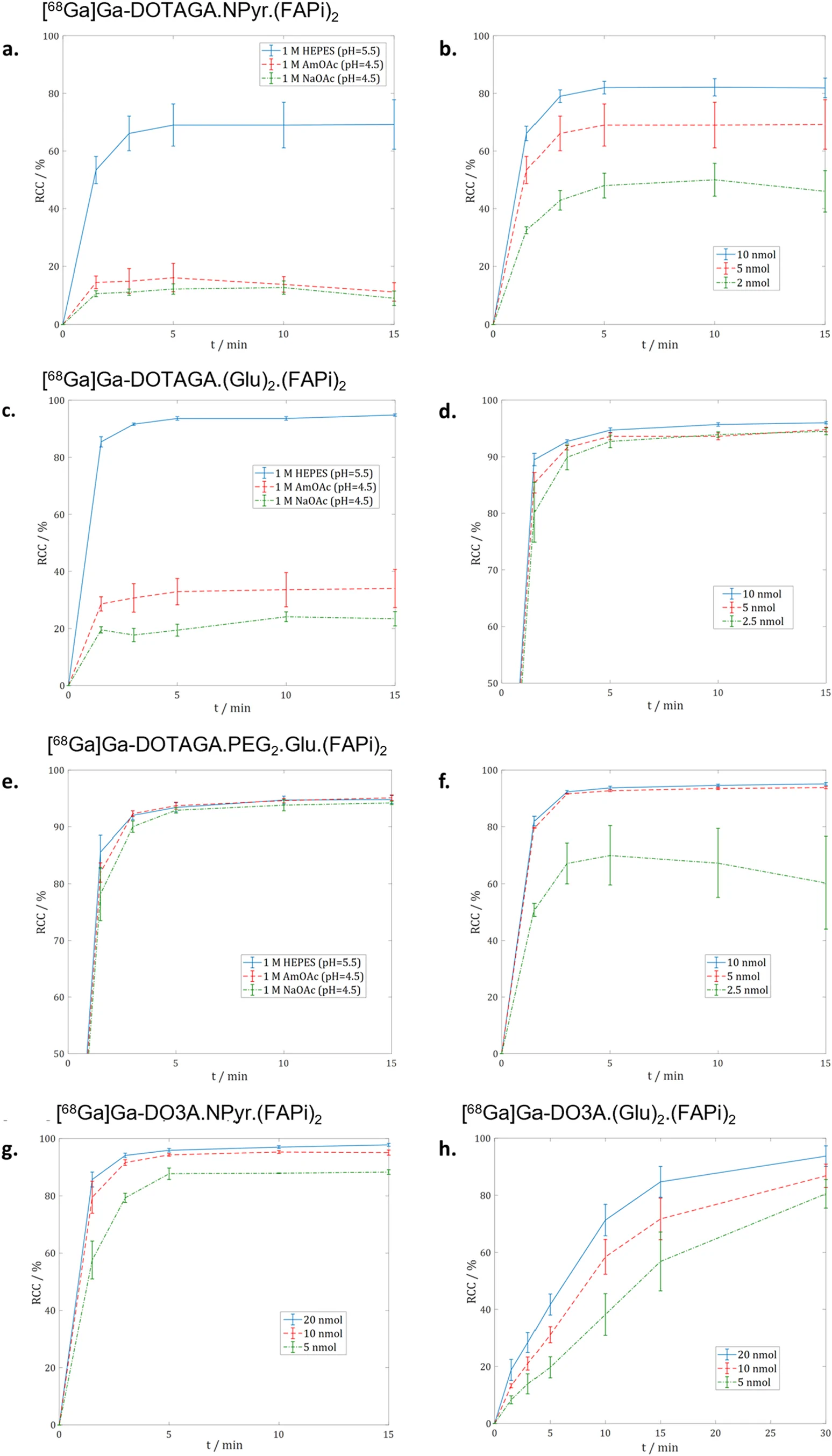
Reaction kinetics (radiochemical conversion (RCC) in %) of [^68^Ga]­Ga-DOTAGA.NPyr.(FAPi)_2_ [^68^Ga]­Ga**-8** (a, b), [^68^Ga]­Ga-DOTAGA.(Glu)_2_.(FAPi)_2_ [^68^Ga]­Ga**-18** (c, d), [^68^Ga]­Ga-DOTAGA.PEG_2_.Glu.(FAPi)_2_ [^68^Ga]­Ga**-25** (e, f), [^68^Ga]­Ga-DO3A.NPyr.(FAPi)_2_ [^68^Ga]­Ga**-11** (g), [^68^Ga]­Ga-DO3A.(Glu)_2_.(FAPi)_2_ [^68^Ga]­Ga**-19** (h) and at 95 °C with 50 MBq of gallium-68, 400 μL buffer solution (1 M) HEPES (pH = 5.5), 1 M AmOAc (pH = 4.5) or 1 M NaOAc (pH = 4.5) and precursor amounts (2, 5, 10 and 20 nmol) in 1 M HEPES (pH = 5.5).

#### Precursor Concentration and Labeling Efficiency

The effect of the concentration of the tested precursor (2–20 nmol) was systematically studied (Table 1 and Figure 5):

- [^68^Ga]­Ga-**8**: Radiolabeling in HEPES yielded in RCCs up to 82.0 ± 2.2% when 10 nmol of the precursor were used. The lower amounts of precursors resulted in lower RCCs. No significant increase was observed beyond 5 min of incubation.
- **[** ^**68**^ **Ga]­Ga-11**: High RCCs were achieved in HEPES after 10 min of incubation; 97.0 ± 0.5% (20 nmol), 95.3 ± 0.5% (10 nmol) and 87.9 ± 0.1% (5 nmol).
- **[** ^**68**^ **Ga]­Ga-18**: Efficient labeling in HEPES resulted in RCCs of 94.5 ± 0.6% (2 nmol), 94.8 ± 0.4% (5 nmol), and 96.0 ± 0.2% (10 nmol).
- **[** ^**68**^ **Ga]­Ga-19**: Labeling kinetics were slower, requiring up to 30 min of incubation. RCCs exceeded 80% with 5 or 10 nmol, reaching 93.7 ± 3.6% when 20 nmol of the precursor were used. RCP improved from 95.2% (HPLC) to 98.3% postpurification.
- **[** ^**68**^ **Ga]­Ga-25**: This radioligand showed consistently high RCCs (∼95%) across all buffer systems with 10 nmol precursor. Even 5 nmol resulted in RCC > 90% (93.5 ± 0.4% after 10 min) in AmOAc. However, 2 nmol was insufficient.

**Table 1:** Radiochemical Conversion (RCC) Rates (%) Using Varying Amounts of Tested Precursors (2, 5, 10, and 20 nmol) with a Fixed Gallium-68 Activity of 50 MBq^a^

	2 nmol	5 nmol	10 nmol	20 nmol	observations
[^68^Ga]Ga-**8**	RCCs ∼ 40% after 5 min	RCCs ∼ 60% after 5 min	RCCs > 80% after 5 min		No significant increase in RCCs beyond 5 min
[^68^Ga]Ga-**11**		RCCs > 85% after 5 min	RCCs > 95% after 5 min	RCCs > 95% after 5 min	Fast kinetics
[^68^Ga]Ga-**18**	RCCs > 90% after 5 min	RCCs > 90% after 5 min	RCCs > 95% after 5 min		Fast kinetics
[^68^Ga]Ga-**19**		RCCs > 80% after 30 min	RCCs > 80% after 30 min	RCCs > 90% after 30	Slow kinetics
[^68^Ga]Ga-**25**	RCCs ∼ 70% after 10 min	RCCs > 90% after 5 min	RCCs > 95% after 5 min		RCCs (∼95%) across all buffer systems.

a Manual purification using a C18 cartridge (conditioning and elution solvents are given in the materials and methods) led to a radiochemical purity (RCP) of more than 97% for all the tested radioligands (Figures S6–S15).

#### Radiolabeling of DOTAGA.(Glu)_2_.(FAPi)_2_ and DOTAGA.PEG_2_.Glu.(FAPi)_2_ with Gallium-68 for Cell and Animal Studies

DOTAGA.(Glu)_2_.(FAPi)_2_ and DOTAGA.PEG_2_.Glu.(FAPi)_2_ were successfully labeled with gallium-68 using the Modular-Lab PharmTracer module in >98% radiochemical purity, for cells and animal studies. Their apparent molar activity (A_m_) was in the range of 8 to 18 GBq/μmol (nondecay corrected). The results of the quality control (radio- HPLC and TLC) of both radioligands are given in the Supporting Data (Figure S16).

### Radiolabeling of the FAPi Dimers with Lutetium-177

HEPES buffer was shown to be superior in labeling with gallium-68. Therefore, the radiolabeling efficiency and reaction kinetics of the FAPi dimers with lutetium-177 was investigated at different precursor concentrations (2, 5, and 10 nmol) in 1 M HEPES buffer (Table 2 and Figure 6). The tested compounds included [^177^Lu]­Lu-DOTAGA.NPyr.(FAPi)_2_ ([^177^Lu]­Lu-**8**), [^177^Lu]­Lu-DO3A.NPyr.(FAPi)_2_ ([^177^Lu]­Lu-**11**), [^177^Lu]­Lu-DOTAGA.(Glu)_2_.(FAPi)_2_ ([^177^Lu]­Lu-**18**), and [^177^Lu]­Lu-DOTAGA.PEG_2_.Glu.(FAPi)_2_ ([^177^Lu]­Lu-**25**). For each generated radioligand, both the reaction kinetics and the radiolabeling yields were analyzed under different precursor concentrations. The temperature of the radiolabeling reaction was adjusted to 90 °C and 50 MBq of lutetium-177 activity was used to determine the optimal labeling conditions. All the radioligands were produced with a radiochemical purity (RCP) of about 99% and no further purification step was necessary for further studies (Figures S17–S20).

**Figure 6:**
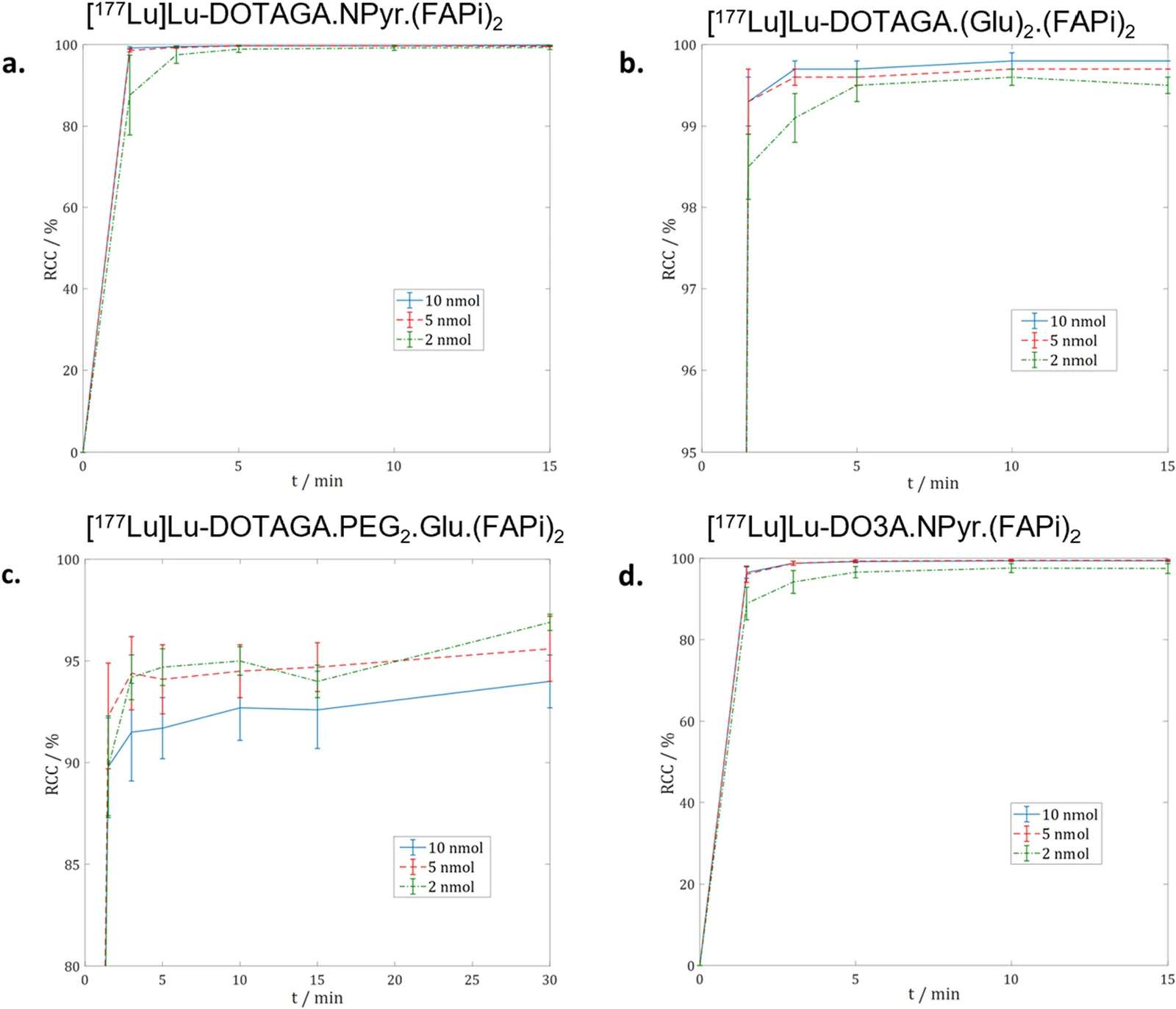
Reaction kinetics (radiochemical conversion (RCC) in %) of [^177^Lu]­Lu-DOTAGA.NPyr.(FAPi)_2_ ([^177^Lu]­Lu**-8**) (a), [^177^Lu]­Lu-DOTAGA.(Glu)_2_.(FAPi)_2_ ([^177^Lu]­Lu**-18**) (b), [^177^Lu]­Lu-DOTAGA.PEG_2_.Glu.(FAPi)_2_ ([^177^Lu]­Lu**-25**) (c) and [^177^Lu]­Lu-DO3A.NPyr.(FAPi)_2_ ([^177^Lu]­Lu**-11**) (d) at 95 °C with 50 MBq of lutetium-177 and precursor concentrations (2, 5, and 10 nmol) in 400 μL 1 M HEPES (pH = 5.5) buffer.

**Table 2:** Radiochemical Conversion (RCC) Rates (%) Using Varying Amounts of Tested Precursors (2, 5, and 10 nmol) with a Fixed Lutetium-177 Activity of 50 MBq

	2 nmol	5 nmol	10 nmol	RCP
[^177^Lu]Lu-**8**	RCCs > 99% after 10 min	RCCs > 99% after 2–3 min	RCCs > 99% after 2–3 min	>99% without any purification
[^177^Lu]Lu-**11**	RCCs > 99% after 10 min	RCCs > 99% after 4–5 min	RCCs > 99% after 4–5 min	>99% without any purification
[^177^Lu]Lu-**18**	RCCs > 99% after 3–4 min	RCCs > 99% after 1–2 min	RCCs > 99% after 1–2 min	>99% without any purification
[^177^Lu]Lu-**25**	RCCs > 90% after 2–3 min	RCCs > 90% after 2–3 min	RCCs > 90% after 2–3 min	>99% without any purification

#### Precursor Concentration and Labeling Efficiency

The effect of precursor concentration (10, 5, and 2 nmol) was systematically studied (Table 1 and Figure 6):

- [^177^Lu]­Lu-**8:** RCCs > 99% reached after 2–3 min (10 and 5 nmol) and 10 min (2 nmol) of heating. RCP > 99% without any purification.
- [^177^Lu]­Lu-**11:** RCCs > 99% was observed with 10 and 5 nmol after 4–5 min. High yields were also observed with only 2 nmol after 10 min. Quantitative RCP without any purification
- [^177^Lu]­Lu-1**8:** RCC > 99% was obtained after 1–2 min when 10 and 5 nmol were used and after 3–4 min with 2 nmol. RCP > 99% without any purification
- [^177^Lu]­Lu-**25:** RCC > 99% was obtained after 2–3 min (with 10, 5, and 2 nmol). RCP > 99% without any purification

### Stability Studies in Human Serum (HS) and Phosphate-Buffered Saline (PBS)

The radioligand stability was determined in human serum (HS) and phosphate-buffered saline (PBS) (n = 3) by incubating the ^68^Ga-labeled tracers ([^68^Ga]­Ga**-8**, -**11**, -**18**, -**19** and -**25**) (10 MBq, 1 nmol) in 0.5 mL of HS and PBS at 37 °C for 120 min (Figure S21) and analyzed *via* radio-TLC as described in Martin et al.^21^ All radioligands were stable over two half-lives, with stability of >95% in HS and PBS after 120 min except [^68^Ga]­Ga**-19** that showed stability <90% in PBS and <80% in HS.

The stability of ^177^Lu-labeled radioligands [^177^Lu]­Lu**-8**, -**11**, -**18**, and -**25** was investigated in a similar way to their ^68^Ga-labeled analogues. The incubation in HS and PBS at 37 °C was carried out for 14 days. All radioligands showed no degradation and were stable over 2 half-lives (14 days), with stability/RCP > 99% in HS and PBS (Figure S22).

### Lipophilicity/Protein Binding Studies

The lipophilicity of the radioligands was determined in PBS and 1-octanol (*n* = 4), and their respective Log *D*_octanol/PBS_ values are presented in Table 3. For comparison in Table 3, we also have provided the Log *D*_octanol/PBS_ values of the three generations of FAPi ligands reported previously by our group.^10,17,37^

**Table 3:** Log *D*_7.4_ Values of Different Labeled FAPi-Based Radiopharmaceuticals

complex	Log *D*_octanol/PBS_	references
[^68^Ga]Ga-DOTA.SA.FAPi	–3.38 ± 0.03	Läpchen et al.^17^
[^177^Lu]Lu-DOTA.SA.FAPi	–2.86 ± 0.06	Läpchen et al.^17^
[^68^Ga]Ga-DOTAGA.(SA.FAPi)_2_	–1.83 ± 0.02	Läpchen et al.^17^
[^177^Lu]Lu-DOTAGA.(SA.FAPi)_2_	–1.71 ± 0.03	Läpchen et al.^17^
[^68^Ga]Ga-DOTAGA.Glu.(FAPi)_2_	–2.90 ± 0.10	Bilinska et al.^37^
[^177^Lu]Lu-DOTAGA.Glu.(FAPi)_2_	–3.00 ± 0.10	Bilinska et al.^37^
[^68^Ga]Ga-DO3A.Glu.(FAPi)_2_	–2.20 ± 0.04	Bilinska et al.^37^
[^177^Lu]Lu-DO3A.Glu.(FAPi)_2_	–1.70 ± 0.01	Bilinska et al.^37^
[^68^Ga]Ga-DOTAGA.NPyr.(FAPi)_2_ (^68^Ga-8)	–2.71 ± 0.13	Present study
[^177^Lu]Lu-DOTAGA.NPyr.(FAPi)_2_ (^177^Lu-8)	–3.15 ± 0.10	Present study
[^68^Ga]Ga-DO3A.NPyr.(FAPi)_2_ (^68^Ga-11)	–2.43 ± 0.10	Present study
[^177^Lu]Lu-DO3A.NPyr.(FAPi)_2_ (^177^Lu-11)	–2.31 ± 0.06	Present study
[^68^Ga]Ga-DOTAGA.(Glu)_2_.(FAPi)_2_ (^68^Ga-18)	–3.49 ± 0.25	Present study
[^177^Lu]Lu-DOTAGA.(Glu)_2_.(FAPi)_2_ (^177^Lu-18)	–3.49 ± 0.27	Present study
[^68^Ga]Ga-DO3A.(Glu)_2_.(FAPi)_2_ (^68^Ga-19)	–2.59 ± 0.13	Present study
[^68^Ga]Ga-DOTAGA.PEG_2_.(FAPi)_2_ (^68^Ga-25)	–3.04 ± 0.28	Present study
[^177^Lu]Lu-DOTAGA.PEG_2_.(FAPi)_2_ (^177^Lu-25)	–3.32 ± 0.14	Present study

All the third-generation radioligands showed high hydrophilicity in the same range or even higher compared to the first and second generation radioligands. The values vary between −2.31 and −3.49 for the different radioligands with the DO3A compounds **11** and **19** being the least and **18** being the most hydrophilic.

The percentage of the protein binding for [^68^Ga]­Ga-DOTAGA.(Glu)_2_.(FAPi)_2_ was 11.3 ± 0.4 and 8.7 ± 0.4 after 30 and 60 min of incubation. [^68^Ga]­Ga-DOTAGA.PEG_2_.Glu.(FAPi)_2_ showed slightly lower protein binding (8.4 ± 0.6 and 6.5 ± 0.3 after 30 and 60 min of incubation).

### *In Vitro*-Inhibition Assays

The precursors were tested for their *in vitro* affinity for FAP, PREP and the DPPs (DPP4, DPP8 and DPP9).^38^ The measured IC_50_ values are shown in Table 4 as well as the selectivity index (SI) which is the ratio of IC_50_ (PREP or DPP4) to IC_50_ (FAP).

**Table 4:** IC_50_ Value and Selectivity Index (SI) of Different FAPis^a^ ^21,38^

	IC_50_/nM	IC_50_/μM				SI	SI	
compound	FAP	PREP	DPP4	DPP8	DPP9	PREP/FAP	DPP4/FAP	references
DOTAGA.NPyr.(FAPi)_2_ (**8**)	0.50 ± 0.03	0.73 ± 0.16	2.68 ± 0.10	0.106 ± 0.002	0.35 ± 0.02	1500	5300	Present study
DO3A.NPyr.(FAPi)_2_ (**11**)	0.48 ± 0.04	1.86 ± 0.16	3.90 ± 0.22	0.208 ± 0.004	0.20 ± 0.01	3900	8200	Present study
DOTAGA.(Glu)_2_.(FAPi)_2_ (**18**)	0.48 ± 0.06	0.63 ± 0.05	1.37 ± 0.14	0.041 ± 0.003	0.23 ± 0.05	1300	2900	Present study
DO3A.(Glu)_2_.(FAPi)_2_ (**19)**	0.31 ± 0.18	0.41 ± 0.06	1.03 ± 0.03	0.074 ± 0.001	0.130 ± 0.005	1300	3300	Present study
DOTAGA.PEG_2_.(FAPi)_2_ (**25**)	0.37 ± 0.03	0.50 ± 0.08	2.44 ± 0.19	0.023 ± 0.007	0.059 ± 0.002	1400	6600	Present study
DOTAGA.Glu.(FAPi)_2_	0.26 ± 0.04	0.59 ± 0.10	1.19 ± 0.08	0.029 ± 0.004	0.083 ± 0.002	2269	4577	Martin et al.^21^
[^nat^Lu]Lu-DOTAGA.Glu. (FAPi)_2_	0.33 ± 0.02	0.43 ± 0.16	0.65 ± 0.04	0.22 ± 0.02	0.19 ± 0.01	1292	1967	Martin et al.^21^
DO3A.Glu.(FAPi)_2_	0.60 ± 0.04	1.00 ± 0.14	0.54 ± 0.06	1.03 ± 0.18	0.95 ± 0.11	1667	900	Martin et al.^21^
UAMC-1110	0.43 ± 0.02	1.80 ± 0.01	>10	>10	4.70 ± 0.40	4186	23,256	Martin et al.^21^

a The data for DOTAGA.Glu.(FAPi)_2_, [^nat^Lu]­Lu-DOTAGA.Glu.(FAPi)_2_, DO3A.Glu.(FAPi)_2_, UAMC-1110 are taken from Martin et al.^21,38^

The third-generation FAPi-based precursors exhibited very high affinity for FAP with subnanomolar IC_50_ (FAP) values. The selectivity for PREP and DPP4 remained at values in the μM range and, consequently, selectivity indexes (SI) > 1000 for all compounds. The respective SI of DPP8 and DPP9 *versus* FAP are between 60 and 700 and are summarized in Table S1.

### Saturation Binding/Internalization Studies

^68/nat^Ga-DOTAGA.(Glu)_2_.(FAPi)_2_ and ^68/nat^Ga-DOTAGA.PEG_2_.Glu.(FAPi)_2_, exhibited similar affinities for FAP^+^ CAFs, with *K*_d_ values of 1.1 ± 0.3 nM and 1.2 ± 0.4 nM respectively (Figure 7a,b), comparable with second generation homodimers.^37^ The *B*_max_ values were at the same level for both (0.9 ± 0.08 nM and 1.2 ± 0.12 nM respectively), which correspond to 5.4–7.2 × 10^5^ receptors per cell.

**Figure 7:**
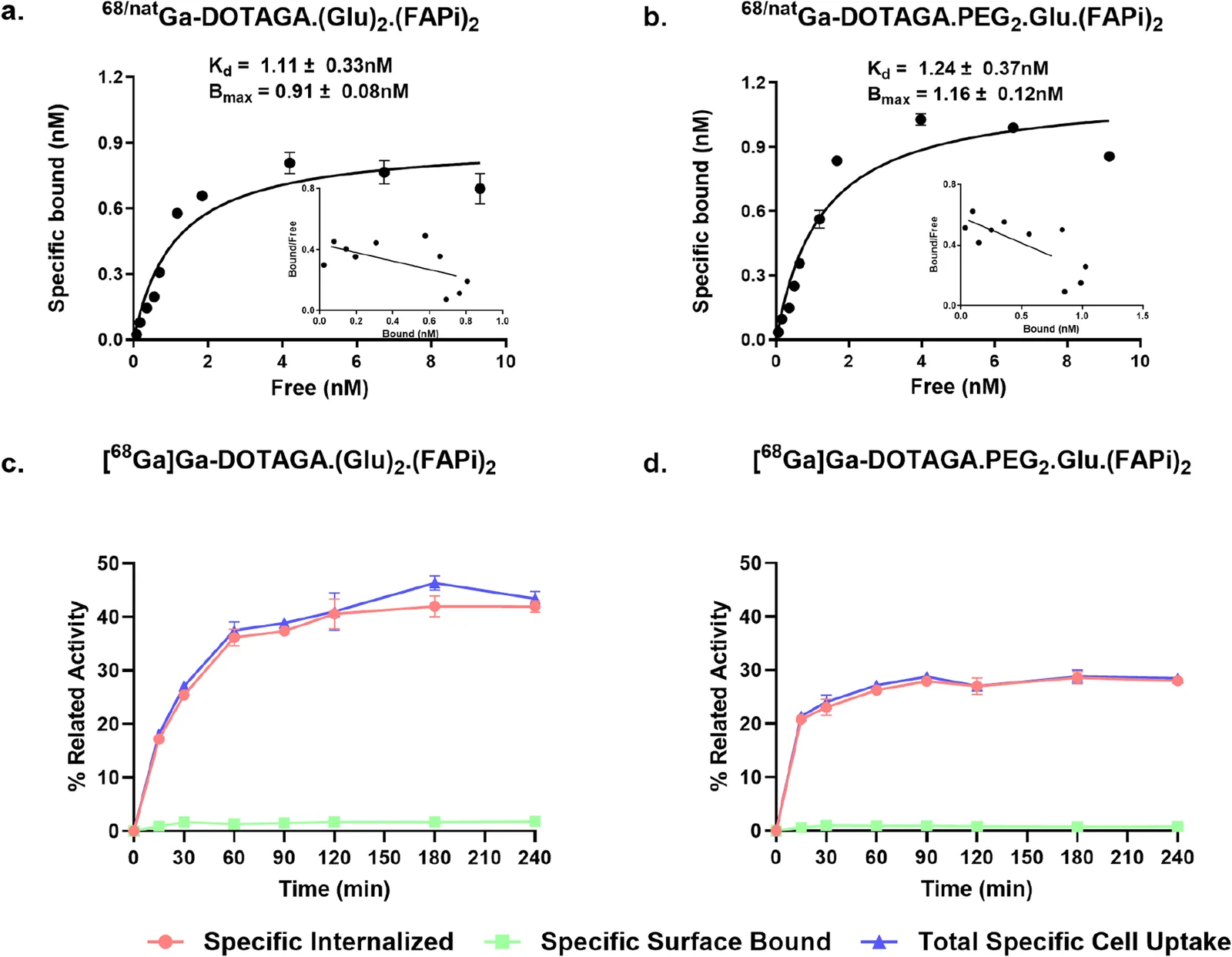
Saturation radioligands binding assays on FAP^+^ CAF cells, using increasing concentrations of (a)^68/nat^Ga-DOTAGA.(Glu)_2_.(FAPi)_2_ and (b) ^68/nat^Ga-DOTAGA.PEG_2_.Glu.(FAPi)_2_ (0.1 to 10 nM). Internalization rate and specific surface bound uptake after the incubation of CAFs with (c) [^68^Ga]­Ga-DOTAGA.(Glu)_2_.(FAPi)_2_ and (d) [^68^Ga]­Ga-DOTAGA.PEG_2_.Glu.(FAPi)_2_ at 37 °C. Total specific cell uptake calculated as specific surface bound fraction plus specific internalized fraction. Total specific cell uptake is expressed as percentage of the total applied radioactivity. Nonspecific binding was determined in the presence of 1 μM UAMC-1110. ^68/nat^Ga- DOTAGA.(Glu)_2_.(FAPi)_2_ and ^68/nat^Ga-DOTAGA.PEG_2_.Glu.(FAPi)_2_ complexes prepared *in situ*.

Both radioligands were found to be internalized rapidly by CAFs with over 30% of the total added activity being internalized already after 60 min of incubation at 37 °C (Figure 8c,d). More than 95% of the total specific cell associated activity was found to be internalized.

**Figure 8:**
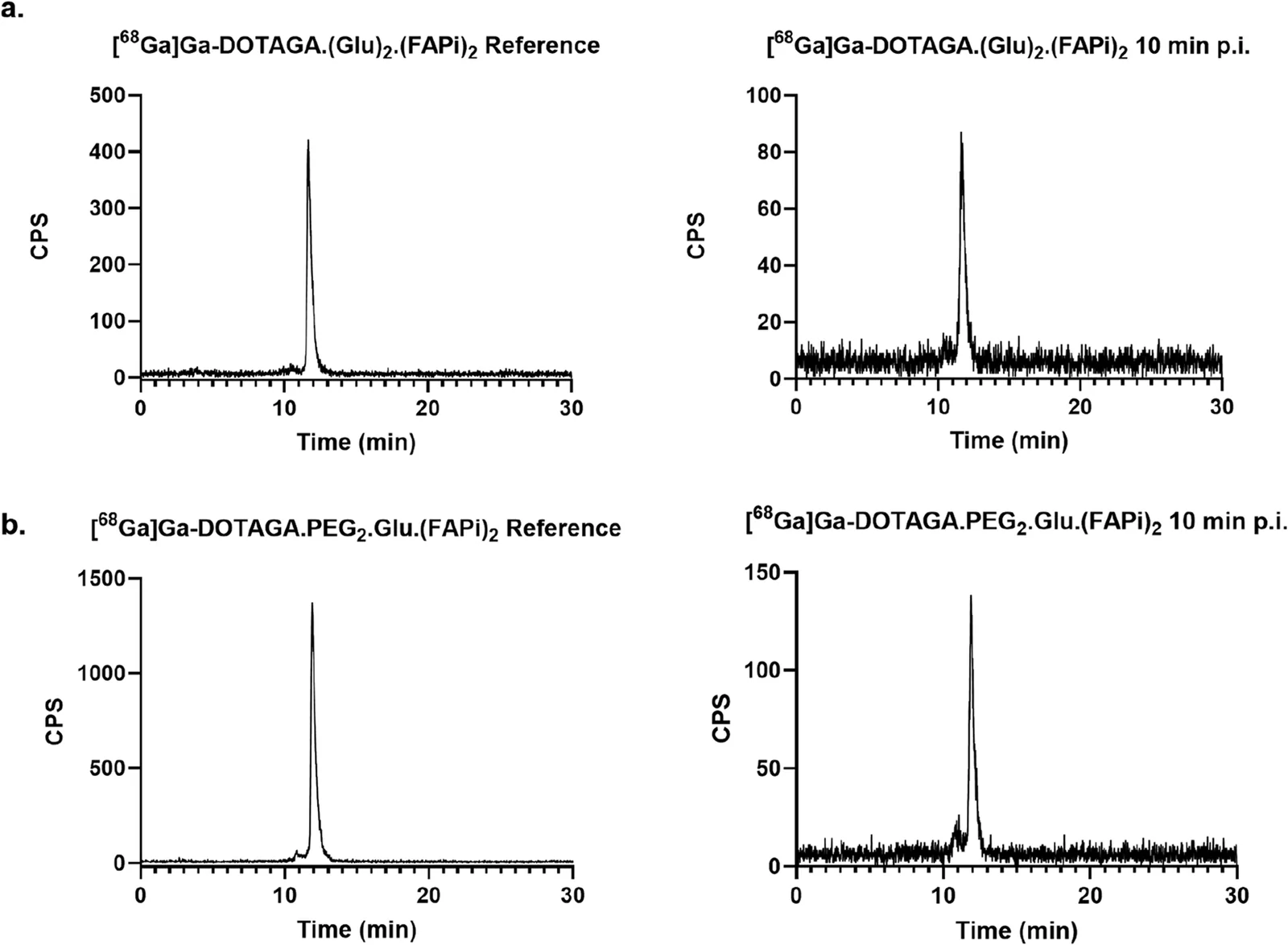
Representative radio-HPLC chromatograms of (a) [^68^Ga]­Ga-DOTAGA.(Glu)_2_.(FAPi)_2_, (b) [^68^Ga]­Ga-DOTAGA.PEG_2_.Glu.(FAPi)_2_: reference chromatograms for the radiotracers before their injection in PC3 tumor-bearing mice are presented on the left side. The right side displays the chromatograms from blood samples collected 10 min p.i. of the respective radiotracers.

Blocking studies, for all the *in vitro* studies, were conducted using an excess of UAMC-1110 (1 μM), which demonstrated negligible nonspecific binding to CAFs, indicating high specificity of the radioligands toward FAP-positive CAFs.

### Metabolic Stability

The HPLC analysis of mice plasma samples showed that the circulating radioactivity in blood 10 min (Figure 8) after injection of the radioligands contains only intact radiotracers.

### Small-Animal PET/CT Imaging

PET/CT images of PC3 xenografted mice taken 1 h after injection of [^68^Ga]­Ga-DOTAGA.(Glu)_2_.(FAPi)_2_ and [^68^Ga]­Ga-DOTAGA.PEG_2_.Glu.(FAPi)_2_ are shown in Figure 9. Each radioligand was administered at doses of 600, 1000, and 1500 pmol. Previous studies from our group demonstrated that a dose of 600 pmol of the first and second FAPi-homodimer generations achieved maximum tumor uptake. Based on these findings, we included this dose in the current study of third-generation FAPi-homodimers and added the higher doses of 1000 and 1500 pmol to assess how the increased cold mass in the formulation affects the pharmacokinetics of the radioligands.

**Figure 9:**
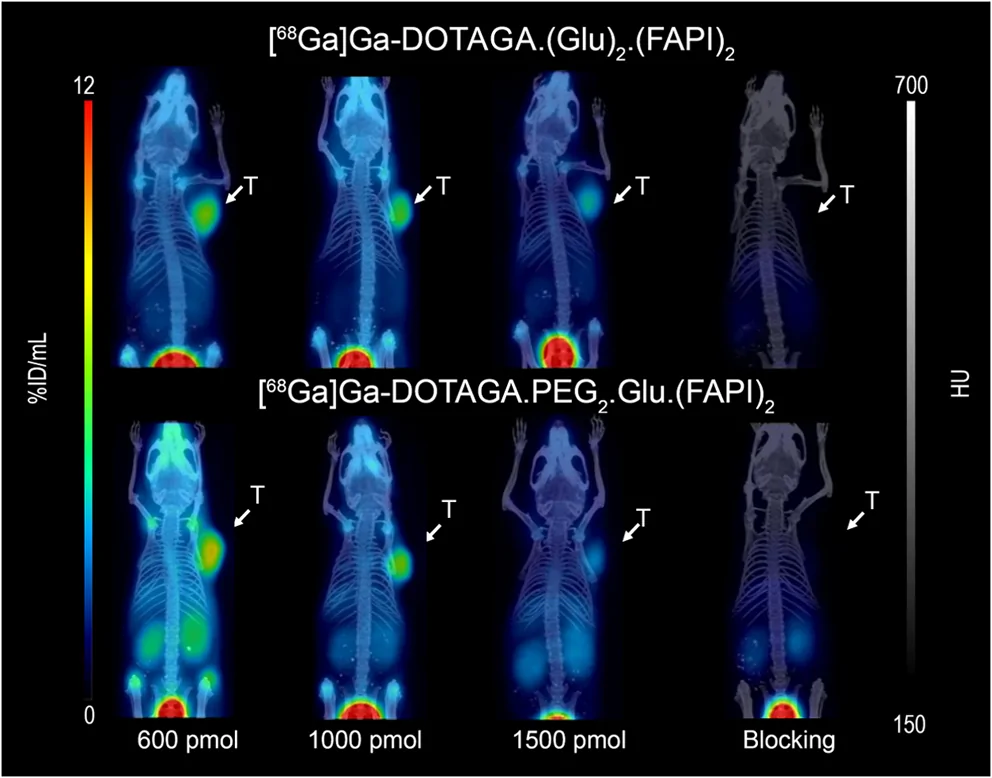
PET/CT images of mice bearing PC3 tumors were acquired 1 h after injection of 600, 1000, and 1500 pmol of [^68^Ga]­Ga-DOTAGA.(Glu)_2_.(FAPi)_2_ or [^68^Ga]­Ga-DOTAGA.PEG_2_.Glu.(FAPi)_2_. Blocking studies were also performed at 1 h postinjection.

The PET/CT images clearly showed a strong correlation between the injected mass and the *in vivo* behavior. Specifically, the 600 pmol dose led to the highest tumor uptake, as confirmed by image quantification (Figure 10a). In contrast, increasing the dose to 1000 and 1500 pmol caused a significant reduction in tumor accumulation, approximately 30% and 25%, respectively, similar for both radioligands. Additionally, uptake in nontarget organs such as bone and salivary glands (which also express FAP) decreased at higher doses. A direct comparison between the two radioligands showed that [^68^Ga]­Ga-DOTAGA.(Glu)_2_.(FAPi)_2_ had higher tumor uptake and lower background activity than [^68^Ga]­Ga-DOTAGA.PEG_2_.Glu.(FAPi)_2_, resulting in superior tumor-to-background ratios in most key organs (Figure 10b). Blocking studies confirmed the specific binding of both tracers.

**Figure 10:**
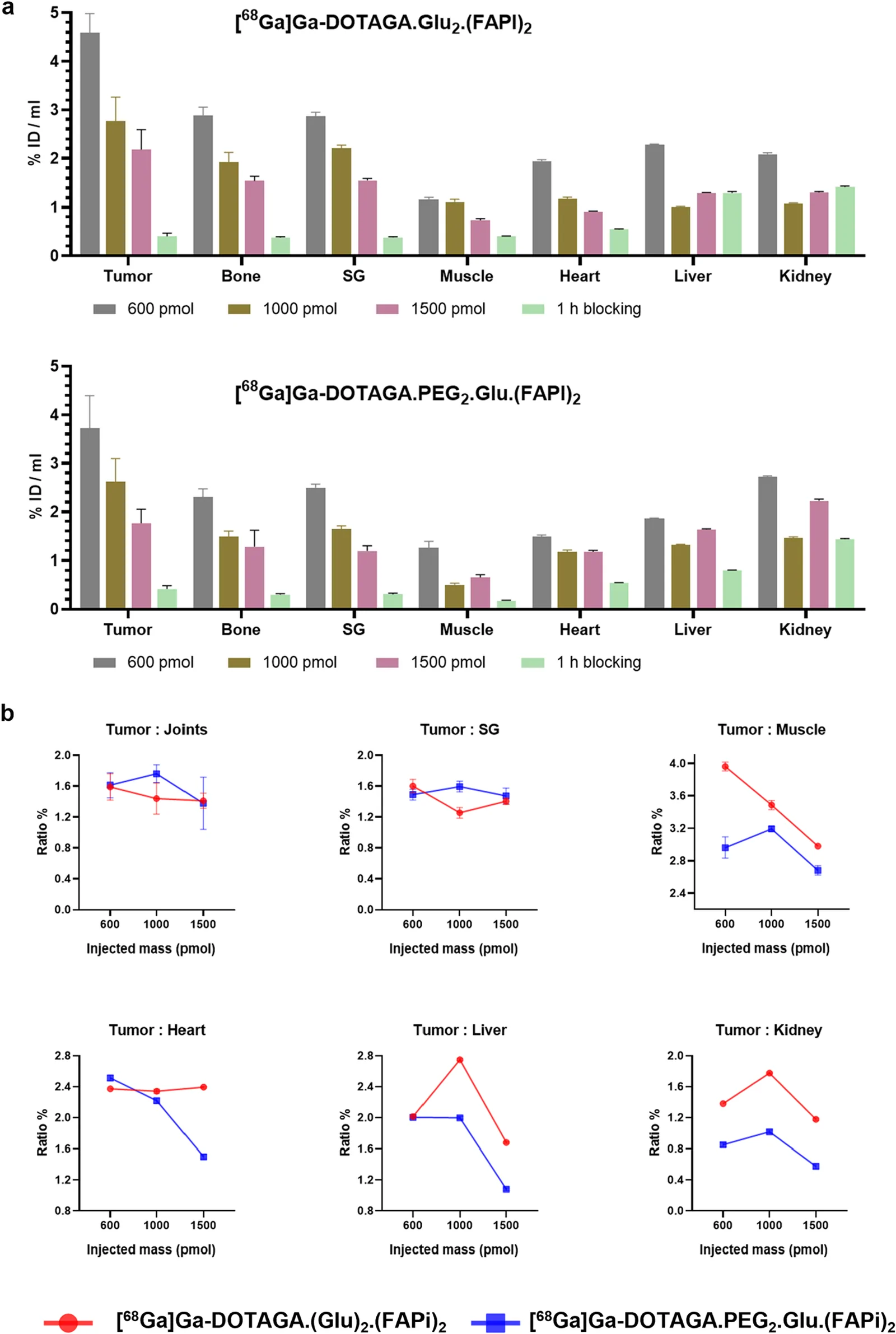
(a) PET quantification for various organs of mice bearing PC3 tumors 1h p.i. of [^68^Ga]­Ga-DOTAGA.(Glu)_2_.(FAPi)_2_ or [^68^Ga]­Ga-DOTAGA.PEG_2_.Glu.(FAPi)_2_. (b) Tumor to organ (Joints, SG, muscle, heart, liver and kidney) ratios from PET quantification data. Tumors (T) are indicated by white arrows, and SG denotes salivary glands.

## Discussion

Five novel FAPi-homodimers featuring different linkers/spacers and chelators were synthesized and evaluated for their radiolabeling efficiency with gallium-68 and lutetium-177. The compounds included NPyr-, Glu_2_-, and PEG_2_.Glu-linked dimers, each prepared using either DOTAGA or DO3A as the chelating moiety. Their synthesis and radiochemical behavior revealed significant differences in efficiency and versatility depending on the linker architecture and chelator employed.

The NPyr-linked derivatives, DOTAGA.NPyr.(FAPi)_2_ **8** and DO3A.NPyr.(FAPi)_2_ **11**, were obtained over seven steps with relatively low overall yields (4%), mainly resulting from the coupling of DOTAGA­(*^t^* Bu)_4_ **7** to the sterically hindered secondary amine NPyr.(FAPi)_2_ **6**. These challenges highlight the limitations of using secondary amine linkers in such dimeric systems.

In contrast, the (Glu)_2_-linked dimers, DOTAGA.(Glu)_2_.(FAPi)_2_ **18** and DO3A.(Glu)_2_.(FAPi)_2_ **19**, were obtained in six steps with improved yields of 12% and 7%, respectively. These reactions benefited from the use of primary amines, resulting in better coupling efficiency. Moreover, the DOTAGA-based derivative **18** yielded three times more product than the NPyr analog **8**, emphasizing the impact of reduced steric hindrance. A side product consistently observed in the final TFA deprotection step across multiple syntheses and it was identified *via* LC/MS as being 74.1 Da heavier than the expected deprotected product, suggesting the formation of a *tert*-butyl amide (NP) *via* side reaction of the cyano group. To suppress this undesired transformation, 5% MeCN was added to the TFA/TIPS/H_2_O mixture. This modification likely scavenges the reactive cyano intermediate, thereby preventing its conversion to the NP byproduct. For **18**, this adjustment improved the yield over the final steps from 5% to 16%, though no benefit was seen for the DO3A-based analog **19**, suggesting structure-dependent side reaction profiles.

The PEG_2_.Glu-linked compound DOTAGA.PEG_2_.Glu.(FAPi)_2_ **25** was synthesized in five steps with the highest overall yield (17%). The flexible and hydrophilic PEG_2_ spacer likely improved the solubility and minimized steric hindrance during coupling and deprotection. Attempts to synthesize the corresponding DO3A-based analog under identical conditions failed, consistent with previous observations that DO3A derivatives often show lower coupling efficiency.

Radiolabeling with gallium-68 and lutetium-177 revealed additional structure–activity trends. In line with prior studies, labeling of the DOTAGA derivatives was most efficient in HEPES buffer, while negligible complexation was observed in AmOAc or NaOAc for both **8** and **18**.^21^ [^68^Ga]­Ga-DOTAGA.NPyr.(FAPi)_2_ **8** achieved RCCs of ∼80–85% in HEPES with increasing precursor amounts, while DOTAGA.(Glu)_2_.(FAPi)_2_ **18** reached >85% with as little as 5 nmol. Interestingly, ethanol, known to enhance DOTA-based radiolabeling, did not render RCCs quantitative, and the DO3A analog **11** actually showed better labeling efficiency, indicating that steric hindrance was not the limiting factor for **8**.

A particularly remarkable result was obtained for [^68^Ga]­Ga**-**DOTAGA.PEG_2_.Glu.(FAPi)_2_ **25**, which showed RCC ≥ 95% in all three buffer systems (HEPES, AmOAc and NaOAc). Efficient labeling was achieved in AmOAc even at reduced precursor concentrations (5 nmol), suggesting that the longer PEG_2_ spacer of **25** reduces steric hindrance and thus allows complexation in a broader range of conditions.

In contrast, [^68^Ga]­Ga-DO3A.(Glu)_2_.(FAPi)_2_ **19** labeled slowly despite good final RCCs, and was not investigated further with lutetium-177 due to its slow kinetics and limited precursor availability.

Lutetium-177 labeling was achieved with consistently high yields for the DOTAGA-based compounds **8**, **18**, and **25**. All reactions were performed in 1 M HEPES (pH 5.5) with quantitative RCCs ≥ 99% within 5 min, even with as little as 2 nmol precursor per 50 MBq. The results suggest that precursor amount can be reduced further without affecting yield during scale-up, particularly given the longer half-life of lutetium-177 (6.7 days).

This study highlights the impact of the linker design and the chelator choice on both synthetic accessibility and radiolabeling performance of FAPi-homodimers. DOTAGA-based compounds, especially those incorporating flexible PEG linkers, offer superior yields and broader radiochemical compatibility compared to DO3A analogs. These findings provide a rational framework for the design of the next-generation FAP-targeting agents with optimized properties for theranostic applications.

Following the successful synthesis and radiolabeling of the FAPi-homodimers, we evaluated their lipophilicity to better understand how linker architecture and chelator selection influence key physicochemical properties relevant to their *in vivo* pharmacokinetics. These studies complement the observed differences in radiochemical performance and offer insight into the impact of linker architecture and chelator selection on molecular hydrophilicity.

All radiolabeled compounds demonstrated high hydrophilicity, with logD_7.4_ values consistent with renal clearance profiles expected of FAPi-targeted imaging agents. Among the tested series, [^177^Lu]­Lu-DOTAGA.(Glu)_2_.(FAPi)_2_ ([^177^Lu]­Lu-**18)** exhibited the lowest log *D*_7.4_ (−3.49 ± 0.25), closely matching previously reported values for [^177^Lu]­Lu-DOTAGA.Glu.(FAPi)_2_ (log *D*_7.4_ = −3.00 ± 0.10), suggesting that the incorporation of a second glutamate moiety in the dimeric system preserved the compound’s favorable hydrophilic profile.^21^ Interestingly, the addition of this second Glu unit, which introduces an extra free carboxylic acid, also alters the net charge at physiological pH. However, this modification did not result in a substantial increase in hydrophilicity, indicating that charge alone does not dominate lipophilic behavior, and other structural factors such as overall molecular size, spatial conformation, and chelator environment also contribute significantly.

In contrast, the most lipophilic dimer was [^68^Ga]­Ga-DO3A.NPyr.(FAPi)_2_ ([^68^Ga]­Ga-**11**), which also demonstrated the lowest radiolabeling efficiency among the evaluated compounds. This may reflect a reduced overall charge density and lower polarity, as well as the absence of additional acidic groups such as those introduced by the glutamate linkers. Conversely, the PEG_2_.Glu-linked derivative [^68^Ga]­Ga-DOTAGA.PEG_2_.Glu.(FAPi)_2_ ([^68^Ga]­Ga-**25**) maintained high hydrophilicity, attributed to the flexible and hydrophilic nature of the PEG moiety, which enhances solubility without significantly increasing molecular weight or complexity.

The lipophilicity data support the synthetic and radiolabeling trends observed earlier: compounds incorporating DOTAGA and flexible, hydrophilic linkers (*e.g.*, (Glu)_2_ or PEG_2_.Glu) not only demonstrated superior synthetic accessibility and radiolabeling efficiency but also retained favorable physicochemical characteristics for *in vivo* applications. These features are likely to translate into improved pharmacokinetics and biodistribution, as suggested by previous *in vivo* imaging studies on similar structures.^31^

Although the addition of a second glutamate residue was initially expected to decrease lipophilicity, the experimental data indicate that such modifications result in only modest changes to logD_7.4_ values. This suggests a plateauing effect in the hydrophilic tuning of FAPi dimers, whereby additional acidic groups provide diminishing returns beyond a certain threshold of polarity. Nevertheless, these variations could still influence biodistribution patterns, particularly regarding renal excretion or background tissue uptake.

All the newly synthesized FAPi homodimers exhibited subnanomolar inhibitory potency (IC_50_) against FAP, confirming their exceptionally high affinity. This potency was comparable to that of the second-generation dimers previously developed by our group.^21^ In addition to high binding affinity, the compounds demonstrated remarkable selectivity over related serine proteases. Selectivity indices (SI) for FAP over PREP and DPP4 consistently exceeded 1000, highlighting the high target specificity of these ligands.

[^68^Ga]­Ga-DOTAGA.(Glu)_2_.(FAPi)_2_ **18** and [^68^Ga]­Ga-DOTAGA.PEG_2_.Glu.(FAPi)_2_ **25** emerged as the lead candidates from previous investigations (synthesis yield, radiolabeling, hydrophilicity and *in vitro* studies) and were further evaluated in saturation binding assays *in vitro* in CAFs. Although direct comparison between the enzymatic and saturation assays is not feasible, the observed affinity trends were consistent across both methods. Interestingly, while the *B*_max_ values for all previously studied first- and second-generation FAPi ligands were approximately 0.5–0.6 nM, the third-generation compounds exhibited nearly double these values^10,17^ (Table S2). Despite this increase in *B*_max_, cellular binding assays showed that [^68^Ga]­Ga-DOTAGA.(Glu)_2_.(FAPi)_2_ **18** had a total cell-associated activity of ∼40%, similar to that of [^68^Ga]­Ga-DOTAGA.Glu.(FAPi)_2_. In contrast, [^68^Ga]­Ga-DOTAGA.PEG_2_.Glu.(FAPi)_2_ **25** demonstrated lower binding, with ∼25% of the total added activity associated with the cells. The underlying cause for these observations remains to be elucidated. However, these findings suggest that the internalization mechanism of FAP is complex and merits further investigation.

In our previous investigation of circulating FAP and the pharmacokinetics of FAPi-based radioligands, we highlighted the critical role of precise dosing in achieving optimal *in vivo* efficacy in PC3 tumor bearing mice.^31^ Our findings demonstrated that accurate control of the administered mass is essential for maximizing both diagnostic accuracy and therapeutic effectiveness. In this study, we did not employ the full range of injected masses, from very low to very high (100–1500 pmol), as in our previous work.^31^ Instead, we selected 600 pmol as the optimal injected dose for dimers to maximize tumor uptake in PC3 tumor bearing mice. To further investigate the impact of the increasing cold mass on the pharmacokinetics of the radioligands, we additionally administered higher doses of 1000 and 1500 pmol. This allowed us to assess both the extent and nature of the pharmacokinetic alterations induced by the elevated amounts of the nonradioactive ligand. [^68^Ga]­Ga-DOTAGA.(Glu)_2_.(FAPi)_2_ **18** demonstrated higher tumor uptake (PET imaging) at the optimal administered dose, along with lower background uptake across all injected tissues, when compared to [^68^Ga]­Ga-DOTAGA.PEG_2_.Glu.(FAPi)_2_ **25**. These properties resulted in significantly improved tumor-to-organ ratios for [^68^Ga]­Ga-DOTAGA.(Glu)_2_.(FAPi)_2_ **18**. Current preclinical data suggest that this radioligand holds strong potential as a candidate for therapeutic applications. Future studies will also encompass comprehensive therapeutic efficacy evaluations and detailed dosimetry analyses to further substantiate the therapeutic potential of these FAPi homodimers.

## Conclusion

In conclusion, this study presents the successful synthesis, radiochemical evaluation, and initial preclinical profiling of five novel FAPi-homodimers incorporating various linkers/spacer units and chelators. Among these, the DOTAGA-based compounds featuring (Glu)_2_ and PEG_2_.Glu linkers demonstrated superior synthetic yields, robust and efficient radiolabeling with gallium-68 and lutetium-177, and favorable hydrophilicity contributing to renal clearance. The PEG_2_.Glu derivative offered enhanced radiochemical versatility, while the (Glu)_2_-linked analog showed enhanced tumor uptake and tumor-to-organ ratios *in vivo*, highlighting its therapeutic potential. DO3A-based counterparts consistently underperformed, emphasizing the importance of chelator selection. These findings established a clear structure–activity relationship between linker/spacer design, chelator chemistry, and radioligand performance, providing a rational framework for the development of the next-generation of FAPi-radiopharmaceuticals with optimized properties for theranostic applications.

## Experimental Section

### Chemicals and Equipment

All chemicals and reagents used in this study were purchased from commercial suppliers and used without further purification as described in Martin et al.^21,38^

The instruments and analytical equipment employed for synthesis, radiolabeling, characterization and PET/CT imaging are described in detail in the Supporting Information.

Mice were purchased from Janvier Laboratories (Rue du Genest, 53940 Le Genest-Saint-Isle, France), pentobarbital natrium (150 mg/kg) from Streuli Pharma SA (Uznach, Switzerland). The radiotracer solutions for the *in vivo* studies were prepared by dilution with 0.9% NaCl (Bichsel AG, Interlaken, Switzerland).

### Organic Synthesis

The synthetic strategy for the third-generation FAPi homodimers was based on the previously reported procedure for second-generation derivatives, with modifications introduced at the linker moiety.^21,38^ All other key reaction steps, purification procedures, and characterization methods were performed as described previously to ensure consistency and comparability.^21,38^

#### Synthesis of DOTAGA.NPyr.(FAPi)_2_ and DO3A.NPyr.(FAPi)_2_

##### Dibenzyl (*R*)-2,2′-((1-(*tert*-Butoxycarbonyl)­pyrrolidin-3-yl)­azanediyl)-diacetate (**3**, Boc-NPyr­(OBzl)_2_)

(*S*)-1-Boc-3-aminopyrrolidine **1** (1.07 g, 5.74 mmol, 1.00 equiv) and *N*,*N*-diisopropylethylamine (DIPEA, 1.5 mL) were dissolved in MeCN (6 mL). After 60 min, a solution of benzyl bromoacetate **2** (1.74 g, 7.55 mmol, 1.32 equiv) in MeCN (6 mL) was slowly added and stirred at room temperature (RT) for an additional 2 h. MeCN was removed under reduced pressure. Subsequent column chromatography (CHCl_3_/MeOH (30:1) + 1% triethylamine (TEA)) yielded Boc-NPyr­(OBzl)_2_ **3** (1.31 g, 2.71 mmol, 47%) as a colorless oil. ^1^H NMR (400 MHz, CDCl_3_) δ = 7.42–7.28 (m, 10H), 5.13 (s, 4H), 3.76–3.38 (m, 7H), 3.21 (td, *J* = 10.6, 6.7 Hz, 1H), 3.16–3.03 (m, 1H), 2.08–1.94 (m, 1H), 1.83–1.69 (m, 1H), 1.44 (s, 9H). MS (ESI^+^): *m*/*z* (%) = 383.2 (45, [M–Boc + H]^+^), 483.2 (100, [M + H]^+^), 484.2 (30, [M + H]^+^), calculated for C_27_H_34_N_2_O_6_: 482.24 [M].

##### (*R*)-2,2′-((1-(*tert*-Butoxycarbonyl)­pyrrolidin-3-yl)­azanediyl)­diacetic acid (**4**, Boc-NPyr)

Boc-NPyr­(OBzl)_2_ **3** (1.21 g, 2.51 mmol, 1.00 equiv) was dissolved in dry MeOH (8 mL) and palladium on carbon (10 wt % Pd, 53 mg, 50 μmol, 0.02 equiv) was added. It was stirred at RT for 2 days under hydrogen atmosphere. It was filtered through Celite and washed with MeOH. The solvent was removed in vacuo. Boc-NPyr **4** (643 mg, 2.13 mmol, 85%) was obtained as a colorless solid. ^1^H NMR (400 MHz, MeOD) δ = 3.75–3.45 (m, 6H), 3.35 (s, 1H), 3.31–3.11 (m, 2H), 2.15–2.05 (m, 1H), 1.95–1.79 (m, 1H), 1.46 (s, 9H). MS (ESI+): *m*/*z* (%) = 247.0 (100, [M–*^t^* Bu + H]^+^), 303.1 (36, [M + H]^+^), 605.3 (23, [2M + H]^+^), calculated for C_13_H_22_N_2_O_6_: 302.15 [M].

##### *tert*-Butyl (*R*)-3-(Bis­(2-((4-((4-((2-((*S*)-2-cyano-4,4-difluoropyrrolidin-1-yl)-2-oxoethyl)­carbamoyl) quinolin-6-yl)­oxy)­butyl)­amino)-2-oxoethyl)­amino)­pyrrolidine-1-carboxylate (**5**, Boc-NPyr.(FAPi)_2_)

Boc-NPyr **4** (30.2 mg, 100 μmol, 1.00 equiv), 1-hydroxybenzotriazole (HOBt, 36.0 mg, 266 μmol, 2.66 equiv) and 1-ethyl-3-(3-(dimethylamino)­propyl)­carbodiimide hydrochloride (EDC*HCl, 50.0 mg, 260 μmol, 2.60 equiv) were dissolved in dry *N*,*N*-dimethylformamide (DMF, 3 mL) and stirred under argon atmosphere at 30 °C for 60 min. Then, a solution of FAPi-NH_2_*TFA (110 mg, 202 μmol, 2.02 equiv) and DIPEA (51.0 μL, 300 μmol, 3.00 equiv) in DMF (2 mL) was added and the solution was stirred at 30 °C for another 3.5 h. HOBt (8.5 mg, 62.9 μmol, 0.63 equiv) and EDC*HCl (12.0 mg, 62.6 μmol, 0.63 equiv) was then added, followed by a solution of FAPi-NH_2_*TFA (25.0 mg, 45.8 μmol, 0.46 equiv) and DIPEA (17.0 μL, 100 μmol, 1.00 equiv) in dry DMF (1 mL) 30 min later. After stirring at 30 °C overnight, the additions of HOBt (8.5 mg, 62.9 μmol, 0.63 equiv), EDC*HCl (12.0 mg, 62.6 μmol, 0.63 equiv), and 30 min later of FAPi-NH_2_*TFA (16.0 mg, 29.3 μmol, 0.29 equiv) with DIPEA (17.0 μL, 100 μmol, 1.00 equiv) in dry DMF (1 mL) were repeated one more time. Stirring continued for another 5 h at 30 °C and then the solvent was removed in vacuo. After column chromatography (CHCl_3_/MeOH/TEA, 100:7.5–10:1) Boc-NPyr.(FAPi)_2_ **5** (102 mg, 90.3 μmol, 90%) was obtained as a yellow oil. MS (ESI^+^): *m*/*z* (%) = 358.6 (86, [M – *^t^* Bu + H]^3+^), 372.2 (58, [M–*^t^* Bu + MeCN + H]^3+^), 377.3 (100, [M + H]^3+^), 390.3 (68, [M + MeCN + H]^3+^), 515.3 (36, [M – Boc + H]^2+^), 537.5 (8, [M – *^t^* Bu + H]^2+^), 565.5 (84, [M + H]^2+^), 1129.6 (28, [M + H]^+^), 1130.6 (17, [M + H]^+^), calculated for C_55_H_64_F_4_N_12_O_10_: 1128.48 [M].

##### 6,6′-((((2,2′-(((*R*)-Pyrrolidin-3-yl)­azanediyl)­bis­(acetyl))­bis­(azanediyl))­bis­(butane-4,1-diyl)) bis­(oxy))­bis­(*N*-(2-((*S*)-2-cyano-4,4-difluoropyrrolidin-1-yl)-2-oxoethyl)­quinoline-4-carboxamide) (**6**, NPyr.(FAPi)_2_)

Boc-NPyr.(FAPi)_2_ **5** (102 mg, 90.3 μmol) was stirred with Milli-Q water (50 μL), triisopropylsilane (TIPS, 50 μL), and TFA (1.9 mL) at RT for 1 h. Then, MeOH (10 mL) was added and removed again in vacuo which was repeated five times. A yellow oil was obtained which was used in the next step without further purification. MS (ESI^+^): *m*/*z* (%) = 344.1 (100, [M + H]^3+^), 357.6 (45, [M + MeCN + H]^3+^), 515.5 (18, [M + H]^2+^), 1029.5 (3, [M + H]^+^), calculated for C_50_H_56_F_4_N_12_O_8_: 1028.43 [M].

##### 2,2′,2′′-(10-(4-((*R*)-3-(Bis­(2-((4-((4-((2-((*S*)-2-cyano-4,4-difluoropyrrolidin-1-yl)-2-oxoethyl)­carbamoyl)­quinolin-6-yl)­oxy)­butyl)­amino)-2-oxoethyl)­amino)­pyrrolidin-1-yl)-1-carboxy-4-oxobutyl)-1,4,7,10-tetraazacyclododecane-1,4,7-triyl)­triacetic acid (**8**, DOTAGA.NPyr.(FAPi)_2_)

DOTAGA­(*^t^* Bu)_4_ **7** (50.0 mg, 71.3 μmol, 1.43 equiv), *N*-hydroxysuccinimide (NHS, 16.4 mg, 142 μmol, 2.84 equiv) and 2-(1H-benzotriazol-1-yl-)-1,1,3,3-tetramethyluronium hexafluorophosphate (HBTU, 54.1 mg, 143 μmol, 2.86 equiv) were dissolved in dry DMF (1 mL) and shaken overnight at 30 °C under argon atmosphere. NHS (8.2 mg, 71.2 μmol, 1.42 equiv) and HBTU (27.0 mg,71.2 μmol, 1.42 equiv) were added again. Four hours later, a solution of NPyr.(FAPi)_2_ **6** (51.5 mg, 50.0 μmol, 1.00 equiv) and DIPEA (40 μL, 235 μmol, 4.70 equiv) in dry DMF (1 mL) was added. Stirring continued for 2 days at 40 °C and then all solvents were completely removed in vacuo. A yellow oil was obtained and used without further purification.

Twenty-five μL of Milli-Q water, 50 μL of TIPS, 100 μL MeCN and 1 mL of TFA (TFA/MeCN/TIPS/H_2_O, 100:10:5:2.5) was added and the solution was stirred at RT for 5 h. Then the solvents were removed under reduced pressure *via* codistillation with MeOH (3 × 10 mL). The crude product was purified by semipreparative RP-HPLC (25–27% MeCN in 20 min, *t*_R_ = 18–20 min). 8.3 mg (5.6 μmol, 11%) DOTAGA.NPyr.(FAPi)_2_ **8** were obtained as a yellow solid (purity = 99.6%: cf. analytical RP-HPLC in S1). MS (ESI^+^): *m*/*z* (%) = 372.6 (100, [M + H]^4+^), 382.9 (38, [M + MeCN + H]^4+^), 496.6 (76, [M + H]^3+^), 744.4 (5, [M + H]^2+^), calculated for C_69_H_86_F_4_N_16_O_17_: 1486.63 [M].

##### tri-*tert*-Butyl 2,2′,2′′-(10-(2-((2,5-dioxopyrrolidin-1-yl)­oxy)-2-oxoethyl)-1,4,7,10-tetraazacyclodo-decane-1,4,7-triyl)­triacetate (**10**, DOTA­(*^t^* Bu)_3_-NHS)

DOTA-tris­(*tert*-butyl ester) **9** (573 mg, 1.00 mmol, 1.00 equiv) and HBTU (417 mg, 1.10 mmol, 1.10 equiv) were dissolved in dry MeCN (12 mL) and dry DMF (5 mL) and then NHS (127 mg, 1.10 mmol, 1.10 equiv) was added under argon atmosphere. After stirring at 30 °C for 6 h, HBTU (190 mg, 501 μmol, 0.50 equiv) and NHS (57.5 mg, 500 μmol, 0.50 equiv) were added and stirring was continued overnight. After all solvents were removed in vacuo, purification by column chromatography (dichloromethane (DCM)/MeOH, 10:1) gave **10** as a colorless solid (643 mg, 961 μmol, 96%). ^1^H NMR (400 MHz, CDCl3): δ [ppm] = 2.97–2.77 (m, 12H), 2.66–2.02 (m, 16H), 1.44 (s, 27H). MS (ESI^+^): *m*/*z* (%) = 335.7 (100, [M + H]^2+^), 670.4 (50, [M + H]^+^), 671.4 (18, [M + H]^+^), calculated for C_32_H_55_N_5_O_10_: 669.39 [M].

##### 2,2′,2′′-(10-(2-((*R*)-3-(Bis­(2-((4-((4-((2-((*S*)-2-cyano-4,4-difluoropyrrolidin-1-yl)-2-oxoethyl)­carbamoyl)­quinolin-6-yl)­oxy)­butyl)­amino)-2-oxoethyl)­amino)­pyrrolidin-1-yl)-2-oxoethyl)-1,4,7,10-tetraazacyclododecane-1,4,7-triyl)­triacetic Acid (**11**, DO3A.NPyr.(FAPi)_2_)

DOTA­(*^t^* Bu)_3_-NHS **10** (33.5 mg, 50.0 μmol, 1.25 equiv) and NPyr.(FAPi)_2_ **6** (41.2 mg, 40.0 μmol, 1.00 equiv) were dissolved in dry DMF (1 mL) and DIPEA (50 μL) was added. The reaction was stirred under argon atmosphere at 40 °C for 3 days and then all solvents were completely removed in vacuo. A yellow oil was obtained which was directly used without further purification.

After addition of 50 μL Milli-Q water, 50 μL TIPS, and 1.5 mL TFA (TFA/TIPS/H_2_O, 94:3:3) it was stirred at RT for 12 h. MeOH (10 mL) was added removed again in vacuo which was repeated five times. The crude product was purified by semipreparative RP-HPLC (21–22% MeCN in 20 min, *t*_R_ = 18.5–19.5 min). DO3A.NPyr.(FAPi)_2_ **11** (5.6 mg, 4.0 μmol, 10%) was obtained as a yellow solid (purity = 95.2%: cf. analytical RP-HPLC in S2). MS (ESI^+^): *m*/*z* (%) = 354.6 (95, [M + H]^4+^), 364.8 (59, [M + MeCN + H]^4+^), 472.6 (100, [M + H]^3+^), 708.6 (13, [M + H]^2+^), 1415.5 (5, [M + H]^+^), calculated for C_66_H_82_F_4_N_16_O_15_: 1414.61 [M].

#### Synthesis of DOTAGA.(Glu)_2_.(FAPi)_2_ and DO3A.(Glu)_2_.(FAPi)_2_

##### Dibenzyl ((*S*)-4-((((9H-Fluoren-9-yl)­methoxy)­carbonyl)­amino)-5-(*tert*-butoxy)-5-oxopentanoyl)-l-glutamatetri-*tert*-Butyl (**14**, Fmoc-Glu­(O*^t^* Bu).Glu­(OBzl)_2_)

Fmoc-Glu-O*^t^* Bu **13** (400 mg, 940 μmol, 1.00 equiv) was dissolved in dry DMF (2 mL). DIPEA (200 μL, 1.18 mmol, 1.26 equiv) and 1-[bis­(dimethylamino)­methylene]-1*H*-1,2,3-triazolo­[4,5-*b*]­pyridinium 3-oxide hexafluorophosphate (HATU, 393 mg, 1.03 mmol, 1.10 equiv) was added and the solution was stirred under argon atmosphere at RT. After 1 h, a solution of dibenzyl glutamate **12** (385 mg, 1.18 mmol, 1.26 equiv) and DIPEA (352 μL, 2.07 mmol, 2.17 equiv) in dry DMF (1 mL) was added and stirred overnight at RT. HATU (357 mg, 939 μmol, 1.00 equiv) and DIPEA (156 μL, 917 μmol, 0.97 equiv) was added again. After three more days of stirring at RT, HATU (357 mg, 939 μmol, 1.00 equiv) and 1 h later a solution of dibenzyl glutamate **12** (154 mg, 470 μmol, 0.50 equiv) and DIPEA (160 μL, 941 μmol, 1.00 equiv) in DMF (0.5 mL) was added. The solvent was removed under reduced pressure after another day at RT and the product was purified by column chromatography (cyclohexane/ethyl acetate (CH/EA), 3:1). Fmoc-Glu­(O*^t^* Bu).Glu­(OBzl)_2_ **14** (657 mg, 894 μmol, 95%) was obtained as a yellowish solid. ^1^H NMR (400 MHz, CDCl_3_): δ [ppm] = 7.76 (dd, *J* = 7.5, 0.9 Hz, 2H), 7.60 (d, *J* = 7.4 Hz, 2H), 7.43–7.36 (m, 2H), 7.36–7.27 (m, 12H), 6.50 (d, *J* = 7.7 Hz, 1H), 5.54 (d, *J* = 8.1 Hz, 1H), 5.14 (s, 2H), 5.07 (s, 2H), 4.67 (td, *J* = 7.8, 5.0 Hz, 1H), 4.45–4.34 (m, 2H), 4.27–4.17 (m, 2H), 2.53–2.32 (m, 2H), 2.31–2.14 (m, 4H), 2.11–1.81 (m, 2H), 1.46 (s, 9H). MS (ESI^+^): *m*/*z* (%) = 679.2 (27, [M – *^t^* Bu + H]^+^), 680.3 (11, [M – *^t^* Bu + H]^+^), 735.5 (100, [M + H]^+^), 736.2 (15, [M + H]^+^), calculated for C_43_H_46_N_2_O_9_: 734.32 [M].

##### ((*S*)-4-((((9H-Fluoren-9-yl)­methoxy)­carbonyl)­amino)-5-(*tert*-butoxy)-5-oxopentanoyl)-l-glutamic Acid (**15**, Fmoc-Glu­(O*^t^* Bu).Glu)

Fmoc-Glu­(O*^t^* Bu).Glu­(OBzl)_2_ **14** (200 mg, 272 μmol, 1.00 equiv) was dissolved in dry tetrahydrofuran (THF, 5 mL) and palladium on carbon (10 wt % Pd, 29.0 mg, 27.2 μmol, 0.10 equiv) was added. The suspension was stirred overnight under hydrogen atmosphere and filtered through Celite. The residue was washed with THF and the solvent was removed under reduced pressure. Fmoc-Glu­(O*^t^* Bu).Glu **15** (150 mg, 270 μmol, 99%) was obtained as a colorless solid. ^1^H NMR (400 MHz, DMSO-*d*_6_): δ [ppm] = 8.32 (s, 1H), 8.11 (d, *J* = 7.7 Hz, 1H), 7.93–7.83 (m, 2H), 7.76–7.67 (m, 2H), 7.46–7.28 (m, 4H), 6.87 (s, 1H), 4.35–4.09 (m, 3H), 3.99–3.70 (m, 2H), 2.31–2.16 (m, 5H), 2.01–1.88 (m, 1H), 1.83–1.67 (m, 1H), 1.49–1.39 (m, 1H), 1.39 (s, 9H). MS (ESI^+^): *m*/*z* (%) = 499.05 (57, [M – *^t^* Bu + H]^+^), 500.2 (11, [M – *^t^* Bu + H]^+^), 555.3 (100, [M + H]^+^), 556.2 (21, [M + H]^+^), calculated for C_29_H_34_N_2_O_9_: 554.23 [M].

##### *tert*-Butyl (2*S*,7*R*)-2-((((9H-Fluoren-9-yl)­methoxy)­carbonyl)­amino)-10-((4-((4-((2-((S)-2-cyano-4,4-difluoropyrrolidin-1-yl)-2-oxoethyl)­carbamoyl)­quinolin-6-yl)­oxy)­butyl)­amino)-7-((4-((4-((2-((S)-2-cyano-4,4-difluoropyrrolidin-1-yl)-2-oxoethyl)­carbamoyl)­quinolin-6-yl)­oxy)­butyl)­carbamoyl)-5,10-dioxodecanoate (**16**, Fmoc-Glu­(O*^t^* Bu).Glu.(FAPi)_2_)

Fmoc-Glu­(O*^t^* Bu).Glu **15** (33.3 mg, 60.0 μmol, 1.00 equiv), HOBt (20.4 mg, 151 μmol, 2.52 equiv) and EDC*HCl (28.8 mg, 150 μmol, 2.50 equiv) were dissolved in dry DMF (2.5 mL) and stirred under argon atmosphere at RT for 1 h. FAPi-NH_2_*TFA (65.4 mg, 120 μmol, 2.00 equiv) and DIPEA (31.0 μL, 182 μmol, 3.03 equiv), dissolved in dry DMF (0.5 mL), was added and stirred at RT overnight. HOBt (7.8 mg, 57.7 μmol, 0.96 equiv) and EDC*HCl (11.4 mg, 59.5 μmol, 0.99 equiv) was added, followed by a solution of FAPi-NH_2_*TFA (16.5 mg, 30.2 μmol, 0.50 equiv) and DIPEA (10.0 μL, 58.8 μmol, 0.98 equiv) in DMF (0.5 mL) 30 min later. One day later, HOBt (3.9 mg, 28.8 μmol, 0.48 equiv) and EDC*HCl (5.7 mg, 29.7 μmol, 0.50 equiv) and after one more hour, additional FAPi-NH_2_*TFA (16.5 mg, 30.2 μmol, 0.50 equiv) and DIPEA (5.0 μL, 29.4 μmol, 0.49 equiv) in DMF (0.5 mL), was added. This step was repeated again the next day. After the final addition the reaction solution was stirred for one more day and then the solvent was subsequently removed under reduced pressure. Column chromatography (CHCl_3_/MeOH, 10:1) yielded Fmoc-Glu­(O*^t^* Bu).Glu.(FAPi)_2_ **16** (86.7 mg, 62.8 μmol, 79%) as a yellowish solid. MS (ESI^+^): *m*/*z* (%) = 461.3 (32, [M + H]^3+^), 691.5 (100, [M + H]^2+^),692.3 (12, [M + H]^2+^), 1382.0 (12, [M + H]^+^), calculated for C_71_H_76_F_4_N_12_O_13_: 1380.56 [M].

##### *tert*-Butyl (2*S*,7*R*)-2-Amino-10-((4-((4-((2-((*S*)-2-cyano-4,4-difluoropyrrolidin-1-yl)-2-oxoethyl)­carbamoyl)­quinolin-6-yl)­oxy)­butyl)­amino)-7-((4-((4-((2-((S)-2-cyano-4,4-difluoropyrrolidin-1-yl)-2-oxoethyl)­carbamoyl)­quinolin-6-yl)­oxy)­butyl)­carbamoyl)-5,10-dioxodecanoate (**17**, Glu­(O*^t^* Bu).Glu.(FAPi)_2_)

Fmoc-Glu­(O*^t^* Bu).Glu.(FAPi)_2_ **16** (72.2 mg, 52.3 μmol, 1.00 equiv) was dissolved in dry DMF (1 mL) and piperidine (100 μL) was added. The solution was stirred at RT for 90 min. Then, the solvents were completely removed under reduced pressure. Glu­(O*^t^* Bu).Glu.(FAPi)_2_ **17** was obtained as a yellow oil in quantitative yield and used directly in the next step without further purification. MS (ESI^+^): *m*/*z* (%) = 387.1 (99, [M + H]^3+^), 580.4 (37, [M + H]^2+^), 1159.3 (4, [M + H]^+^), calculated for C_56_H_66_F_4_N_12_O_11_: 1158.49 [M].

##### 2,2′,2′′-(10-(4-(((*S*)-4-(((*S*)-1,5-Bis­((4-((4-((2-((*S*)-2-cyano-4,4-difluoropyrrolidin-1-yl)-2-oxoethyl)­carbamoyl)­quinolin-6-yl)­oxy)­butyl)­amino)-1,5-dioxopentan-2-yl)­amino)-1-carboxy-4-oxobutyl)­amino)-1-carboxy-4-oxobutyl)-1,4,7,10-tetraazacyclododecane-1,4,7-triyl)­triacetic acid (**18**, DOTAGA.(Glu)_2_.(FAPi)_2_)

DOTAGA­(*^t^* Bu)_4_ **7** (36.3 mg, 45.5 μmol, 1.25 equiv) and HATU (27.7 mg, 72.9 μmol, 2.00 equiv) was dissolved in dry DMF (700 μL), DIPEA (9,3 μL, 54.7 μmol, 1.50 equiv) was added and stirred under argon atmosphere at RT for 60 min. Glu­(O*^t^* Bu).Glu.(FAPi)_2_ **16** (42.2 mg, 36.4 μmol, 1.00 equiv) and DIPEA (12.4 μL, 72.9 μmol, 2.00 equiv), dissolved in dry DMF (500 μL) and dry MeCN (500 μL), was added and stirred at RT overnight. The solvents were removed in vacuo and the residue was dissolved in 25 μL Milli-Q water, 25 μL TIPS, 50 μL MeCN and 900 μL TFA (TFA/MeCN/TIPS/H_2_O, 90:5:2.5:2.5) and stirred at RT for 6 h. The solvents were removed *via* codistillation with MeOH (3 × 10 mL). Subsequent semipreparative RP-HPLC (23–25% MeCN in 20 min, *t*_R_ = 15–16 min) yielded DOTAGA.(Glu)_2_.(FAPi)_2_ **18** (9.1 mg, 5.8 μmol, 16%) as a yellow solid (purity = 97.8%: cf. analytical RP-HPLC in S3). MS (ESI^+^): *m*/*z* (%) = 391.1 (78, [M + H]^4+^), 401.2 (19, [M + MeCN + H]^4+^), 521.3 (100, [M + H]^3+^), 781.8 (6, [M + H]^2+^), 1561.7 (3, [M + H]^+^). calculated for C_71_H_88_F_4_N_16_O_20_: 1560.63 [M].

##### 2,2′,2′′-(10-(2-(((*S*)-4-(((*S*)-1,5-Bis­((4-((4-((2-((*S*)-2-cyano-4,4-difluoropyrrolidin-1-yl)-2-oxoethyl)­carbamoyl)­quinolin-6-yl)­oxy)­butyl)­amino)-1,5-dioxopentan-2-yl)­amino)-1-carboxy-4-oxobutyl)­amino)-2-oxoethyl)-1,4,7,10-tetraazacyclododecane-1,4,7-triyl)­triacetic acid (**19**, DO3A.(Glu)_2_.(FAPi)_2_)

DOTA­(*^t^* Bu)_3_-NHS **9** (26.0 mg, 38.9 μmol, 1.00 equiv) and Glu­(O*^t^* Bu).Glu.(FAPi)_2_ **16** (45.1 mg, 38.9 μmol, 1.00 equiv) was dissolved in dry DMF (1.5 mL) and DIPEA (15.0 μL, 88.2 μmol, 2.27 equiv) was added. After stirring at 40 °C and under argon atmosphere for 3 days, DOTA­(*^t^* Bu)_3_-NHS **9** (6.5 mg, 9.7 μmol, 0.25 equiv) was added and after another 6 h at 40 °C the solvent was removed under reduced pressure.

25 μL Milli-Q water, 25 μL TIPS, 50 μL MeCN and 900 μL TFA (TFA/MeCN/TIPS/H_2_O, 90:5:2.5:2.5) was added and stirred for 6 h at RT. The solvents were removed *via* codistillation with MeOH (3 × 10 mL). After semipreparative RP-HPLC purification (22–23% MeCN in 20 min, *t*_R_ = 13.5–14.5 min) DO3A.(Glu)_2_.(FAPi)_2_ **19** (6.0 mg, 4.0 μmol, 10%) was obtained as a yellow solid (purity = 95.9%: cf. analytical RP-HPLC in S4). MS (ESI^+^): *m*/*z* (%) = 373.1 (84, [M + H]^4+^), 497.2 (100, [M + H]^3+^), 745.7 (5, [M + H]^2+^), calculated for C_68_H_84_F_4_N_16_O_18_: 1488.61 [M].

#### Synthesis of DOTAGA.PEG_2_.Glu.(FAPi)_2_

##### Dibenzyl (1-(9H-Fluoren-9-yl)-3-oxo-2,7,10-trioxa-4-azatridecan-13-oyl)-l-glutamatetri-*tert*-Butyl (**21**, Fmoc-PEG_2_.Glu­(OBzl)_2_)

Fmoc-N-amido-dPEG2 acid **20** (450 mg, 1.13 mmol, 1.00 equiv), HBTU (470 mg, 1.24 mmol, 1.10 equiv), and HOBt (168 mg, 1.24 mmol, 1.10 equiv) were dissolved in dry DMF (9 mL) and DIPEA (240 μL, 1.41 mmol, 1.25 equiv) was added. The colorless solution was stirred under argon atmosphere at RT for 1 h. Then, dibenzyl glutamate **12** (461 mg, 1.41 mmol, 1.25 equiv) dissolved in dry DMF (3 mL) and DIPEA (422 μL, 2.48 mmol, 2.19 equiv) was added. After 1 day at RT, the reaction was completed, and the solvent was removed under reduced pressure. The yellowish oil was purified by column chromatography (DCM/MeOH, 100:2) to yield Fmoc-PEG_2_.Glu­(OBzl)_2_ **21** (795 mg, 1.12 mmol, 99%) as a colorless oil. ^1^H NMR (400 MHz, CDCl3): δ [ppm] = 7.75 (d, *J* = 7.5 Hz, 2H), 7.59 (d, *J* = 7.5 Hz, 2H), 7.43–7.26 (m, 14H), 6.91 (d, *J* = 8.0 Hz, 1H), 5.52 (t, *J* = 5.8 Hz, 1H), 5.12 (s, 2H), 5.07 (d, *J* = 2.5 Hz, 2H), 4.70 (td, *J* = 8.0, 5.1 Hz, 1H), 4.39 (d, *J* = 7.1 Hz, 2H), 4.21 (t, *J* = 7.0 Hz, 1H), 3.77–3.63 (m, 2H), 3.60–3.30 (m, 6H), 2.80 (s, 2H), 2.54–2.28 (m, 4H), 2.28–2.16 (m, 1H), 2.05–1.93 (m, 1H). MS (ESI^+^): *m*/*z* (%) = 709.4 (100, [M + H]^+^), 710.2 (15, [M + H]^+^), calculated for C_41_H_44_N_2_O_9_: 708.30 [M].

##### (1-(9H-Fluoren-9-yl)-3-oxo-2,7,10-trioxa-4-azatridecan-13-oyl)-l-glutamic acid (**22**, Fmoc-PEG_2_.Glu)

Fmoc-PEG_2_.Glu­(OBzl)_2_ **21** (196 mg, 276 μmol, 1.00 equiv) was dissolved in dry THF (2 mL) and palladium on carbon (10 wt % Pd, 30.0 mg, 28.2 μmol, 0.10 equiv) was added. It was then stirred under hydrogen atmosphere at RT for 24 h. The suspension was filtered through Celite, the residue was washed with THF and the solvent was removed under reduced pressure. Fmoc-PEG_2_.Glu **22** (122 mg, 231 μmol, 84%) was obtained as a colorless oil and used in the next step without further purification. ^1^H NMR (400 MHz, CDCl3): δ [ppm] = 7.76 (d, *J* = 7.0 Hz, 2H), 7.62–7.48 (m, 2H), 7.43–7.27 (m, 4H), 6.98 (s, 1H), 5.01 (s, 1H), 4.69–4.55 (m, 1H), 4.47–4.31 (m, 2H), 4.28–4.18 (m, 1H), 3.99–3.27 (m, 8H), 2.80 (s, 2H), 2.62–2.38 (m, 3H), 2.32–2.20 (m, 2H), 2.12–1.99 (m, 1H). MS (ESI^+^): *m*/*z* (%) = 529.3 (100, [M + H]^+^), 530.2 (12, [M + H]^+^), calculated for C_27_H_32_N_2_O_9_: 528.21 [M].

##### (9H-Fluoren-9-yl)­methyl ((*S*)-19-((4-((2-((*S*)-2-cyano-4,4-difluoropyrrolidin-1-yl)-2-oxoethyl)­carbamoyl)­quinolin-6-yl)­oxy)-11-((4-((4-((2-((*S*)-2-cyano-4,4-difluoropyrrolidin-1-yl)-2-oxoethyl)­carbamoyl)­quinolin-6-yl)­oxy)­butyl)­carbamoyl)-9,14-dioxo-3,6-dioxa-10,15-diazanonadecyl)­carbamate (**23**, Fmoc-PEG_2_.Glu.(FAPi)_2_)

Fmoc-PEG_2_.Glu **22** (32.0 mg, 60.5 μmol, 1.00 equiv), HOBt (20.4 mg, 151 μmol, 2.50 equiv) and EDC*HCl (28.8 mg, 150 μmol, 2.48 equiv) were dissolved in dry DMF (1 mL) and stirred at RT under argon atmosphere. After 1 h, a solution of FAPi-NH_2_*TFA (65.4 mg, 120 μmol, 1.98 equiv), DIPEA (30.0 μL, 176 μmol, 2.91 equiv), in dry DMF (500 μL) was added. Three hours later, HOBt (7.8 mg, 57.7 μmol, 0.95 equiv) and EDC*HCl (11.4 mg, 59.5 μmol, 0.98 equiv) was added. Another 30 min later, additional FAPi-NH_2_*TFA (16.5 mg, 30.2 μmol, 0.50 equiv) with DIPEA (10.0 μL, 58.8 μmol, 0.97 equiv) in dry DMF (500 μL) was added and the stirring at RT was continued overnight. After another addition of HOBt (3.9 mg, 28.9 μmol, 0.48 equiv) and EDC*HCl (5.7 mg, 29.7 μmol, 0.49 equiv) and four more hours at RT, the reaction was terminated by removing the solvent under reduced pressure. Purification by column chromatography (CHCl_3_/MeOH, 10:1) yielded Fmoc-PEG_2_.Glu.(FAPi)_2_ **23** (79.1 mg, 58.4 μmol, 97%) as a yellow solid. MS (ESI^+^): *m*/*z* (%) = 452.5 (31, [M + H]^3+^), 678.5 (100, [M + H]^2+^), 679.3 (13, [M + H]^2+^), 1355.9 (9, [M + H]^+^), calculated for C_69_H_74_F_4_N_12_O_13_: 1354.54 [M].

##### (*S*)-2-(3-(2-(2-Aminoethoxy)­ethoxy)­propanamido)-*N* ^1^,*N* ^5^-bis­(4-((4-((2-((S)-2-cyano-4,4-difluoropyrrolidin-1-yl)-2-oxoethyl)­carbamoyl)­quinolin-6-yl)­oxy)­butyl)­pentanediamide (**24**, PEG_2_.Glu.(FAPi)_2_)

Fmoc-PEG_2_.Glu.(FAPi)_2_ **23** (67.0 mg, 49.4 μmol, 1.00 equiv) was dissolved in dry DMF (1 mL) and piperidine (100 μL) was added. The yellowish solution was stirred at RT for 2 h and then the solvents were removed under reduced pressure. PEG_2_.Glu.(FAPi)_2_ **24** was obtained as a yellow oil in quantitative yield and used directly in the next step without further purification. MS (ESI^+^): *m*/*z* (%) = 378.4 (100, [M + H]^3+^), 567.4 (26, [M + H]^2+^), 1133.4 (3, [M + H]^+^), calculated for C_54_H_64_F_4_N_12_O_11_: 1132.48 [M].

##### 2,2′,2′′-(10-((16S)-1-Carboxy-24-((4-((2-((*S*)-2-cyano-4,4-difluoropyrrolidin-1-yl)-2-oxoethyl)­carbamoyl)­quinolin-6-yl)­oxy)-16-((4-((4-((2-((*S*)-2-cyano-4,4-difluoropyrrolidin-1-yl)-2-oxoethyl)­carbamoyl)­quinolin-6-yl)­oxy)­butyl)­carbamoyl)-4,14,19-trioxo-8,11-dioxa-5,15,20-triazatetracosyl)-1,4,7,10-tetraazacyclododecane-1,4,7-triyl)­triacetic acid (**25**, DOTAGA.PEG_2_.Glu.(FAPi)_2_)

DOTAGA­(*^t^* Bu)_4_ **7** (21.0 mg, 30.0 μmol, 1.36 equiv), HATU (12.2 mg, 32.2 μmol, 1.46 equiv) and DIPEA (5.1 μL, 30.0 μmol, 1.36 equiv) was dissolved in dry DMF (1 mL) and stirred at 30 °C and under argon atmosphere for 60 min. A solution of PEG_2_.Glu.(FAPi)_2_ **24** (24.9 mg, 22.0 μmol, 1.00 equiv) and DIPEA (5.1 μL, 30.0 μmol, 1.36 equiv) in dry DMF (500 μL) was added and stirred at RT overnight. HATU (6,1 mg, 16.0 μmol, 0.73 equiv) was added once more and the reaction was stirred for two more days at 30 °C. The solvents were removed under reduced pressure.

The residual yellow oil was dissolved in 25 μL Milli-Q water, 50 μL TIPS, 100 μL MeCN and 1 mL TFA (TFA/MeCN/TIPS/H_2_O, 100:10:5:2.5) and stirred at RT for 6 h. The solvents were removed *via* codistillation with MeOH (3 × 10 mL). After semipreparative RP-HPLC (26–28% MeCN in 20 min, *t*_R_ = 18.5–20.0 min) yielded DOTAGA.PEG_2_.Glu.(FAPi)_2_ **25** (7.3 mg, 4.6 μmol, 21%) as a yellow solid (purity = 97.2%: cf. analytical RP-HPLC in S5). MS (ESI^+^): *m*/*z* (%) = 398.7 (93, [M + H] ^4+^), 531.3 (100, [M + H]^3+^), 796.2 (8, [M + H]^2+^), 1591.9 (3, [M + H]^+^), calculated for C_73_H_94_F_4_N_16_O_20_: 1590.68 [M].

### Radiolabeling

#### ^68^Ga-Radiolabeling

Gallium-68 was eluted from a ITM (Isotope Technologies Munich SE, Munich, Germany) ^68^Ge/^68^Ga-generator with 5 mL of 0.05 M HCl (TraceSelect).^21^ Post processing purification was carried out according to the procedure developed by Eppard et al.^21,39^ Each of the precursor **8**, **11**, **18**, **19** or **25** (2–20 nmol, 2–20 μL 1 mM stock solution) was added to 400 μL buffer. Three different buffer systems were used: 1 M 4-(2-hydroxyethyl)-1-piperazineethansulfonic acid (HEPES pH = 4.5 or 5.5), 1 M ammonium acetate (AmOAc, pH = 4.5) and 1 M sodium acetate (NaOAc, pH = 4.5) as described in Martin et al.^21^ Reaction conditions and purification procedures are described in detail in the Supporting Information.

#### ^68^Ga-Radiolabeling for Cell and Animal Studies

For the cell and animal studies, [^68^Ga]­Ga-DOTAGA.(Glu)_2_.(FAPi)_2_ and [^68^Ga]­Ga-DOTAGA.PEG_2_.Glu.(FAPi)_2_ were prepared within 5 min, using the Modular-Lab PharmTracer module by Eckert&Ziegler (Berlin, Germany).^31,37,40^ Reaction conditions, purification procedures and quality control are as described in Bilinska et al.^31,37^

#### ^177^Lu-Radiolabeling

For labeling with lutetium-177,400 μL 1 M HEPES (pH = 5.5) buffer solution and 2–10 nmol (2.0–10 μL 1 mM stock solution) of precursor **8**, **11**, **18** or **25** was added to 50 MBq ^177^Lu and heated at 95 °C as described in Martin et al.^21^

### Stability Studies in Human Serum (HS) and Phosphate-Buffered Saline (PBS)

The radioligands (approximately 1 nmol; 10 MBq) were incubated in 0.5 mL human serum (HS) and phosphate-buffered saline (PBS) at 37 °C for 120 min (for ^68^Ga-labeled radioligands) and 14 days (for ^177^Lu-labeled radioligands), respectively, as described in Martin et al.^21^

### Lipophilicity/Protein Binding Studies

The lipophilicity (LogD_octanol/PBS,_ pH 7.4) of all ^68^Ga-and ^177^Lu-labeled radioligands was determined by the “shake-flask” method as described in Hofstetter et al.^40^

### *In Vitro*-Inhibition Assays (IC_50_ Measurements)

IC_50_ measurements for FAP and PREP were carried out as previously described.^21,25,41,42^ Detailed experimental procedures, including buffer, concentration and incubation time, are provided in the Supporting Information.

### Cell Lines

The human prostate adenocarcinoma cell line PC3 and the prostate-derived CAF cells were used in the present study. Cultivation conditions, materials and further details are as described in Bilinska et al.^37^

### Saturation Binding Studies/Internalization Studies

For receptor saturation studies, FAP^+^ CAFs were incubated with 0.1 to 10 nM of the radioligands. For internalization studies, ∼2.5 pmol of radioligand was added and cells were incubated for 15, 30, 60, 90, 120, 180, and 240 min at 37 °C, 5% CO_2_ as described in Bilinska et al.^37^

### Animal Models

Male athymic Balb/C nude mice (CByJ.Cg-Foxn1nu/J) (6 weeks/20–25 g) (RRID:IMSR_JAX:002019) were implanted with PC3 cells (3.2 × 10^6^/100 μL PBS) into their right shoulder to develop tumor models as described in Bilinska et al.^37^

For the imaging studies, mice were randomly designated to groups based on their tumor sizes to ensure that each group had a similar size distribution (Animal License: BE98/2021).

### Metabolic Stability

Approximately 1 mL of blood from PC3-mice was collected to heparinized tubes at 10 min after intravenous injection of 600 pmol of [^68^Ga]­Ga-DOTAGA.(Glu)_2_.(FAPi)_2_ and [^68^Ga]­Ga- DOTAGA.PEG_2_.Glu.(FAPi)_2_ (in a total injected volume of 100 μL of 0.9% NaCl) as described in Bilinska et al.^37^

### Small-Animal PET/CT Imaging

Static PET images were obtained 1 h after the injection of 600, 1000, and 1500 pmol (∼5 MBq/100 uL) of [^68^Ga]­Ga**-**DOTAGA.(Glu)_2_.(FAPi)_2_ and [^68^Ga]­Ga**-** DOTAGA.PEG_2_.Glu.(FAPi)_2_ in PC3 tumor bearing mice (*n* = 2–3) as described in Bilinska et al.^31,37^

## Supplementary Material



## Notes

Organic synthesis: HPLC; radio synthesis: Radio HPLC and TLC; complex stabilities; *in vitro*-inhibition assays: Selectivity index of different FAP inhibitors (PDF)

## Abbreviations

FAPi: fibroblast activation protein inhibitors
CAFs: cancer-associated fibroblasts
TRT: targeted radionuclide therapy
PET/CT: positron emission tomography/computed tomography
RCC: radiochemical conversion
TLC: thin-layer chromatography
RP HPLC: reverse phase high-performance liquid chromatography
