# Supplementary material for: FAP-Inhibitor Homodimers with Engineered Linker-Spacer Units: Toward Optimized Radiopharmaceuticals
Source: ACS Omega. 2026 Aug 11;11(33):49001–19. doi: 10.1021/acsomega.5c13192 (PMC13520045; doi:10.1021/acsomega.5c13192)
Supplement: Supplementary file 1 [file ao5c13192_si_001.pdf]

Supplemental Data

FAP-inhibitor Homodimers with Engineered Linker-Spacer Units: Toward Optimized Radiopharmaceuticals

Naveen Kumar[1#], Marcel Martin[2#], Adrianna Bilinska[1,3], Elena Menéndez[1], Ingrid De Meester[4], Frank Roesch[2], Axel Rominger[1], Eleni Gourni[1]

[1]*Department of Nuclear Medicine, Inselspital, Bern University Hospital, University of Bern, Bern, Switzerland, 3010*

[2]*Department of Chemistry – TRIGA site, Johannes-Gutenberg University, Mainz,Germany, 55128*

[3]*Graduate School of Cellular and Biomedical Sciences, University of Bern, Bern, Switzerland, 3010*

[4]*Department of Pharmaceutical Sciences, Laboratory of Medical Biochemistry, University of Antwerp, Belgium, 2610*

***Corresponding author:**

Eleni Gourni

Department of Nuclear Medicine

Bern University Hospital, Switzerland

Rosenbühlgasse 25, 3010 Bern, Switzerland.

Email: eleni.gourni@insel.ch

Tel.: +41 (0)31 664 0507.

Content

S.No.	Description	Page no.
1.	Organic synthesis - analytical RP-HPLC	S2
2.	Radiosynthesis - Radio-HPLCs before and after purification	S5
3.	Radiosynthesis – complex stabilities	S16
4.	In vitro-inhibition assays	S17
5.	Experimental Section	S19

Organic synthesis - analytical RP-HPLC

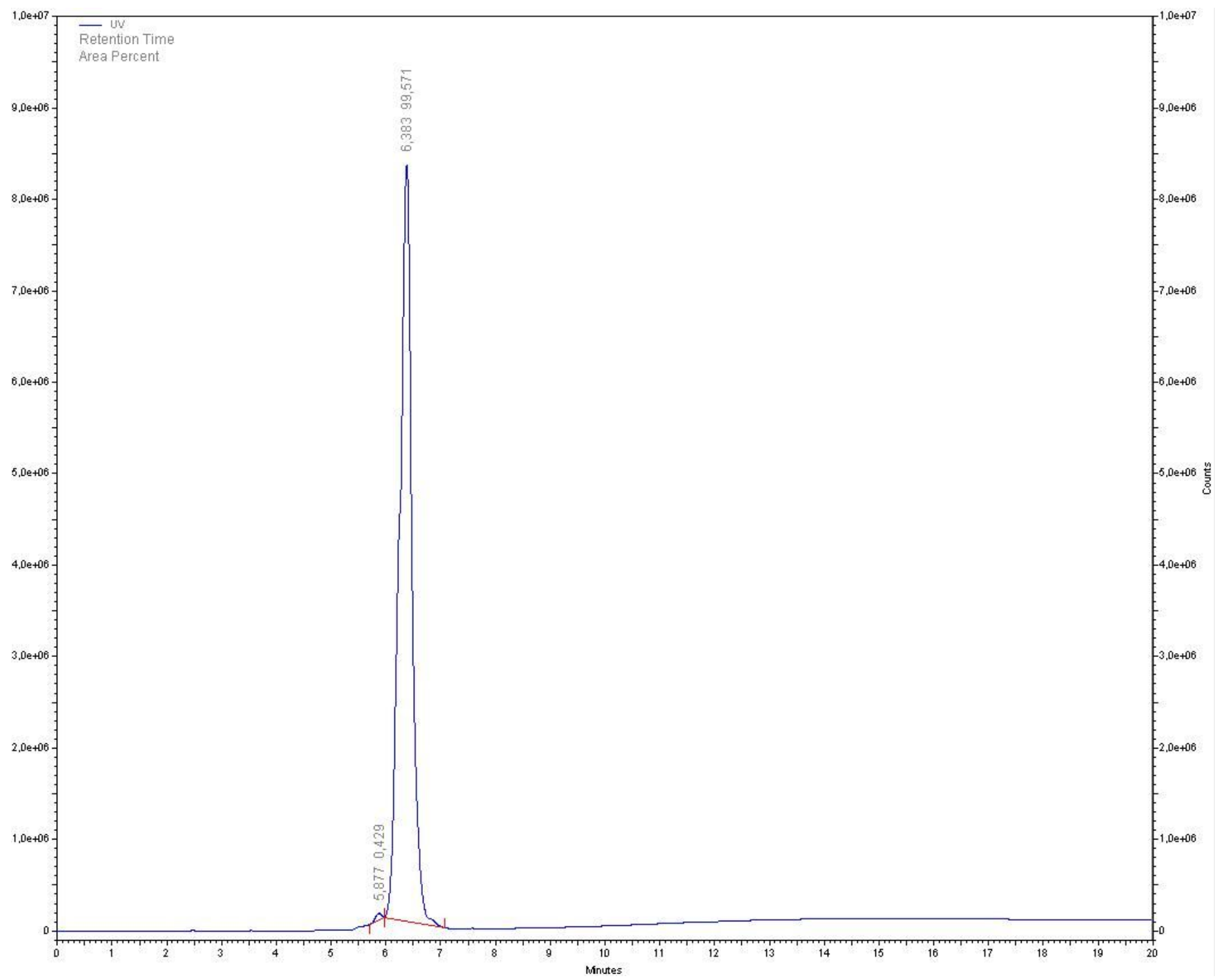

Figure S1: Analytical RP-HPLC of DOTAGA.NPyr.$(FAPi)_2$ **8** (t_R = 6.4 min), 30-40% B in 20 min, 5 mL/min: purity = 99.6%.

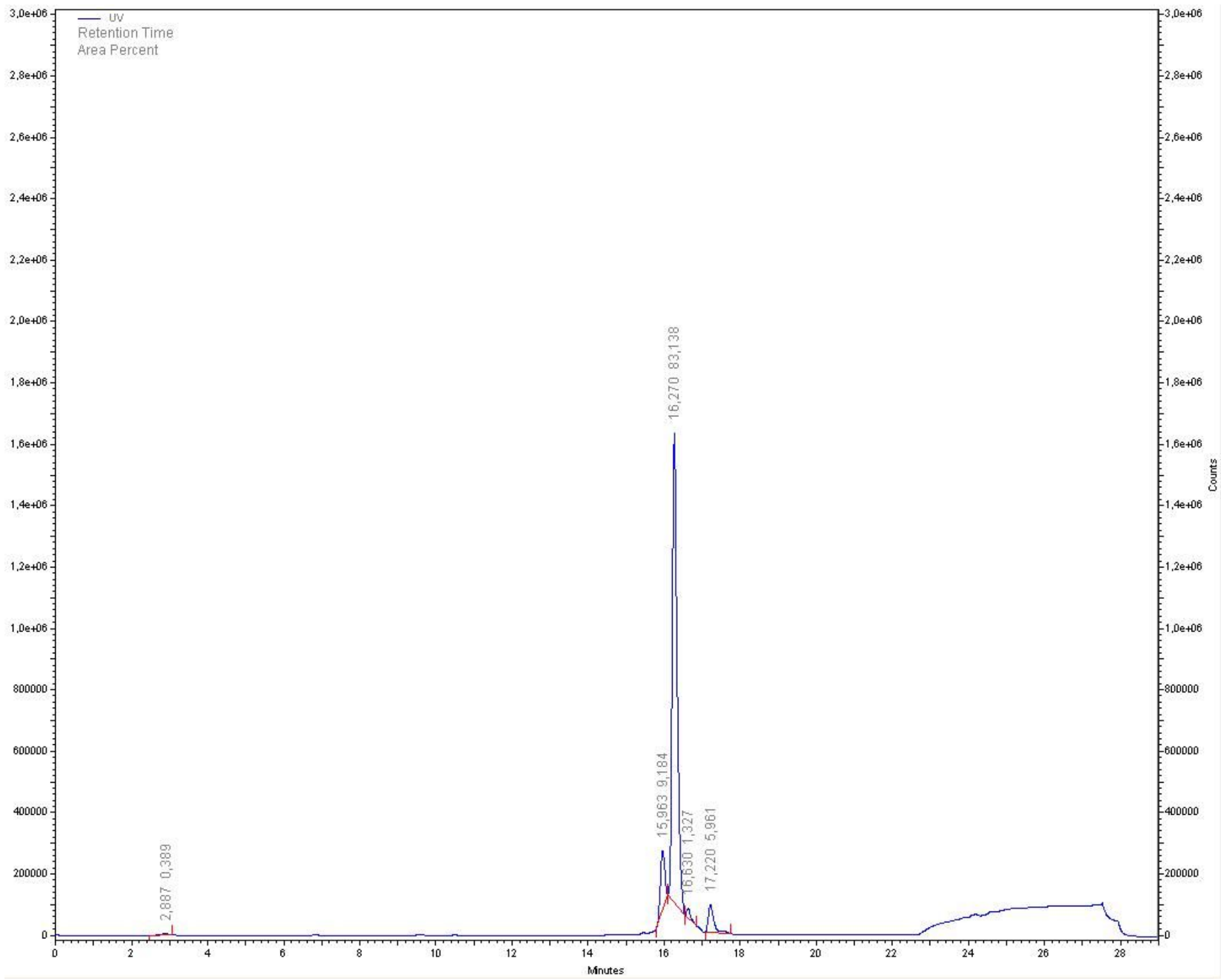

Figure S2: Analytical RP-HPLC of DO3A.NPyr.(FAPi)$_2$ **11** (t_R = 16.3 min), 25-35% B in 20 min, 5 mL/min: purity = 95.2%.

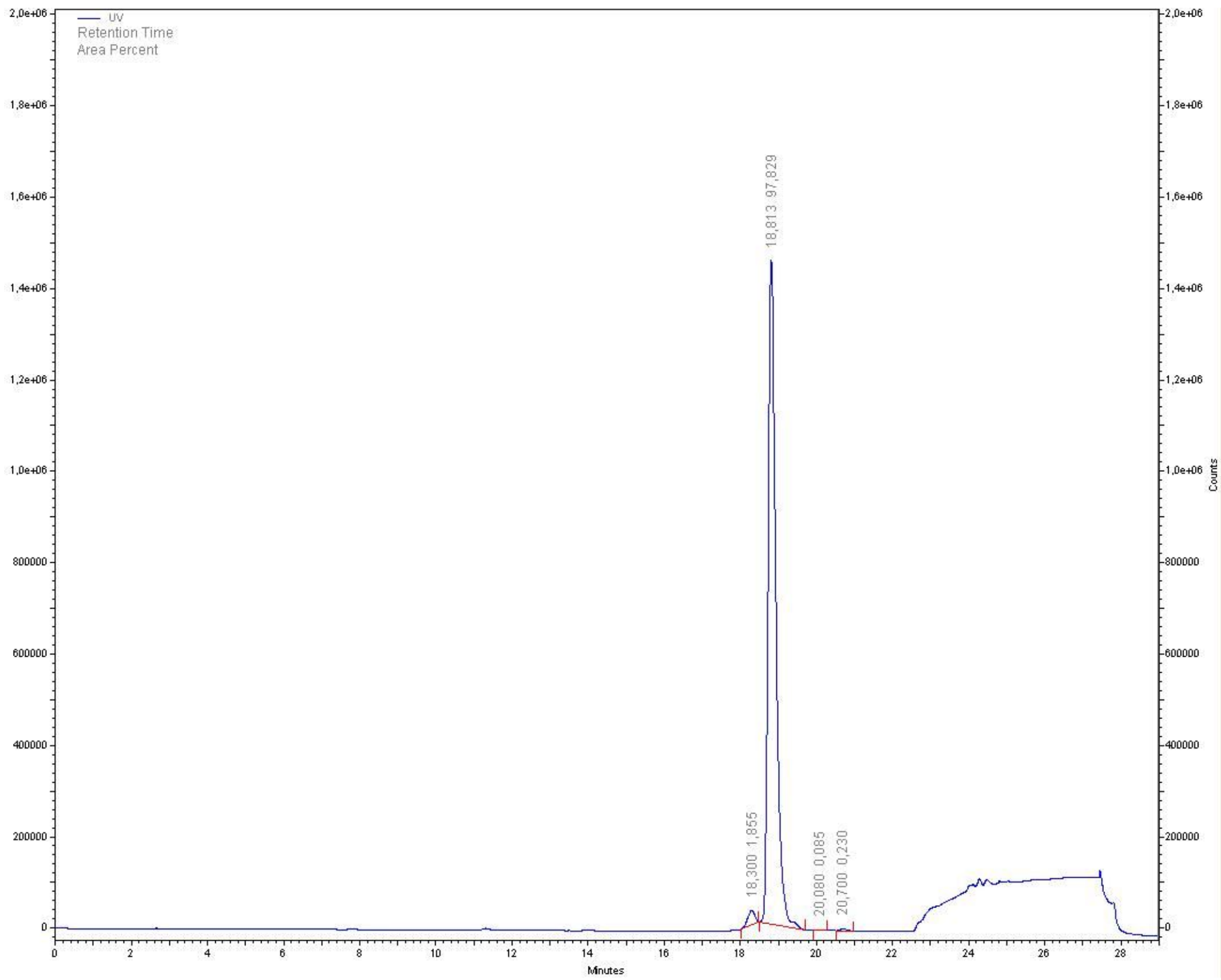

Figure S3: Analytical RP-HPLC of DOTAGA.(Glu)$_2$.(FAPi)$_2$ **18** (t_R = 18.8 min), 20-30% B in 20 min, 5 mL/min: purity = 97.8%.

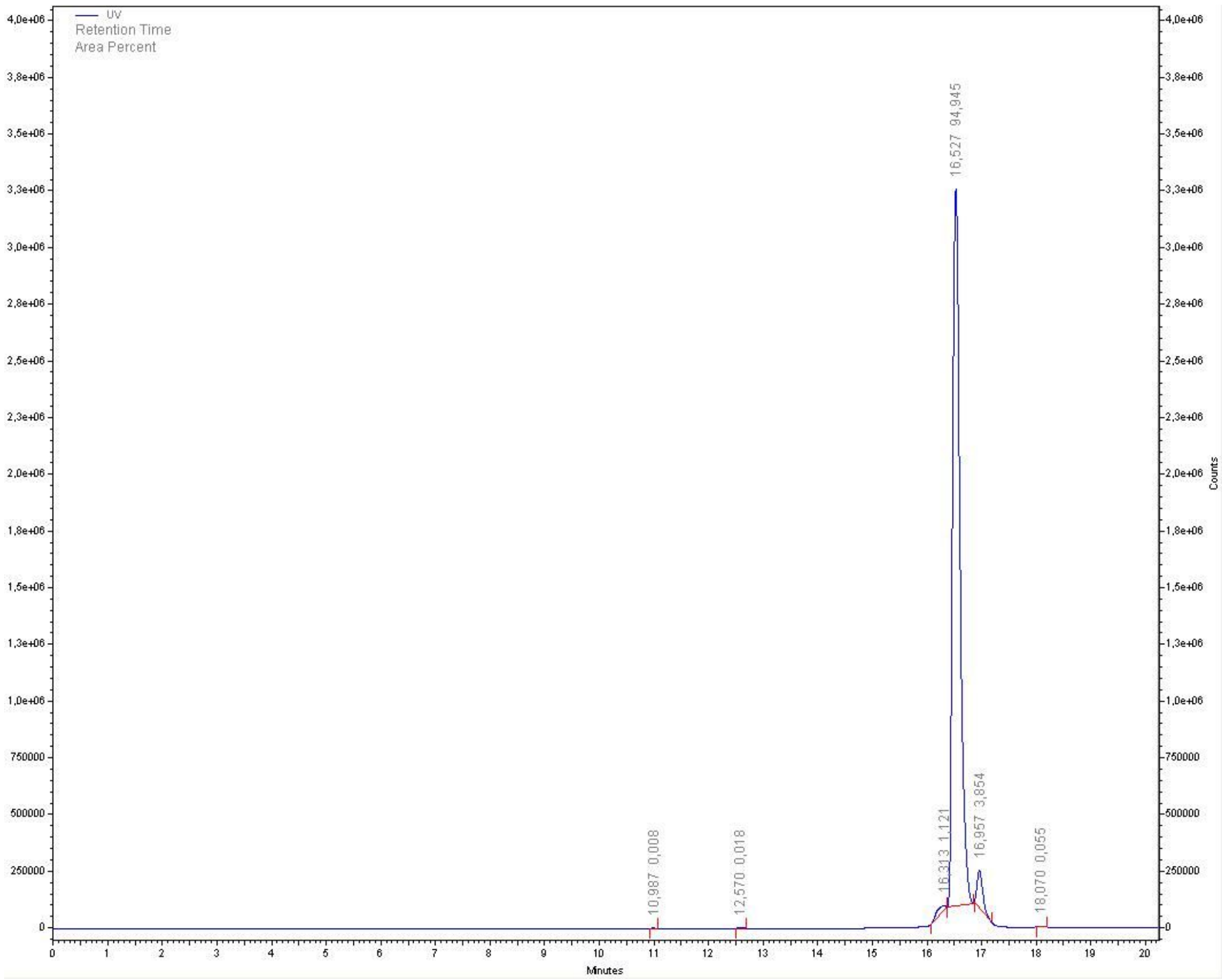

Figure S4: Analytical RP-HPLC of DO3A.(Glu)$_2$.(FAPi)$_2$ **19** (t_R = 16.5 min), 25-35% B in 20 min, 5 mL/min: purity = 95.9%.

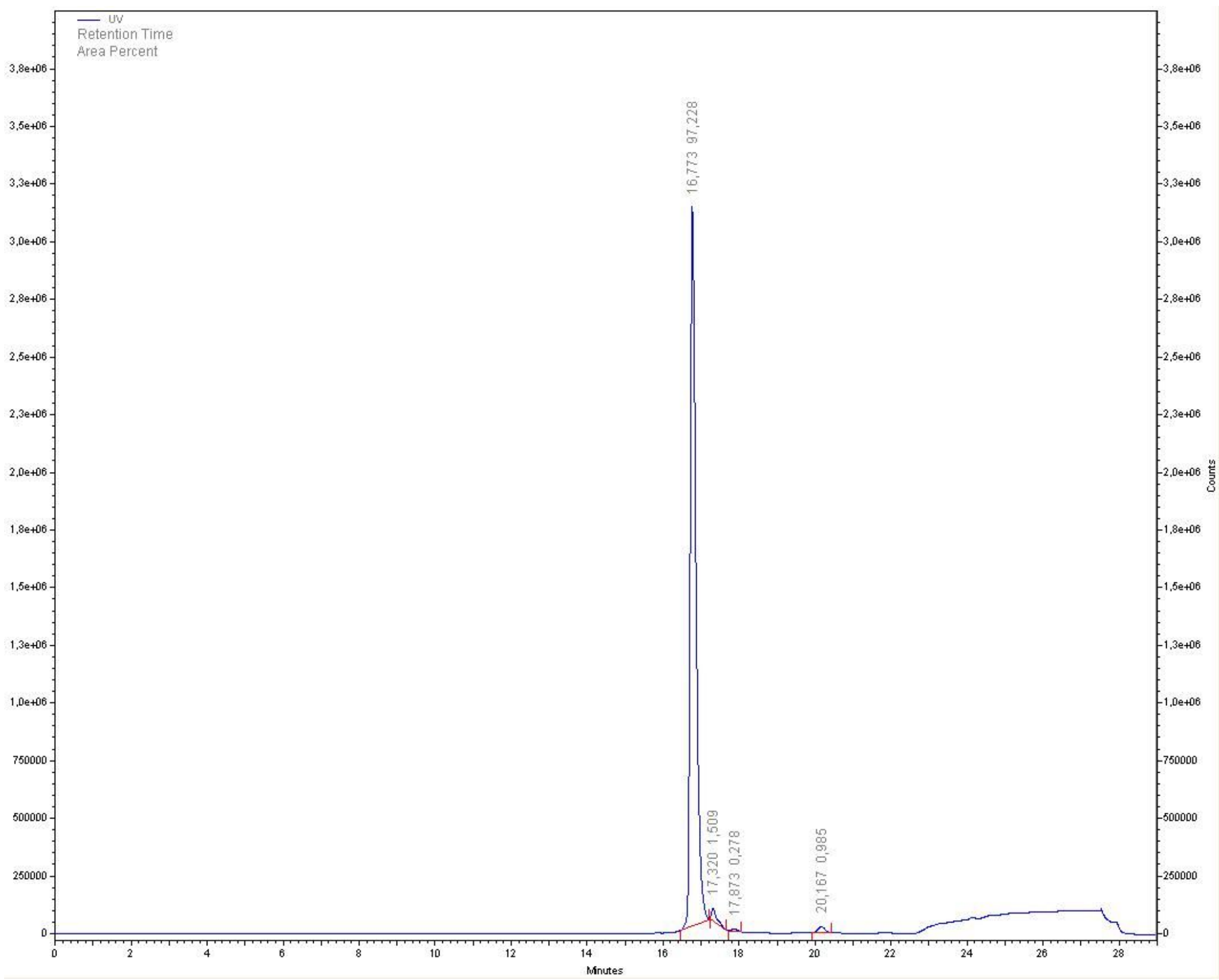

Figure S5: Analytical RP-HPLC of DOTAGA.PEG$_2$.Glu.(FAPi)$_2$ **25** (t_R = 16.8 min), 25-35% B in 20 min, 5 mL/min: purity = 97.2%.

Radiosynthesis - Radio-HPLCs Before and After Purification

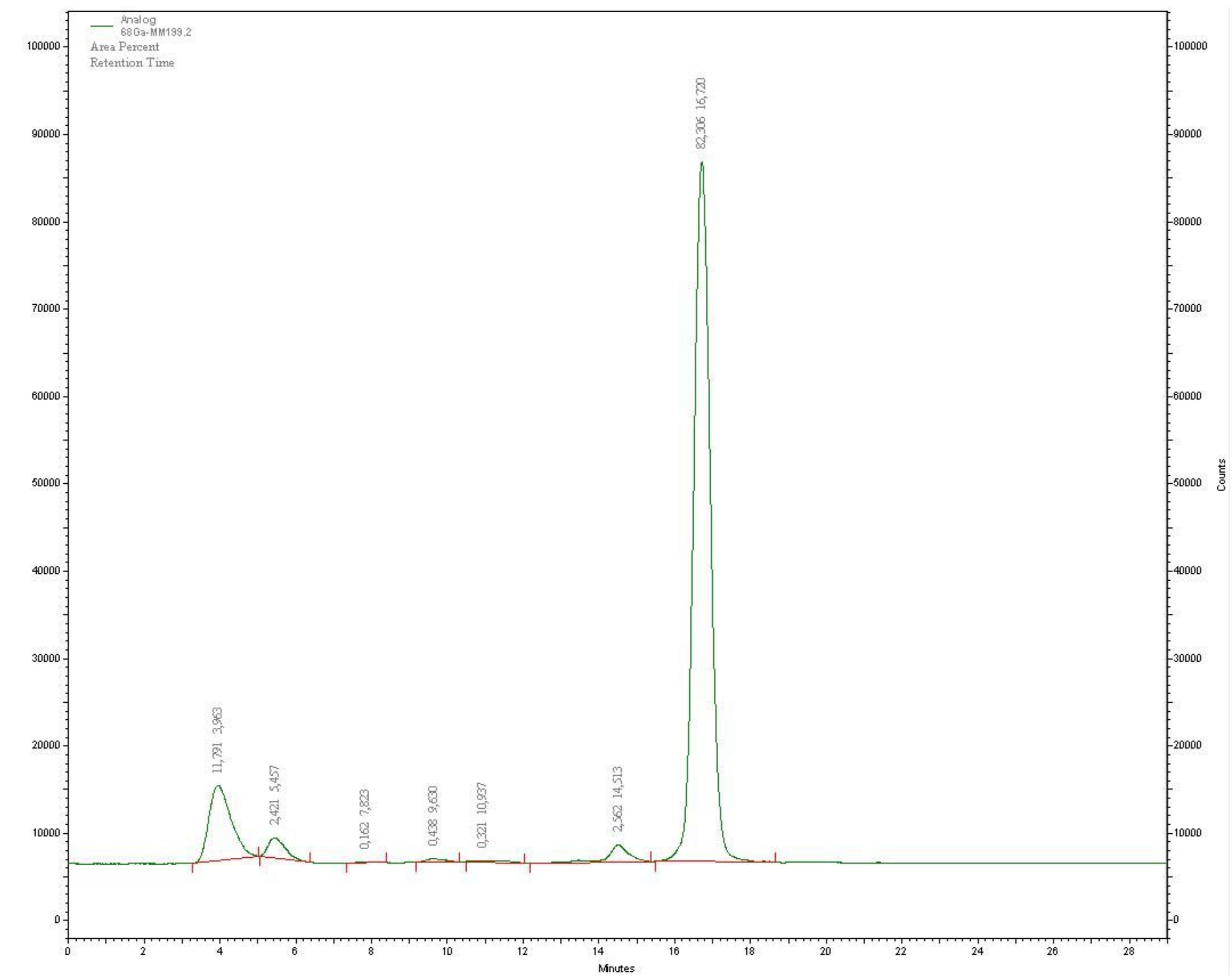

Figure S6: Analytical Radio-HPLC of [^{68}Ga]Ga-DOTAGA.NPyr.(FAPi)$_2$ **^{68}Ga-8** (t_R = 16.7 min) after radiolabeling with 10 nmol **8**, and 50 MBq ^{68}Ga in 1 M HEPES (pH = 5.5), 20-40% MeCN + 0.1% TFA (linear gradient) in 20 min, 1 mL/min.

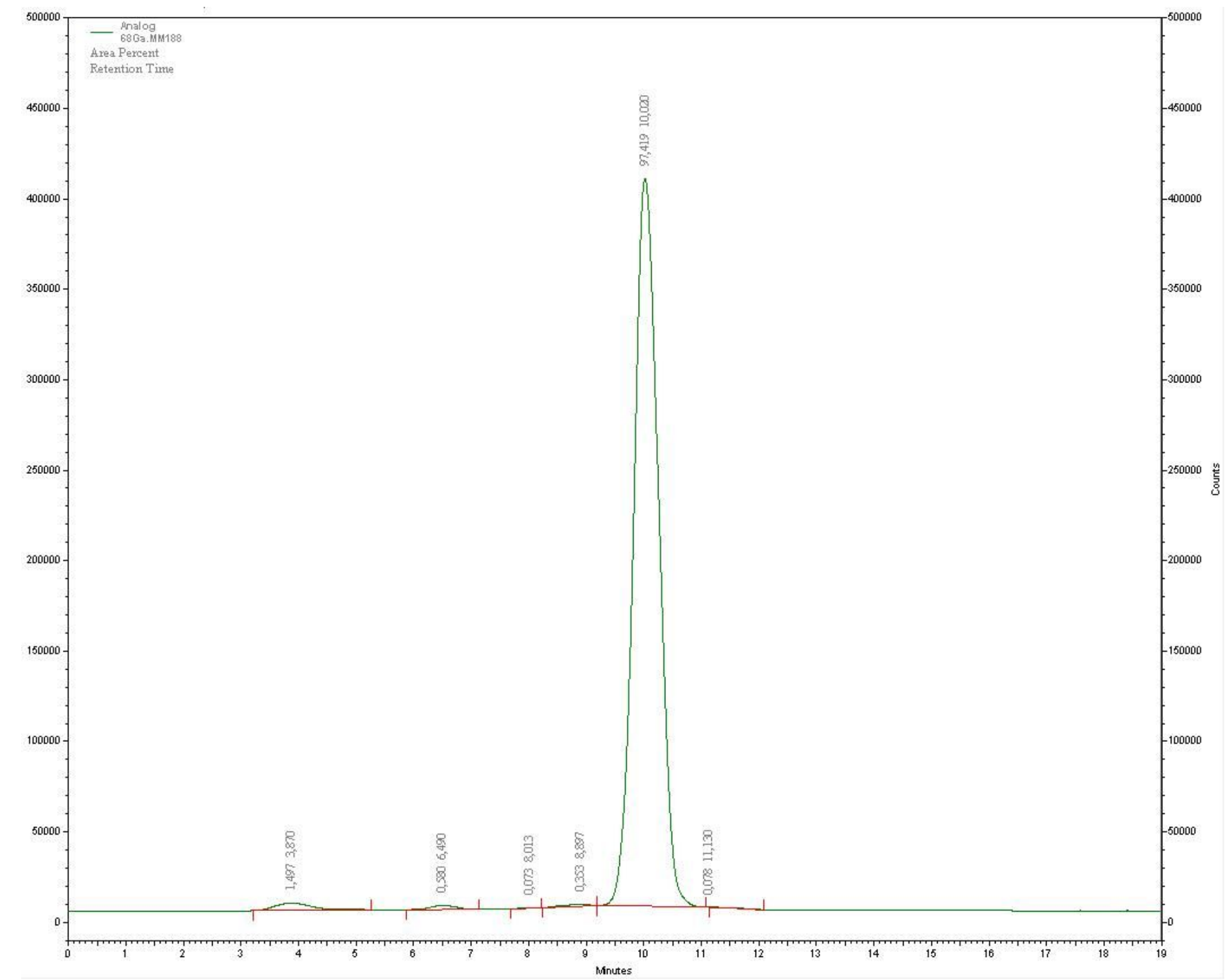

Figure S7: Analytical Radio-HPLC of [^{68}Ga]Ga-DOTAGA.NPyr.(FAPi)$_2$ **^{68}Ga-8** (t_R = 10.0 min) after C18 cartridge purification, 20-50% MeCN + 0.1% TFA (linear gradient) in 20 min, 1 mL/min.

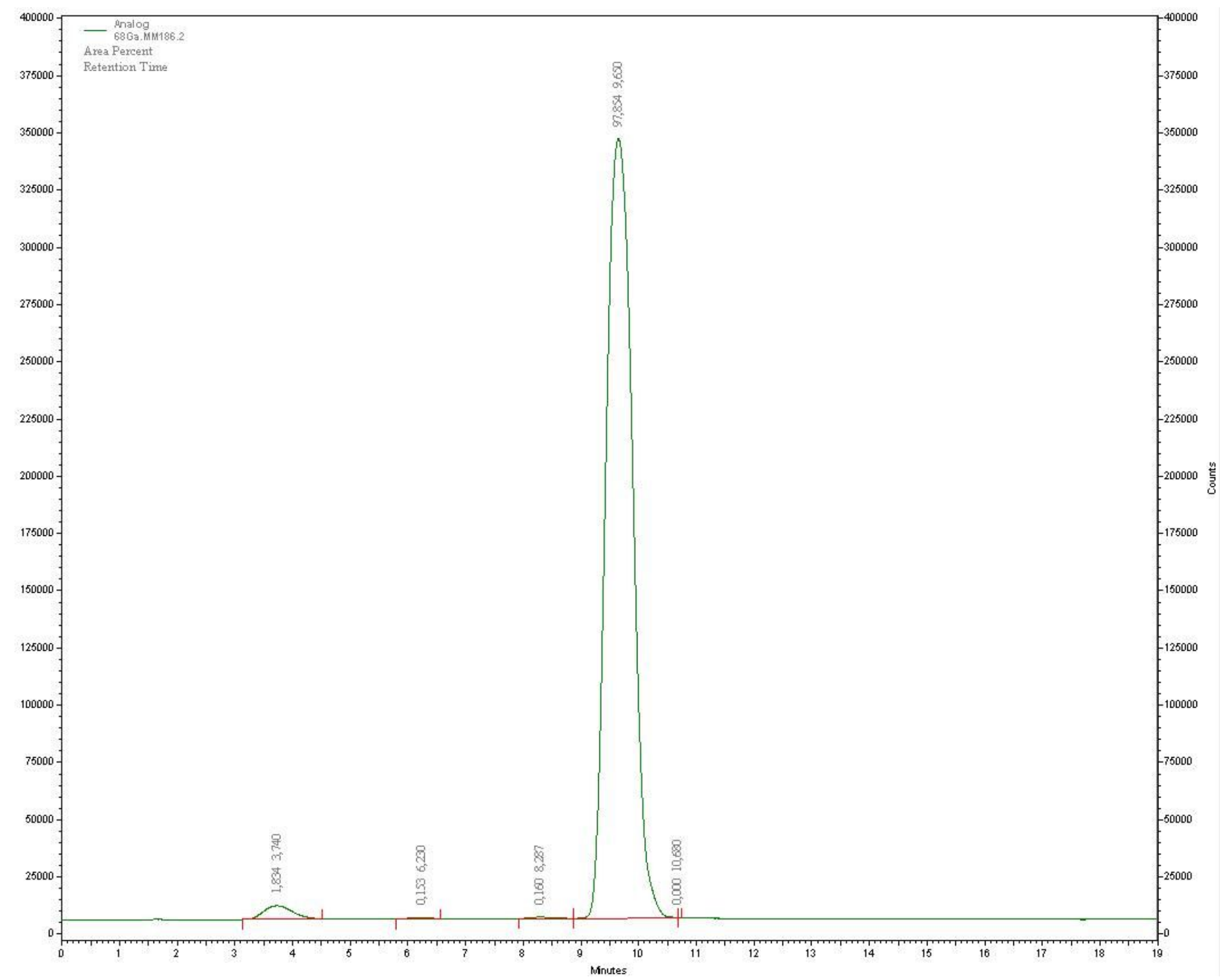

Figure S8: Analytical Radio-HPLC of [^{68}Ga]Ga-DO3A.NPyr.(FAPi)$_2$ **^{68}Ga-11** (t_R = 9.7 min) after radiolabeling with 10 nmol **11**, and 50 MBq ^{68}Ga in 1 M HEPES (pH = 4.5), 20-50% MeCN + 0.1% TFA (linear gradient) in 20 min, 1 mL/min.

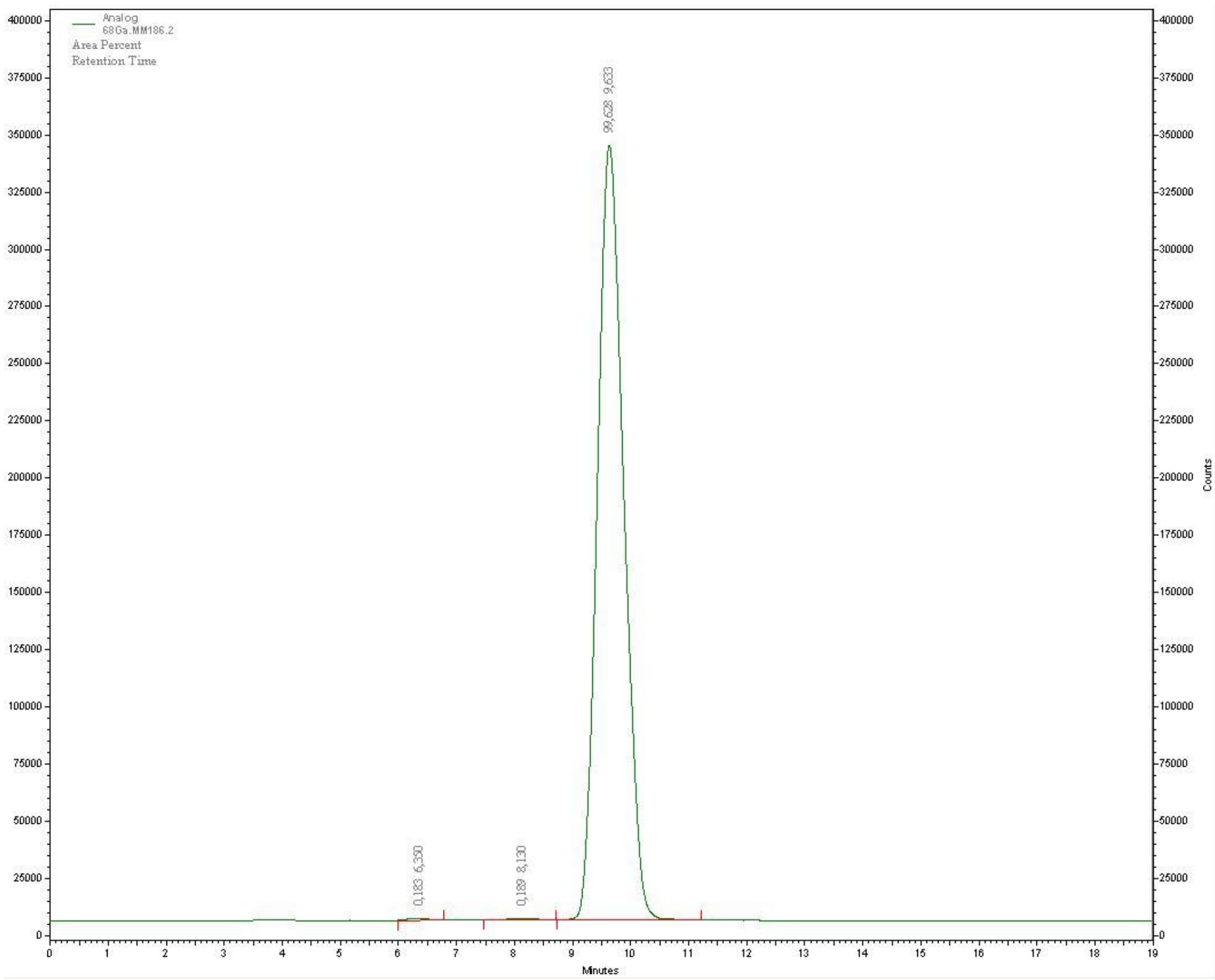

Figure S9: Analytical Radio-HPLC of [^{68}Ga]Ga-DO3A.NPyr.(FAPi)$_2$ **^{68}Ga-11** (t_R = 9.7 min) after C18 cartridge purification, 20-50% MeCN + 0.1% TFA (linear gradient) in 20 min, 1 mL/min.

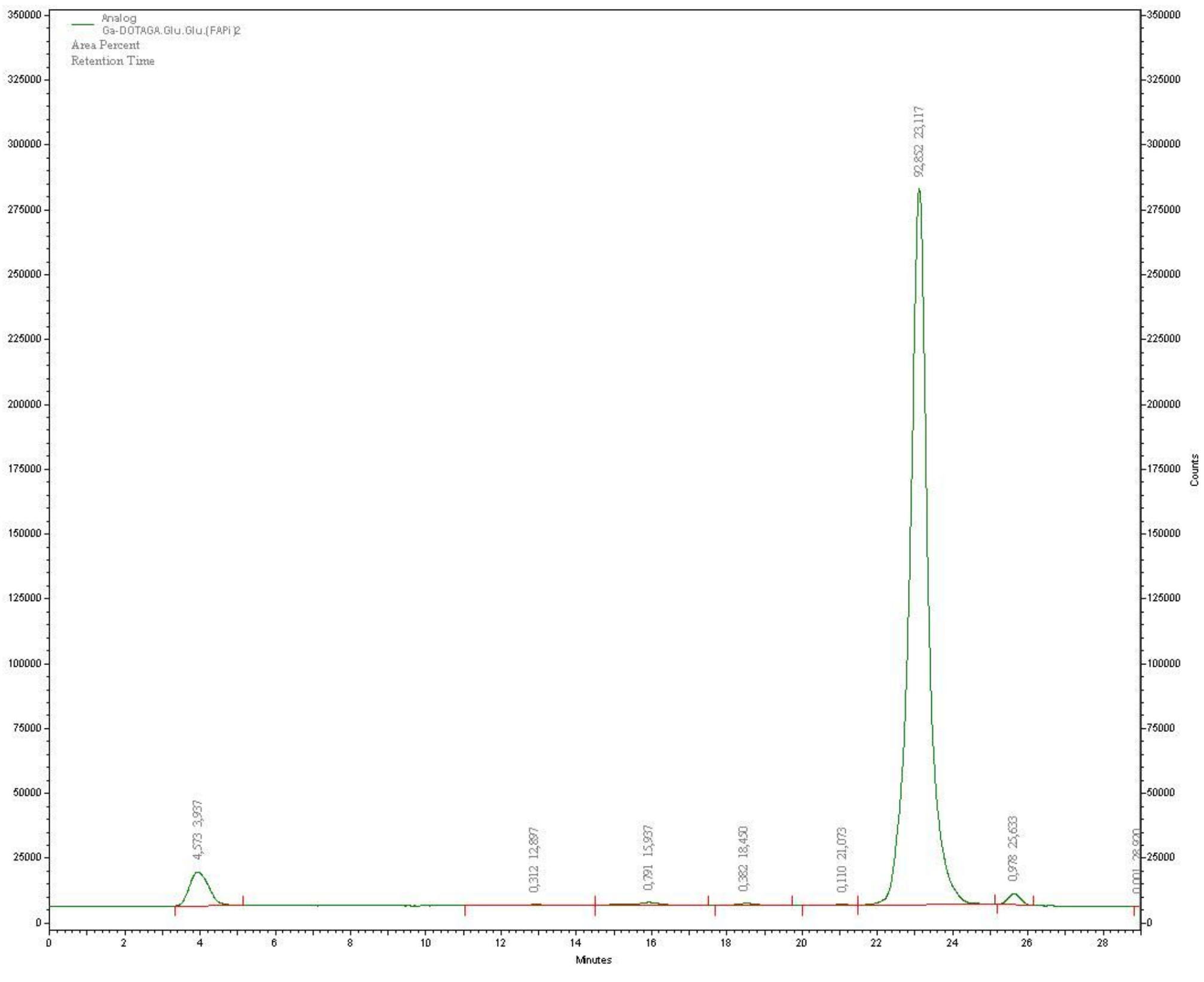

Figure S10: Analytical Radio-HPLC of [^{68}Ga]Ga-DOTAGA.(Glu)$_2$.(FAPi)$_2$ **^{68}Ga-18** (t_R = 23.1 min) after radiolabeling with 10 nmol **18**, and 50 MBq ^{68}Ga in 1 M HEPES (pH = 5.5), 20-30% MeCN + 0.1% TFA (linear gradient) in 30 min, 1 mL/min.

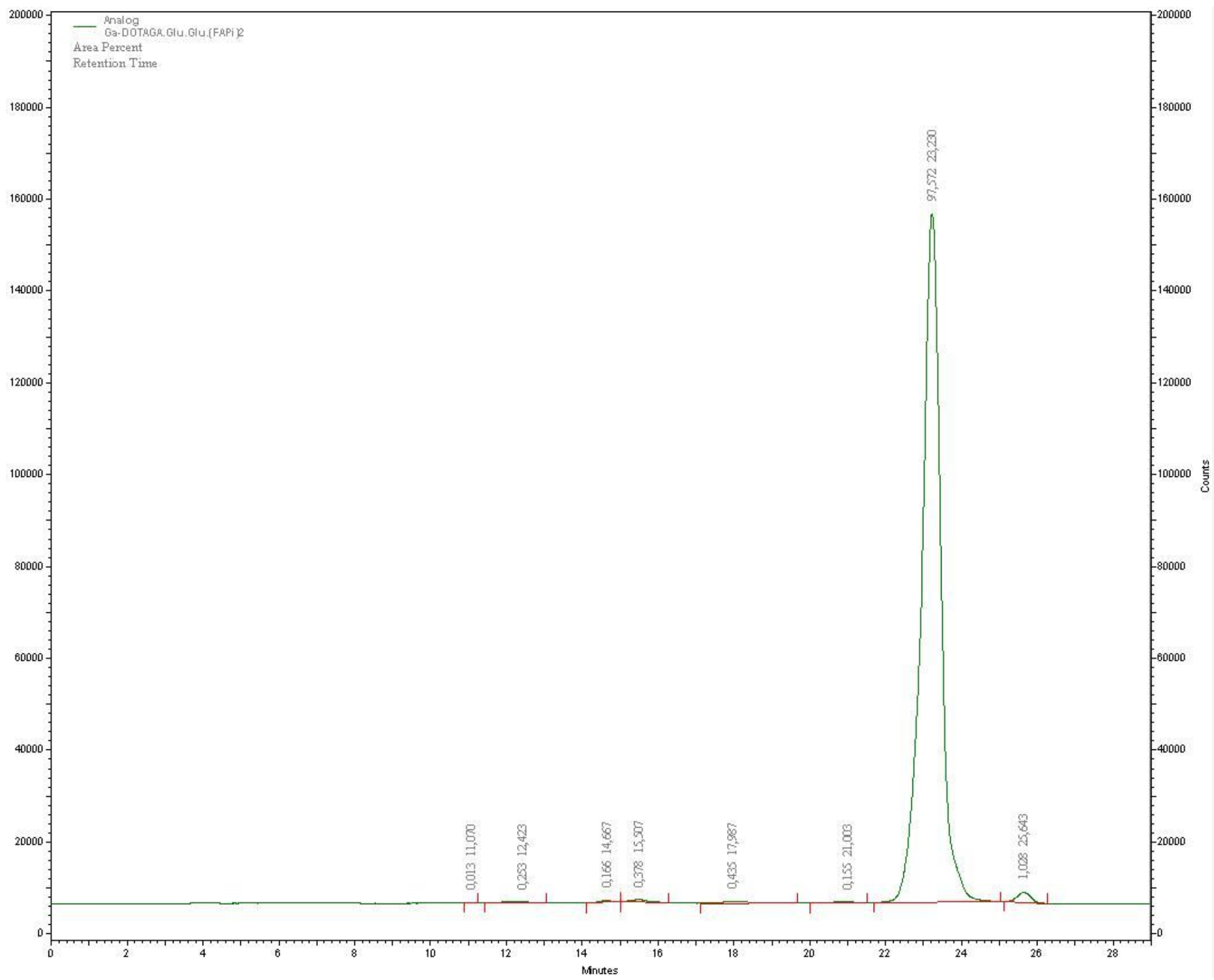

Figure S11: Analytical Radio-HPLC of [^{68}Ga]Ga-DOTAGA.(Glu)$_2$.(FAPi)$_2$ **^{68}Ga-18** (t_R = 23.2 min) after C18 cartridge purification, 20-30% MeCN + 0.1% TFA (linear gradient) in 30 min, 1 mL/min.

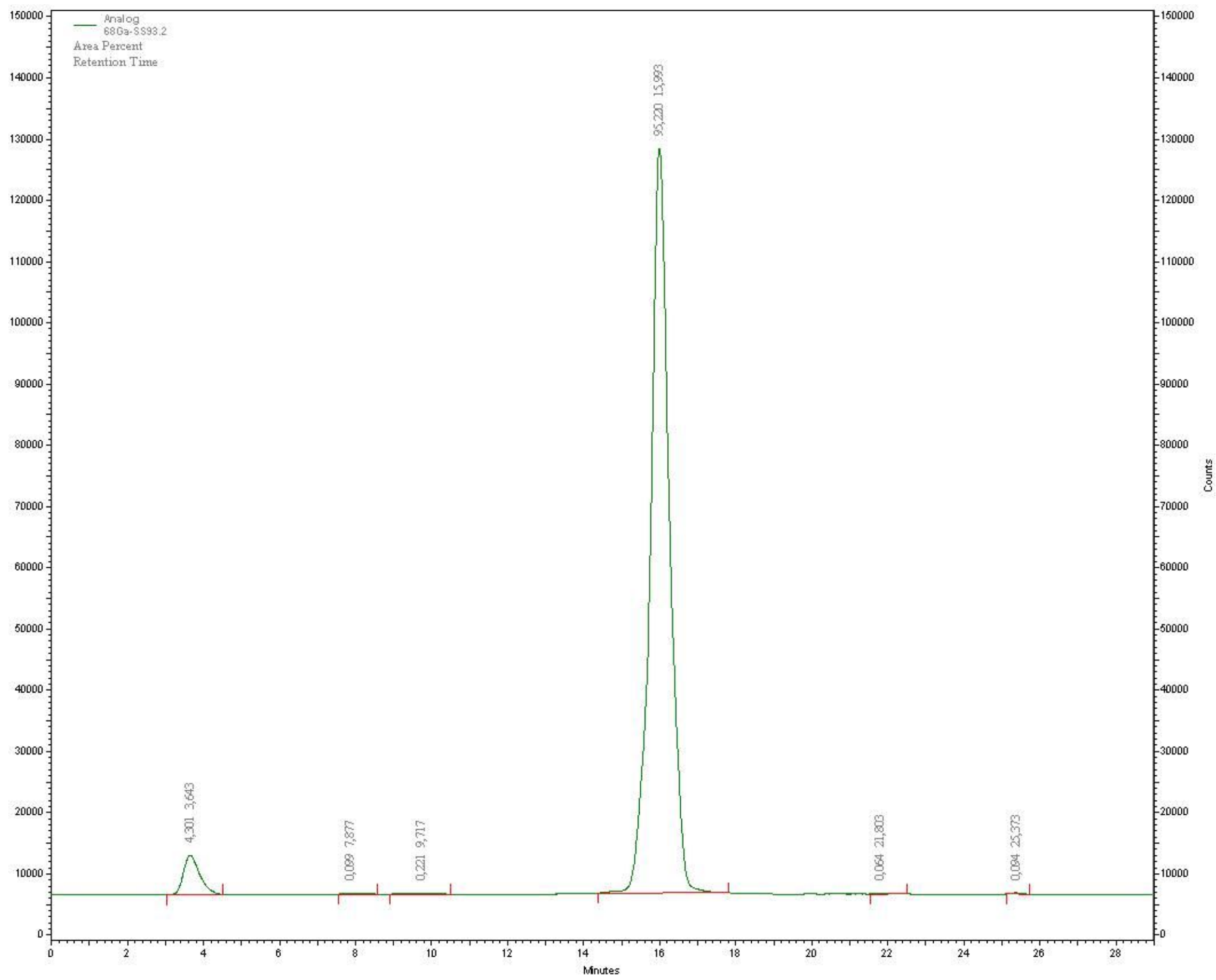

Figure S12: Analytical Radio-HPLC of [^{68}Ga]Ga-DO3A.(Glu)$_2$.(FAPi)$_2$ **^{68}Ga-19** (t_R = 16.0 min) after radiolabeling with 10 nmol **19**, and 50 MBq ^{68}Ga in 1 M HEPES (pH = 4.5), 20-50% MeCN + 0.1% TFA (linear gradient) in 30 min, 1 mL/min.

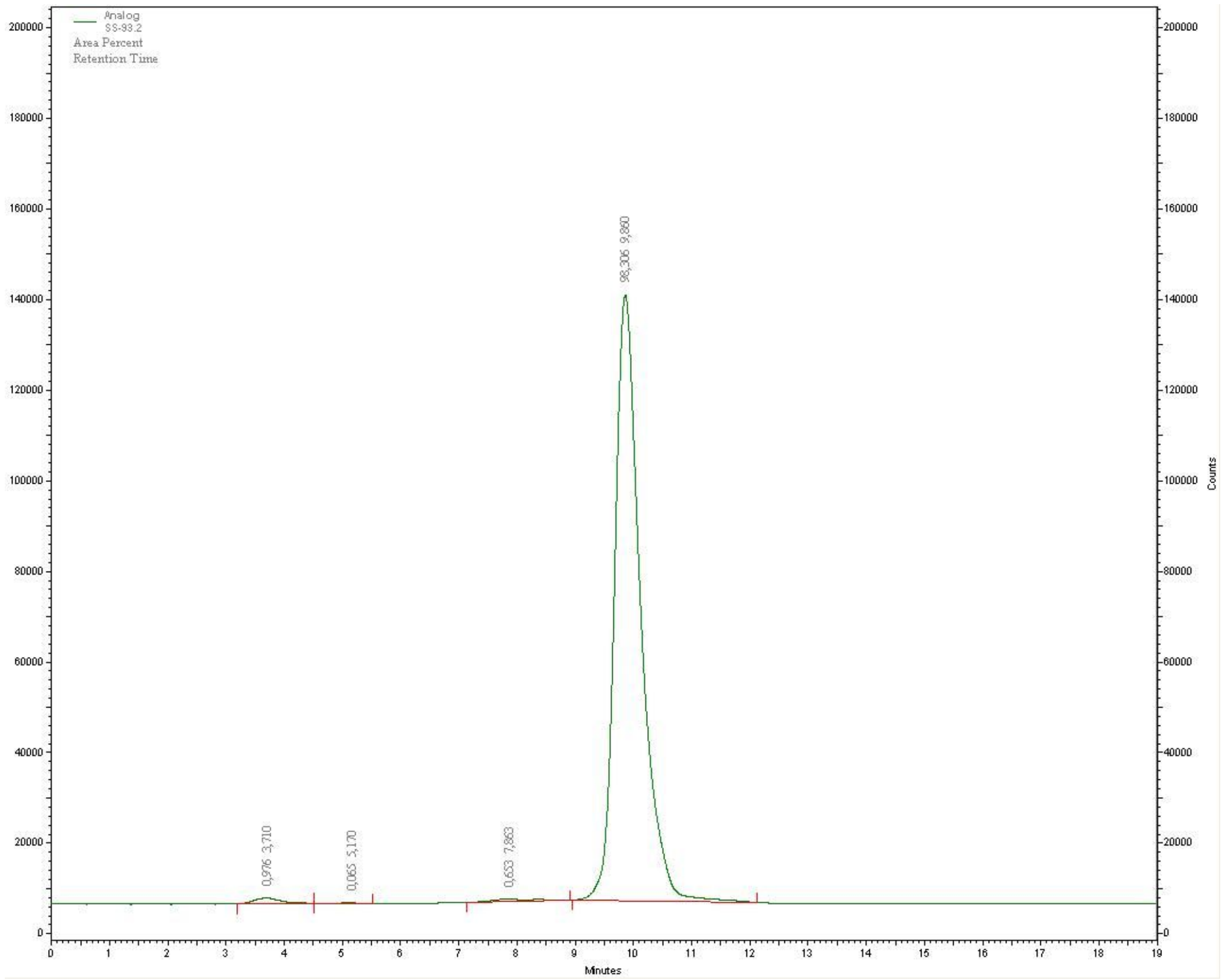

Figure S13: Analytical Radio-HPLC of [^{68}Ga]Ga-DO3A.(Glu)$_2$.(FAPi)$_2$ **^{68}Ga-19** (t_R = 9.9 min) after C18 cartridge purification, 20-50% MeCN + 0.1% TFA (linear gradient) in 20 min, 1 mL/min.

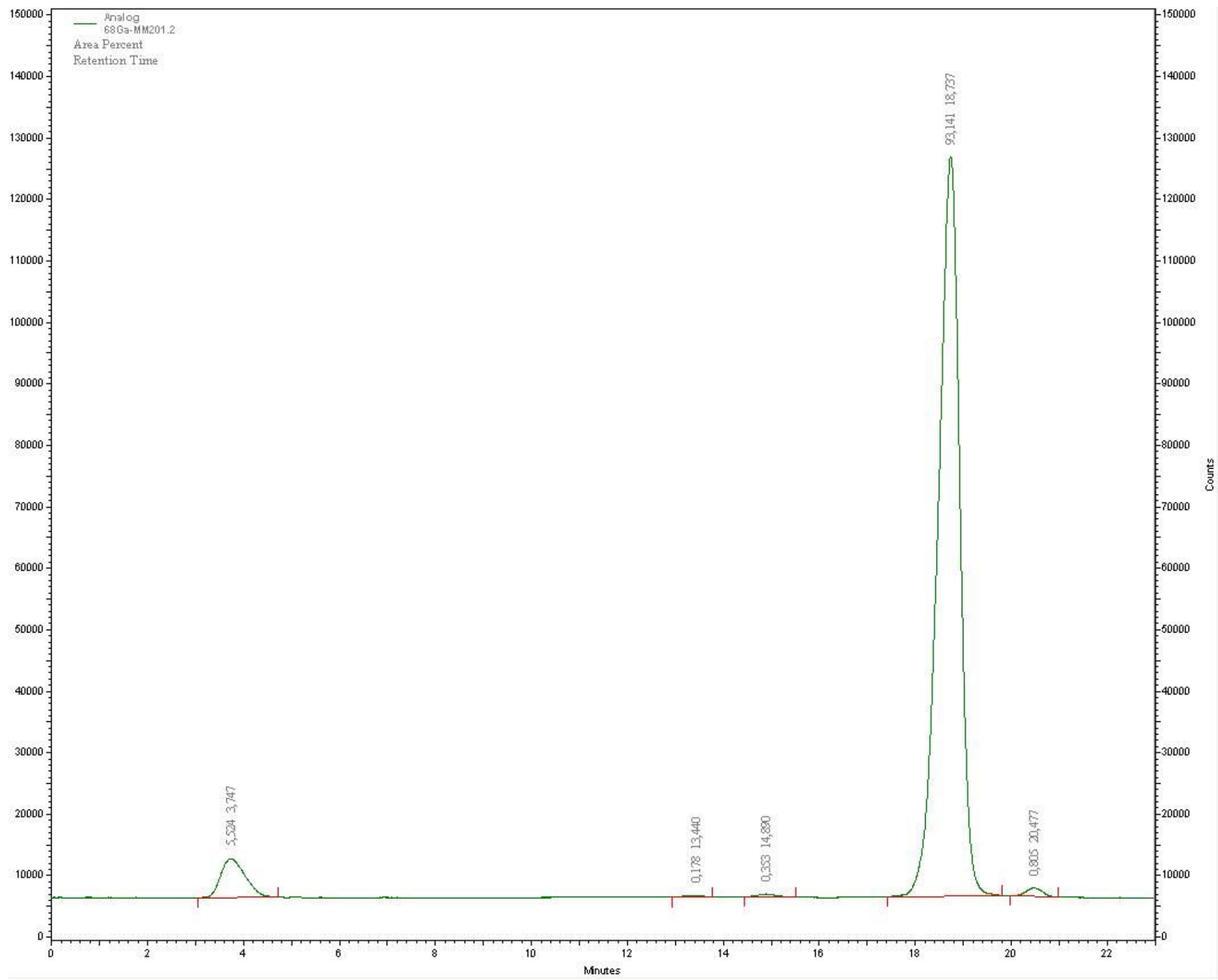

Figure S14: Analytical Radio-HPLC of [^{68}Ga]Ga-DOTAGA.PEG$_2$.Glu.(FAPi)$_2$ **^{68}Ga-25** (t_R = 18.7 min) after radiolabeling with 10 nmol **25**, and 50 MBq ^{68}Ga in 1 M AmOAc (pH = 4.5), 20-30% MeCN + 0.1% TFA (linear gradient) in 30 min, 1 mL/min.

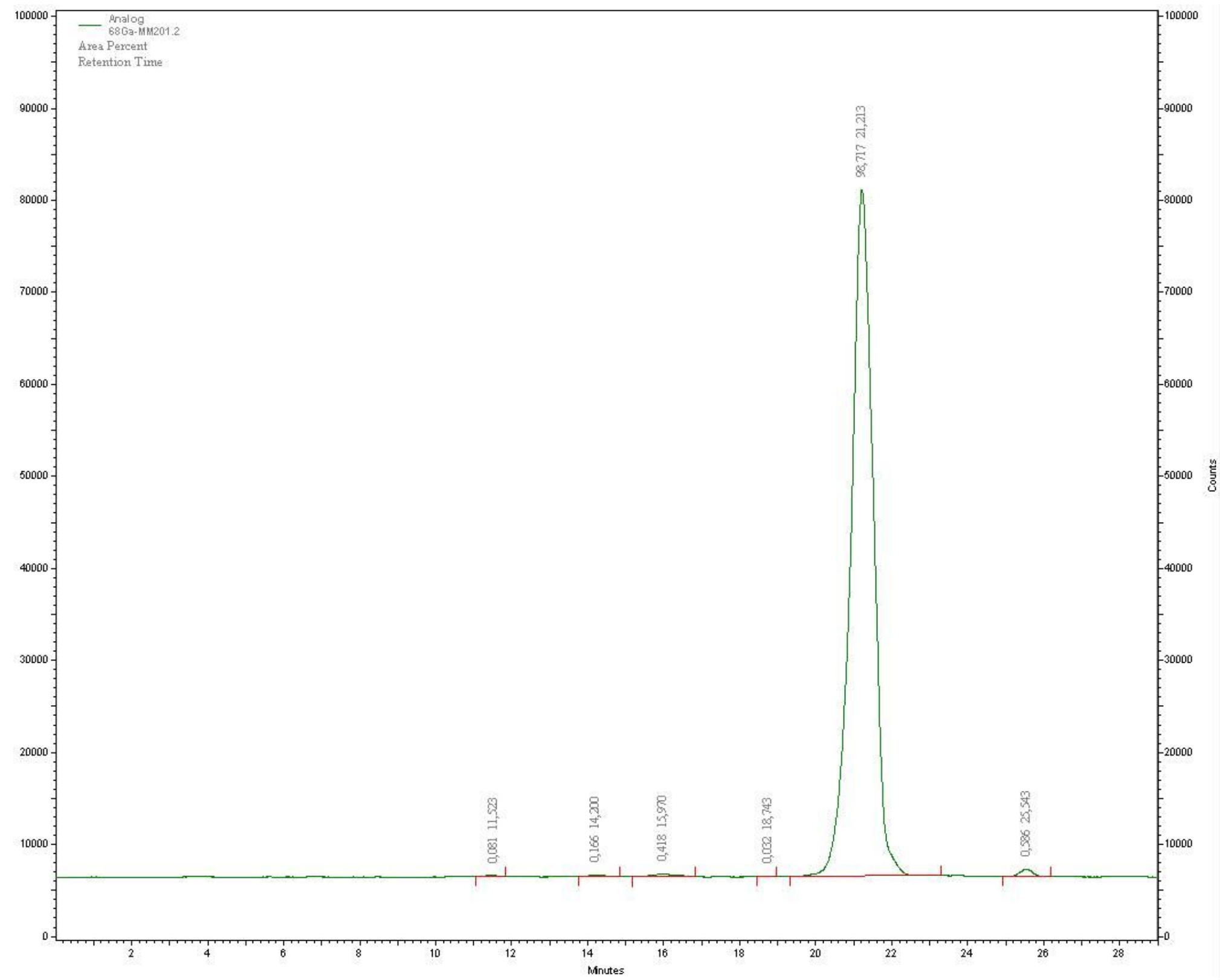

Figure S15: Analytical Radio-HPLC of [^{68}Ga]Ga-DOTAGA.PEG$_2$.Glu.(FAPi)$_2$ **^{68}Ga-25** (t_R = 21.2 min) after C18 cartridge purification, 20-30% MeCN + 0.1% TFA (linear gradient) in 30 min, 1 mL/min.

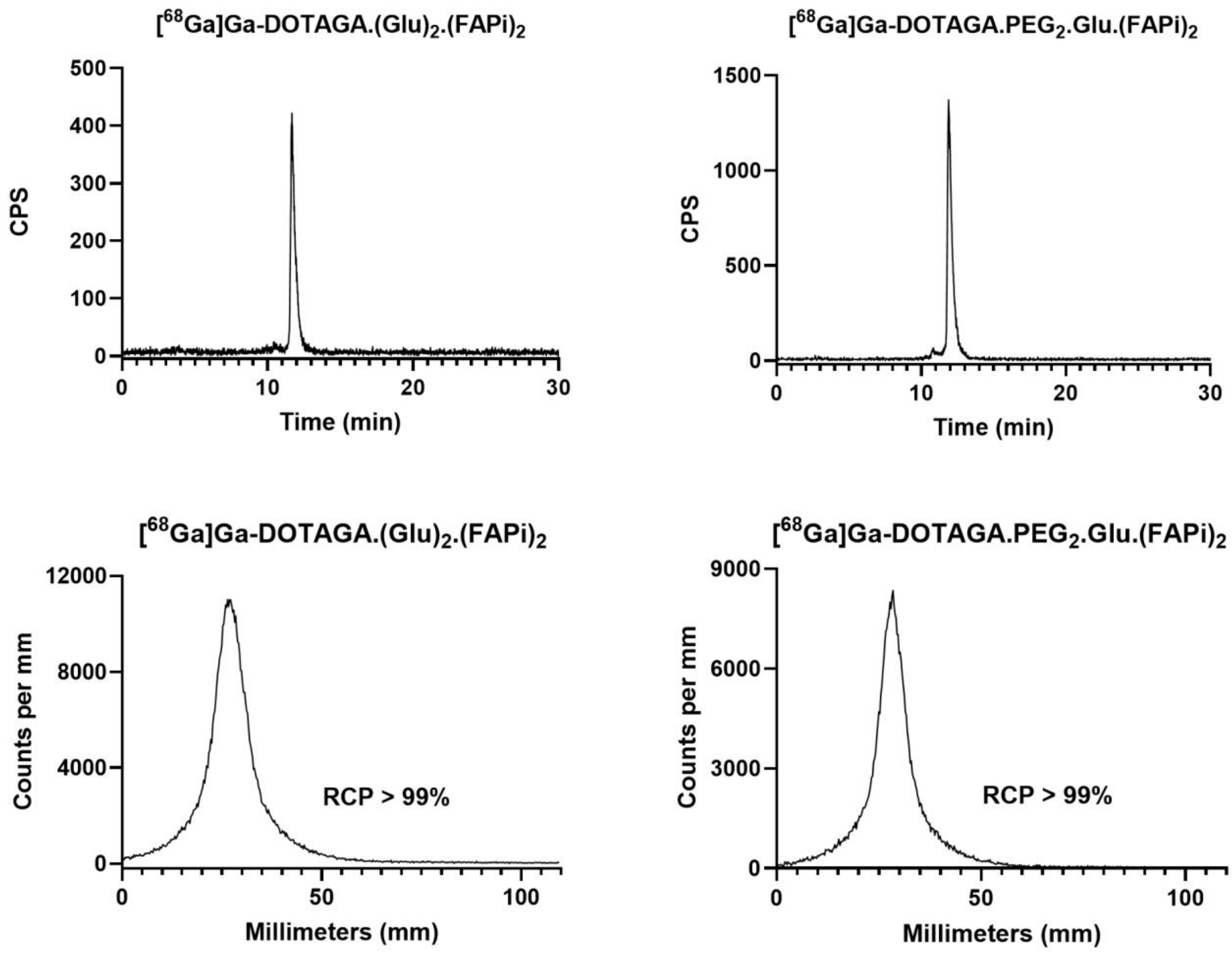

Figure S16: Analytical Radio-HPLC and TLC of $[^{68}Ga]Ga$-DOTAGA.$(Glu)_2$.$(FAPi)_2$ **^{68}Ga-18 (**15 nmol) (t_R = 11.4 min) and $[^{68}Ga]Ga$-DOTAGA.PEG_2.Glu.$(FAPi)_2$ **^{68}Ga-25** (15 nmol) (t_R = 11.5 min) after radiolabeling with ^{68}Ga in 1 M HEPES (pH = 5.5). Radio-HPLC analysis with solvent 15-90% B (1 mL/min) in 30 min. Radio-TLC analysis was performed with aluminum coated silica gel 60 F254 plates from Merck as stationary phase and citrate buffer 0.1 M at pH 5 as a mobile phase.

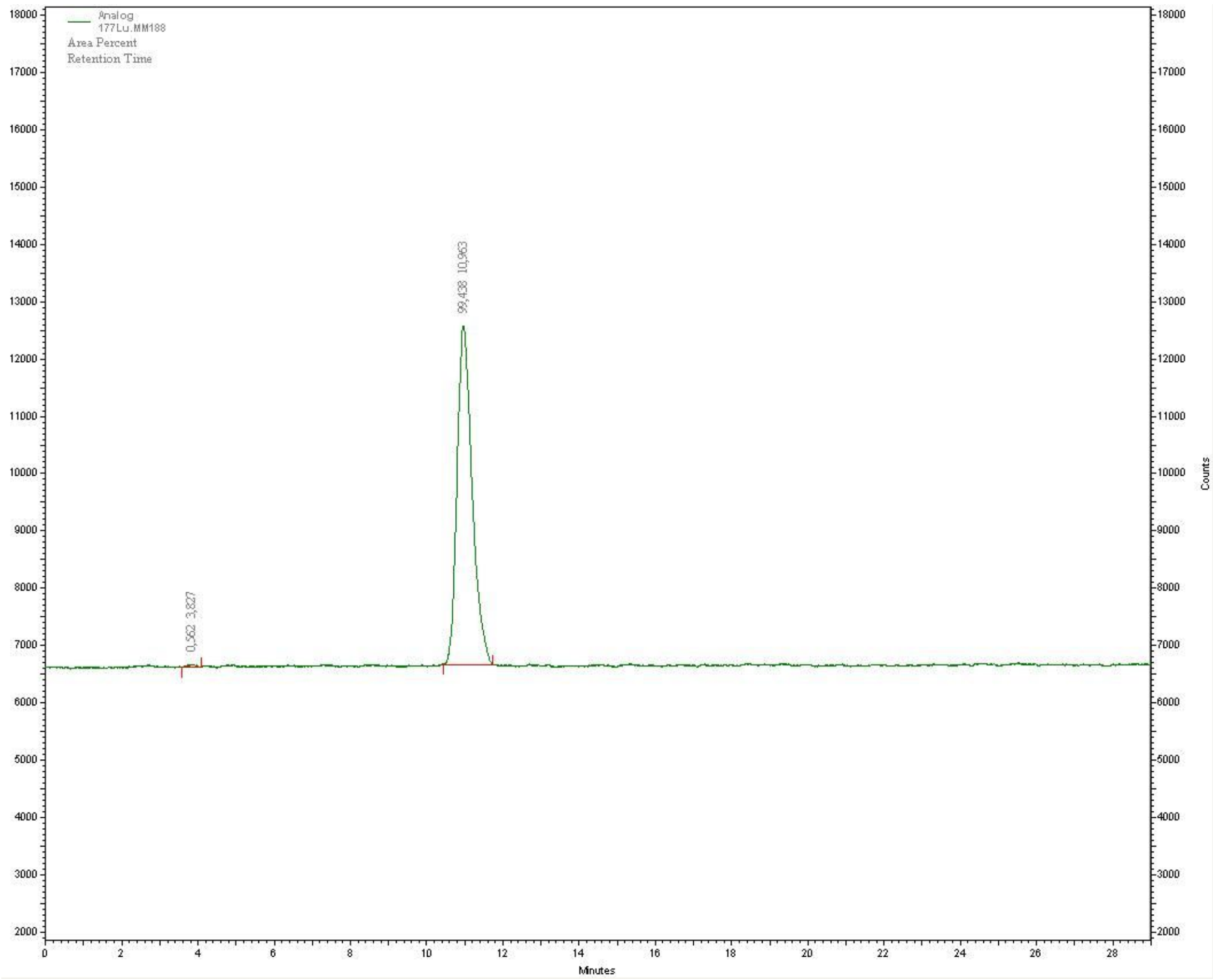

Figure S17: Analytical Radio-HPLC of [^{177}Lu]Lu-DOTAGA.NPyr.(FAPi)$_2$ **^{177}Lu-8** (t_R = 11.0 min) after radiolabeling with 10 nmol **8**, and 50 MBq ^{177}Lu in 1 M HEPES (pH = 5.5), 20-50% MeCN + 0.1% TFA (linear gradient) in 30 min, 1 mL/min.

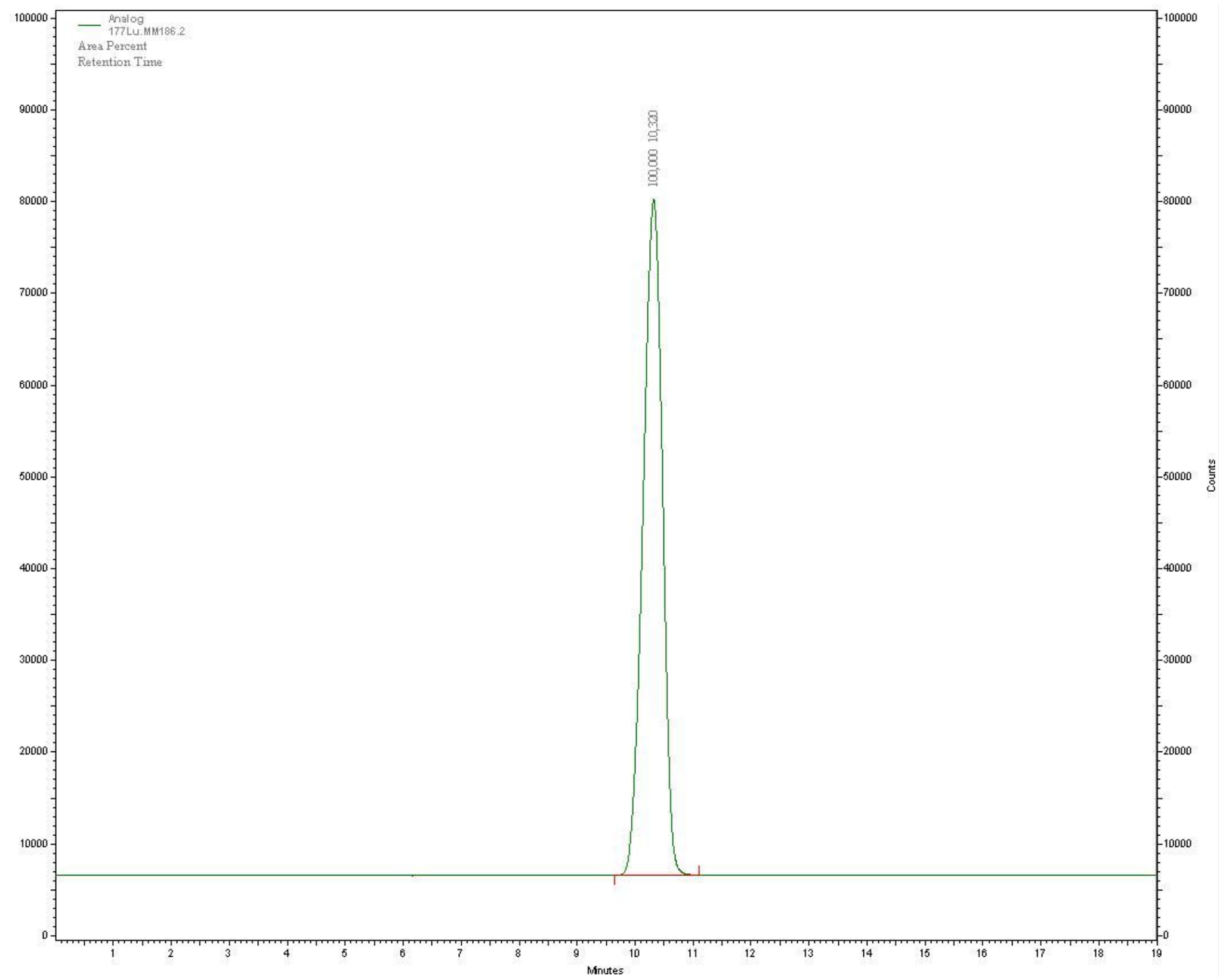

Figure S18: Analytical Radio-HPLC of [^{177}Lu]Lu-DO3A.NPyr.(FAPi)$_2$ **^{177}Lu-11** (t_R = 10.3 min) after radiolabeling with 10 nmol **11**, and 50 MBq ^{177}Lu in 1 M HEPES (pH = 5.5), 20-50% MeCN + 0.1% TFA (linear gradient) in 20 min, 1 mL/min.

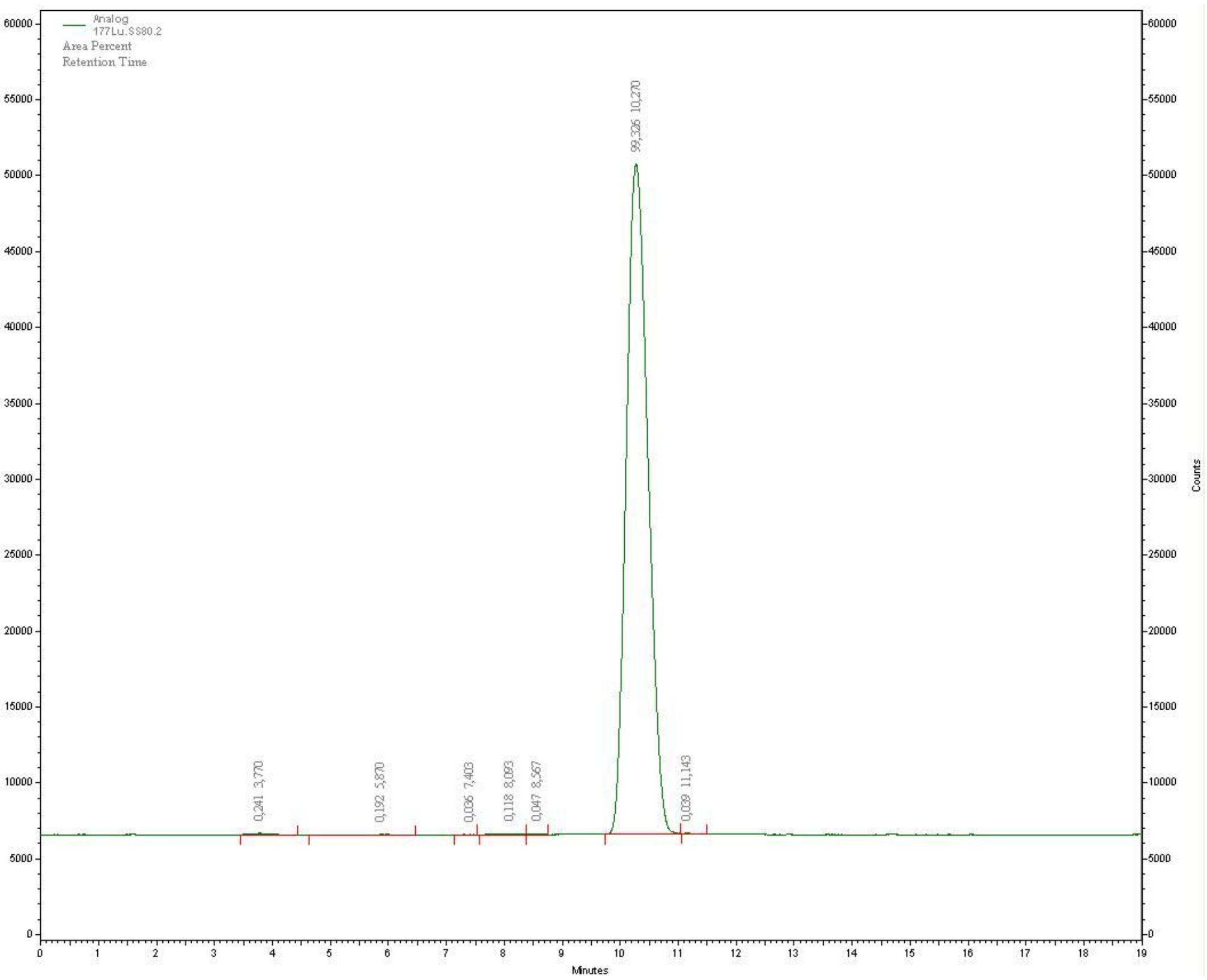

Figure S19: Analytical Radio-HPLC of [^{177}Lu]Lu-DOTAGA.(Glu)$_2$.(FAPi)$_2$ **^{177}Lu-18** (t_R = 10.3 min) after radiolabeling with 10 nmol **18**, and 50 MBq ^{177}Lu in 1 M HEPES (pH = 5.5), 20-50% MeCN + 0.1% TFA (linear gradient) in 20 min, 1 mL/min.

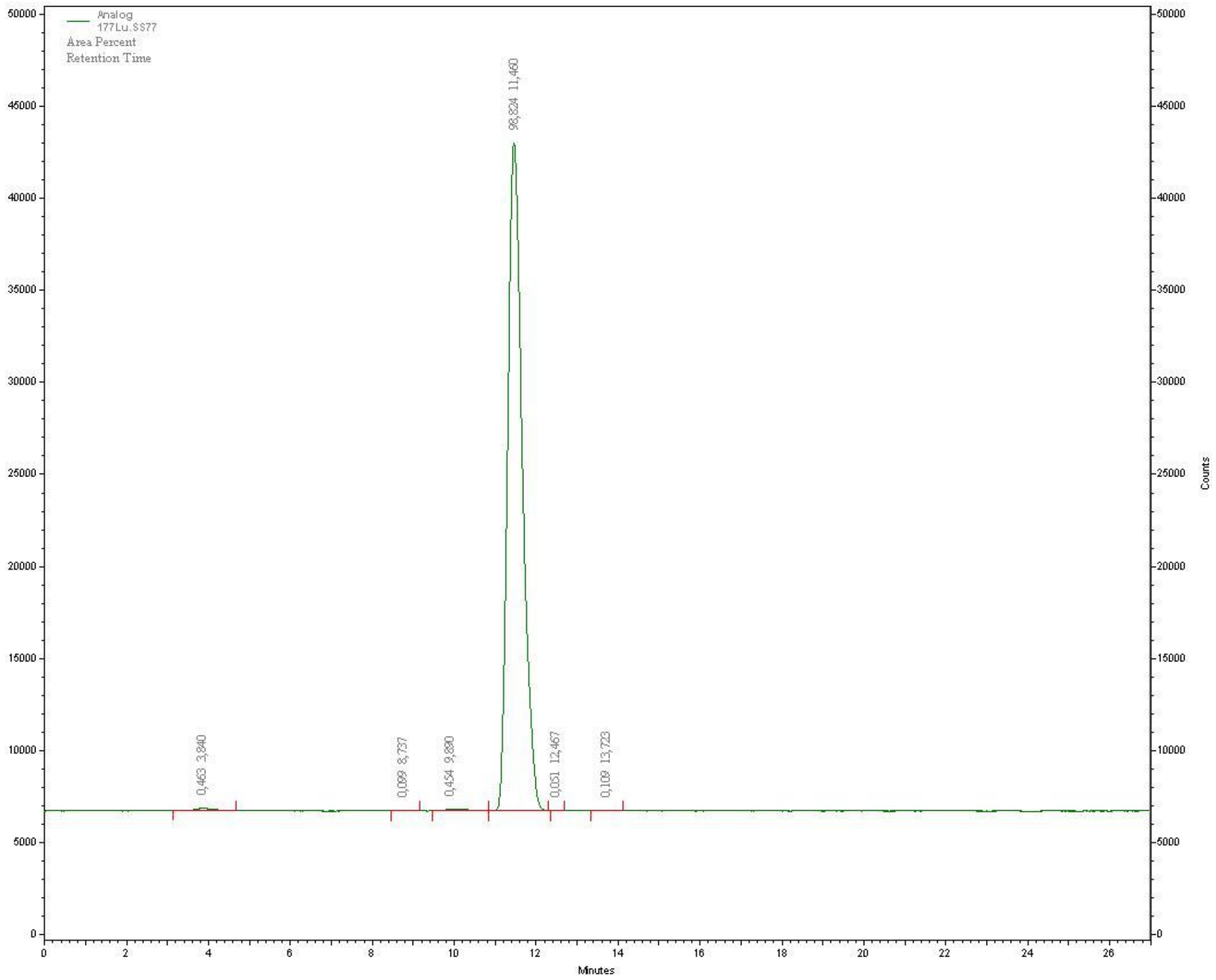

Figure S20: Analytical Radio-HPLC of [^{177}Lu]Lu- DOTAGA.PEG$_2$.Glu.(FAPi)$_2$ **^{177}Lu-25** (t_R = 11.5 min) after radiolabeling with 10 nmol **25**, and 50 MBq ^{177}Lu in 1 M HEPES (pH = 5.5), 20-50% MeCN + 0.1% TFA (linear gradient) in 30 min, 1 mL/min.

Complex Stabilities

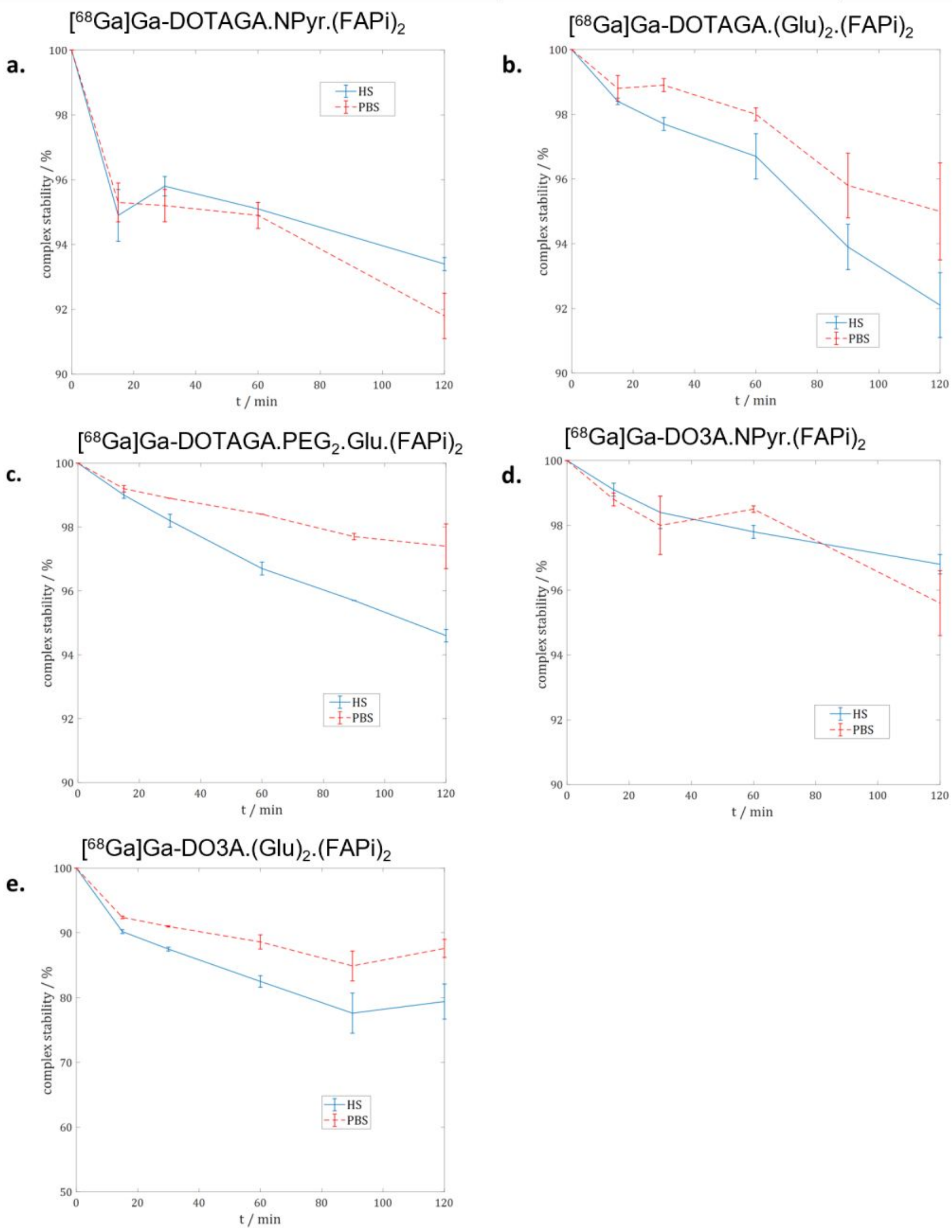

Figure S21: Complex stability of the ^{68}Ga-labeled radioligands in human serum (HS) and phosphate-buffered saline (PBS) over 120 minutes, respectively (n = 3).

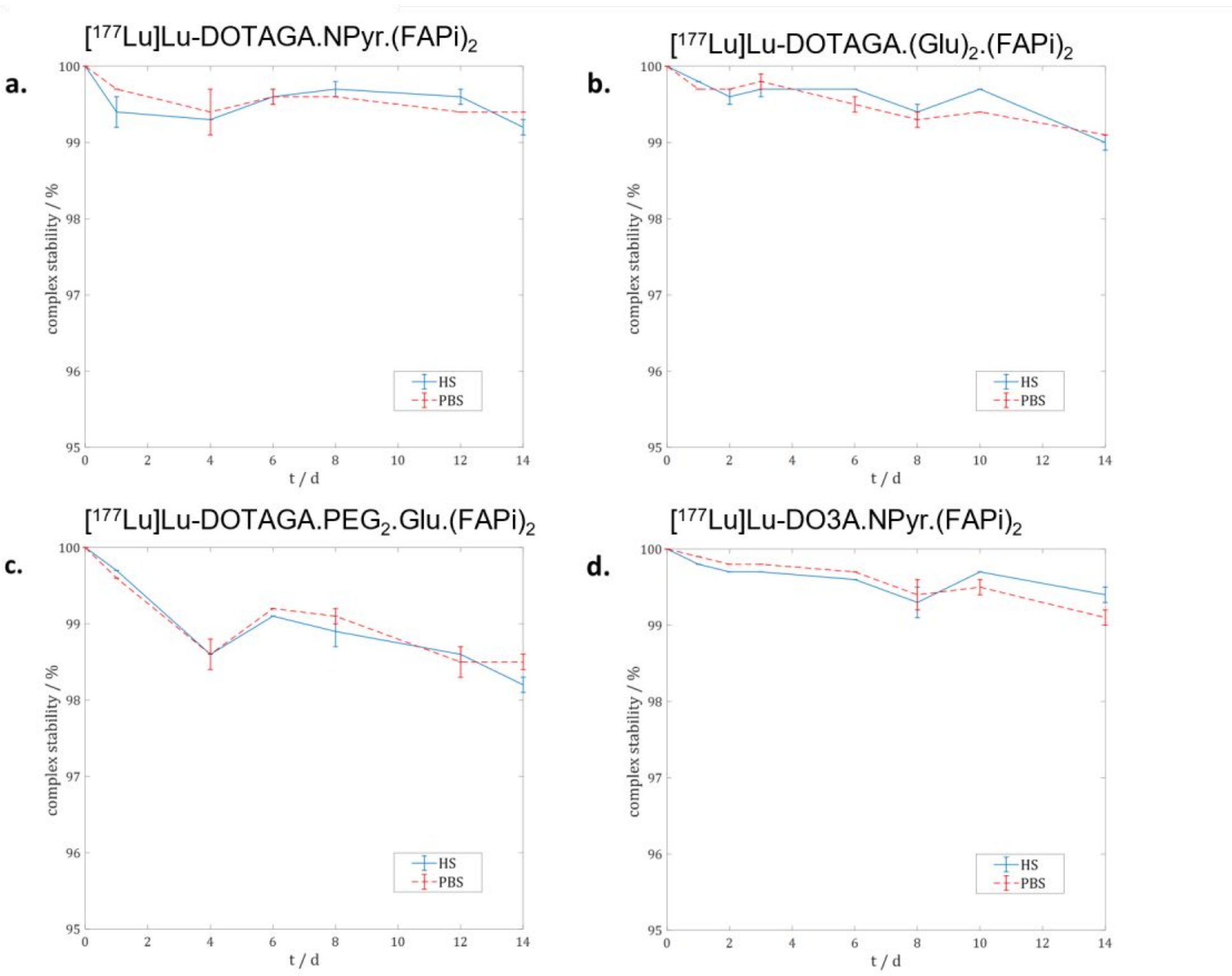

Figure S22: Complex stability of the ^{177}Lu-labeled radioligands in human serum (HS) and phosphate-buffered saline (PBS) over 14 days, respectively (n = 3).

In vitro-inhibition assays

Table S1: Selectivity index (SI = IC_{50}(DPP8 or DPP9)/IC_{50}(FAP)) of different FAP inhibitors.

**Compound**	**SI**	**SI**
	**DPP8/FAP**	**DPP9/FAP**
DOTAGA.NPyr.(FAPi)$_2$ (**8**)	210	700
DO3A.NPyr.(FAPi)$_2$ (**11**)	440	420
DOTAGA.(Glu)$_2$.(FAPi)$_2$ (**18**)	80	480
DO3A.(Glu)$_2$.(FAPi)$_2$ (**19**)	240	420
DOTAGA.PEG$_2$.(FAPi)$_2$ (**25**)	60	160

Table S2: Comparison of FAPi monomers, 1st generation, 2nd generation and 3rd generation FAPi homodimers

Complex	$LogD_{octanol/PBS}$	% Protein Binding (30 min)	% Protein Binding (60 min)	K_d (nM)	B_{max} (nM)
**FAPi monomers**					
[^{68}Ga]Ga-DOTA.SA.FAPi	−3.38 ± 0.03	10.6 ± 3.9		0.8 ± 0.22	1.6 ± 0.53
[^{177}Lu]Lu-DOTA.SA.FAPi	−2.86 ± 0.06	9.9 ± 4.4		0.4 ± 0.03	0.7 ± 0.08
**1st Generation FAPi homodimers**					
[^{68}Ga]Ga-DOTAGA.(SA.FAPi)$_2$	−1.83 ± 0.02	18 ± 1.1		1.2 ± 0.26	0.5 ± 0.03
[^{177}Lu]Lu-DOTAGA.(SA.FAPi)$_2$	−1.71 ± 0.03	25.3 ± 0.8		1.35 ± 0.7	0.4 ± 0.05
**2nd Generation FAPi homodimers**					
[^{68}Ga]Ga-DOTAGA.Glu.(FAPi)$_2$	−2.90 ± 0.10	9.4 ± 2.2	6.3 ± 0.4	0.8±0.2	0.7±0.04
[^{177}Lu]Lu-DOTAGA.Glu.(FAPi)$_2$	−3.00 ± 0.10	9.2 ± 0.03	7.4 ± 0.5	0.7±0.1	0.4±0.02
[^{68}Ga]Ga-DO3A.Glu.(FAPi)$_2$	−2.20 ± 0.04	9.6 ± 0.2	6.3 ± 0.3	0.9±0.3	0.5±0.05
[^{177}Lu]Lu-DO3A.Glu.(FAPi)$_2$	−1.70± 0.01	27.7 ± 0.9	28.3 ± 1.8	0.7±0.1	0.4±0.02
**3rd Generation FAPi homodimers**					
[^{68}Ga]Ga-DOTAGA.(Glu)$_2$.(FAPi)$_2$	−3.49 ± 0.25	11.3 ± 0.4	8.7 ± 0.4	1.1 ± 0.3	0.9 ± 0.08
[^{68}Ga]Ga-DOTAGA.PEG$_2$.(FAPi)$_2$	−3.04 ± 0.28	8.4 ± 0.6	6.5 ± 0.3	1.2 ± 0.4	1.2 ± 0.12

Experimental Section

Chemicals and equipment

All chemicals were purchased from Sigma-Aldrich and Merck (Darmstadt, Germany), TCI (Eschborn, Germany), ABCR (Karlsruhe, Germany), Alfa Aesar by Thermo Fisher Scientific (Kandel, Germany), Acros Organics (Schwerte, Germany) and VWR (Bruchsal, Germany). Dry solvents were bought from Acros Organics, Alfa Aesar by Thermo Fischer Scientific. Merck as well as deuterated solvents from Deutero (Kastellaun, Germany). The chelators DOTA-tri(^{t}Bu)ester (2-(4,7,10-tris(2-(tert-butoxy)-2-oxoethyl)-1,4,7,10-tetraazacyclododecan-1-yl)acetic acid) and DOTA-GA(^{t}Bu)$_4$ (5-(tert-butoxy)-5-oxo-4-(4,7,10-tris(2-(tert-butoxy)-2-oxoethyl)-1,4,7,10-tetra-azacyclododecan-1-yl)pentanoic acid) were obtained from CheMatech (Dijon, France). (S)-6-(4-Aminobutoxy)-N-(2-(2-cyano-4,4-difluoropyrrolidin-1-yl)-2-oxoethyl)quinoline-4-carboxamide, trifluoroacetate salt (FAPi-NH_2*TFA) was purchased from KE Biochem Co. (Shanghai, China).

The human serum was commercially available by Sigma (H4522). All culture reagents were from Gibco BRL, Life Technologies (Grand Island, NY). The human prostate adenocarcinoma cell line PC3 (CLS-300312, Lot number 816SF) and cancer associated prostate fibroblast cell line (hTERT PF179T, ATCC CRL-3290, Lot Number 0303) were obtained from Cell Lines Service GmbH (CLS, Eppelheim, Germany) and the American Type Culture Collection (ATCC, Manassas, Virginia), respectively.

Thin-layer chromatography (TLC) plates coated with silica gel 60 F254 from Merck were used. For analysis they were investigated via UV lamp (λ=254 and 366 nm) as well as staining with potassium permanganate. Silica 60 (0.040-0.063 mm particle size) was used for column chromatography.

Nuclear magnetic resonance (NMR) spectra were recorded in deuterated solvents on an Avance II 400 spectrometer (400 MHz, 5 mm BBFO sample head with z-gradient and ATM, SampleXPress 60 sample changer). MestReNova 14.2.0 software from Mestrelab Research (Santiago de Compostela, Spain) was used to analyze the spectra.

Liquid chromatography coupled with electrospray ionization mass spectrometry (ESI-LC/MS) was performed using a LC/MS system from Shimadzu (Japan) with CBM-20A system controller, SIL-20A HT autosampler, two LC-20AD pump modules, CTO-20 AC column oven. The UV signal was measured with SPD-M20A photodiode array detector and the mass spectrum with a LCMS-2020 mass

spectrometer. The separation was realized with a Eurosphere II 100-5 C18 column (150x4 mm) from Knauer (Berlin, Germany) and linear gradient of acetonitrile (MeCN)/water (HPLC grade) + 0.1% formic acid.

High-pressure liquid chromatography coupled with electrospray ionization mass spectrometry (ESI-HPLC-MS) measurements were performed using an Agilent Technologies G6545A Q-ToF with electron spray ionization coupled to a 1260 Infinity II HPLC system (Agilent Technologies, Santa Clara, USA) with G7111B 1260 Quaternary Pump, G7129A 1260 Vial Sampler, and G7116A Multicolumn Thermostat. Separation was performed using an Agilent Poroshell 120 EC-C8 column (2.1x100 mm, 2.7 µm) with H_2O + 2% MeCN / MeCN + 2% H_2O + 0.05% HFo (Formic acid; mobile phase additive, typically to improve peak shape and resolution) and a flow rate of 0.1 mL/min.

Semi-preparative reversed-phase high-pressure liquid chromatography (RP-HPLC) was performed using LaChrom HPLC (7000 series) from Merck Hitachi with L-7100 pump, L-7400 UV detector (λ = 254 nm), a D-7000 interface and autosampler. Separation was performed using a Phenomenex (Aschaffenburg, Germany) Synergi Max-RP C18 column (250x10 mm, 4 µm) and linear gradient of H_2O + 0.1% TFA (mobile phase A) and MeCN + 0.1% TFA (mobile phase B) with a flow rate of 5 mL/min.

For radiolabeling only chemicals of the highest purity (TraceSelect if available) were used. Radio-TLCs were developed using 0.1 M or 0.25 M citrate buffer (pH = 4.0). Additional TLCs were developed using 1 M ammonium acetate (AmOAc, pH = 4.0)/methanol (MeOH) (1:1). All radio-TLCs were evaluated using a CR-35 Bio Test-Imager from Duerr-ndt (Bietigheim-Bissingen, Germany) and AIDA software from Elysia-Raytest (Straubenhardt, Germany). Analytical radio-HPLC was performed also using a Merck Hitachi LaChrom HPLC (7000 series). Separation was performed using Phenomenex Luna C18 column (250x4.6 mm, 5 µm) and linear gradient of H_2O + 0.1% TFA (mobile phase A) and MeCN + 0.1% TFA (mobile phase B) with a flow rate of 1 mL/min. The radio-HPLC is equipped with a Ramona analog radio detector from Elysia-Raytest. Its energy window was set to 100-1200 keV for ^{68}Ga and 100-250 keV for ^{177}Lu.

Quantitative γ-counting was performed with a COBRA II γ-system well counter from Packard Instrument (USA).

For the experiments for the cell and animal studies the GalliaPharm® Ge-68/Ga-68 generator was available from Eckert & Ziegler (Berlin, Germany) along with the Modular-Lab PharmTracer module by Eckert & Ziegler (Berlin, Germany). The quality control of the precursor and the radiotracers was performed by an analytical Reverse-Phase High Performance Liquid Chromatography (RP-HPLC) on an analytical Nucleosil 120-5 column C18 (250 x 4.5 mm) applying a linear gradient of 15-90% solvent B in 30 min at a flow rate of 1 mL/min. (solvent A, 0.1% trifluoroacetic acid (TFA)/water (H_2O); solvent B, 0.1%TFA/acetonitrile (MeCN). Ultraviolet detection was performed using an Agilent detector at 214 nm. For radioactivity measurement, a Na(TI) well-type scintillation Gina star was used. The radiotracer solutions for the experiments were prepared by dilution with 0.9% NaCl (Bichsel AG, Interlaken, Switzerland).

For µPET/CT studies, a dedicated micro-PET/SPECT/CT scanner (Albira Si; Bruker Biospin, Ettlingen, Germany) was used.

[^{177}Lu]$LuCl_3$ in 0.04 M HCl was purchased from ITM (Isotope Technologies Munich SE, Munich, Germany).

Radiolabeling

^{68}Ga-radiolabeling

Each of the precursor **8**, **11**, **18**, **19** or **25** (2–20 nmol, 2–20 µL 1 mM stock solution) was added to 400 µL buffer. Three different buffer systems were used: 1 M 4-(2-hydroxyethyl)-1-piperazineethansulfonic acid (HEPES pH = 4.5 or 5.5), 1 M ammonium acetate (AmOAc, pH = 4.5) and 1 M sodium acetate (NaOAc, pH = 4.5). Finally, 50 MBq ^{68}Ga (purified generator eluate) was added and the reaction was carried out at 95 °C. Reaction controls were taken at different time points to investigate reaction kinetics and analyzed via radio-TLC with 0.1 M citrate buffer (pH = 4.0) as the mobile phase. The ^{68}Ga-labeled compounds were purified manually via a Sep-Pak® C18 Plus Light cartridge immediately after the radiolabeling. The cartridge was pre-conditioned with 2 mL ethanol, followed by 5 mL Milli-Q® water. The reaction mixture was then loaded on the cartridge. The reaction vial was rinsed once with either the used buffer solution or H_2O (TraceSelect) and was also loaded on

the cartridge. After washing the cartridge with 1 mL H_2O (TraceSelect), the purified product was eluted with 1 mL ethanol or 1:1 mixture of ethanol/water.

***In vitro*-inhibition assays (IC_{50} measurements)**

Briefly, Z-Gly-Pro-7-amino-4-methylcoumarine (AMC, Bachem) at a final concentration of 50 µM at pH = 8.0 in 0.05 M Tris-HCl buffer, 1 mg/mL BSA and 140 mM NaCl was used as the substrate for FAP. N-succinyl-Gly-Pro-AMC (Bachem) was used for PREP. Here, a final concentration of 250 µM at pH = 7.4 in 0.1 M K-phosphate, 1 mM EDTA, 1 mM DTT and 1 mg/mL BSA buffer was used. IC_{50} measurements for DPP4, DPP8 and DPP9 were performed using Ala-Pro-pNA as the substrate at the respective concentrations of 25 µM (DPP4), 300 µM (DPP8) and 150 µM (DPP9) at pH 7.4 in 0.05 M HEPES-NaOH buffer with 0.1% Tween-20, 0.1 mg/mL BSA and 150 mM NaCl).

The FAP inhibitors were pre-incubated with the enzyme for 15 minutes at 37 °C, afterwards the substrate was added and the velocities of AMC release were measured kinetically at λ_{ex}= 380 nm and λ_{em}= 465 nm (405 nm for pNA) for at least 10 minutes at 37 °C. At least eight concentrations for each inhibitor were tested and the measurements were carried out in triplicates. The final DMSO concentration was constant (0.1% (v/v)) during the experiment. The measurements were carried out using an Infinite 200 from Tecan Group Ltd. (Männedorf, Switzerland) and the data were processed using the Magellan software. Data fitting was performed using a non-linear fitting model in Grafit 7.4.
